# Tetrahedral Nitrogen Atoms Arrangement in A‐Site Cations: A New Approach for Regulating Sensitivity and Energy of Perovskite Energetic Materials

**DOI:** 10.1002/advs.202415680

**Published:** 2025-03-24

**Authors:** Shiyong Chen, Yuan Gao, Cheng Dong, Lixiao Shen, Yinning Zeng, Peng Bao, Yan Li, Zhenxin Yi, Houhe Chen, Shunguan Zhu, Lin Zhang

**Affiliations:** ^1^ School of Chemistry and Chemical Engineering Nanjing University of Science and Technology Nanjing 210094 China

**Keywords:** detonation performance, mechanical sensitivity, perovskite energetic materials, thermal decomposition, urotropine

## Abstract

Perovskite energetic materials (PEMs) are emerging combinations of oxidants and reductives, which are promising in explosives owing to the advantages of high energy, simple synthesis and low cost. However, the friction sensitivity of the currently reported PEMs is so high that it limits the further application of PEMs. In this work, a tetrahedral nitrogen‐atom‐arrangement structure, urotropine, is introduced as A‐site cation of PEMs, then four urotropine‐based PEMs ([C_6_H_14_N_4_][M(ClO_4_)_3_], named TAPs) are successfully constructed experimentally for the first time. The crystal structure, reaction progress, thermal decomposition, sensitivity, and detonation performance of TAPs are characterized. The results indicate that, different from the existing cubic PEMs, the crystal structure of TAPs experiences compression along the *c*‐axis, despite the *c*‐axis length being twice that of the *a* or *b*‐axes. As expected, the friction sensitivity is remarkably reduced and the detonation performance is significantly improved. Moreover, the hardness of A‐site cations is proposed as a key factor affecting the impact sensitivity of PEMs, while C─H···O hydrogen bonds play an important role in regulating friction sensitivity. The emergence of TAPs provides a design concept of high‐energy insensitive PEMs and a unique perspective for understanding the mechanical sensitivity of energetic materials.

## Graphical abstract

The urotropine is selected as the A‐site cation in PEMs, and four urotropine‐based PEMs ([C_6_H_14_N_4_][M(ClO_4_)_3_]) are successfully prepared. Compared with the cubic PEMs, the crystal structure of the urotropine‐based PEMs has an expansion along the *c*‐axis. They are endowed with more excellent fiction sensitivity and detonation performance than corresponding DAPs ([C_6_H_14_N_2_][M(ClO_4_)_3_]).

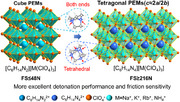

## 1 Introduction

Explosives not only play a crucial role in military and civilian fields, but also bring huge economic benefits.^[^ ^1^, ^2^ ^]^ Ever since the discovery of 1,2,3‐propanetriol trinitrate, researchers have focused on designing and synthesizing explosives with high energy and low sensitivity.^[^ ^3^, ^4^, ^5^ ^]^ Currently, CHON‐based explosives, such as 1,3,5‐trinitro‐1,3,5‐triazinane (RDX) and 2,4,6,8,10,12‐hexanitro‐2,4,6,8,10,12‐hexaazaisowurtzitane (CL‐20), continue to dominate modern weapon charges and civil fields.^[^ ^6^, ^7^ ^]^ Additionally, various molecular construction strategies, such as the rich nitrogen compounds,^[^ ^8^, ^9^ ^]^ energetic ion salts,^[^ ^10^ ^]^ energetic metal–organic frameworks,^[^ ^11^ ^]^ energetic cocrystals,^[^ ^12^, ^13^, ^14^ ^]^ perovskite energetic materials (PEMs) have been reported, expanding options for designing high‐performance explosives.^[^ ^15^ ^]^

PEMs with general formula ABX_3_ are a class of energetic compounds structurally similar to perovskites. In this structure, the A‐site is occupied by an organic amine cation, the B‐site by an inorganic cation, and the X‐site by an oxidative anion (ClO_4_^−^, IO_4_^−^ or NO_3_^−^).^[^ ^16^ ^]^ As a novel combination of oxidants and reductives, PEMs have the advantages of simple synthesis, low cost, compact structure, good regulation, and fast deflagration to detonation process, making them to be a unique platform for the design of energetic materials (EMs) with diverse functions.^[^ ^17^, ^18^ ^]^ However, the reported PEMs containing ClO_4_⁻ at the X‐site exhibit high friction sensitivity (≯60 N).^[^ ^16^ ^]^ During handling, transportation and storage, PEMs may experience physical effects such as impaction and friction. Reduced friction sensitivity enhances the stability of PEMs during handling and transportation, enabling transport under harsher environmental conditions and less stringent requirements for transportation equipments.

**Figure 1:**
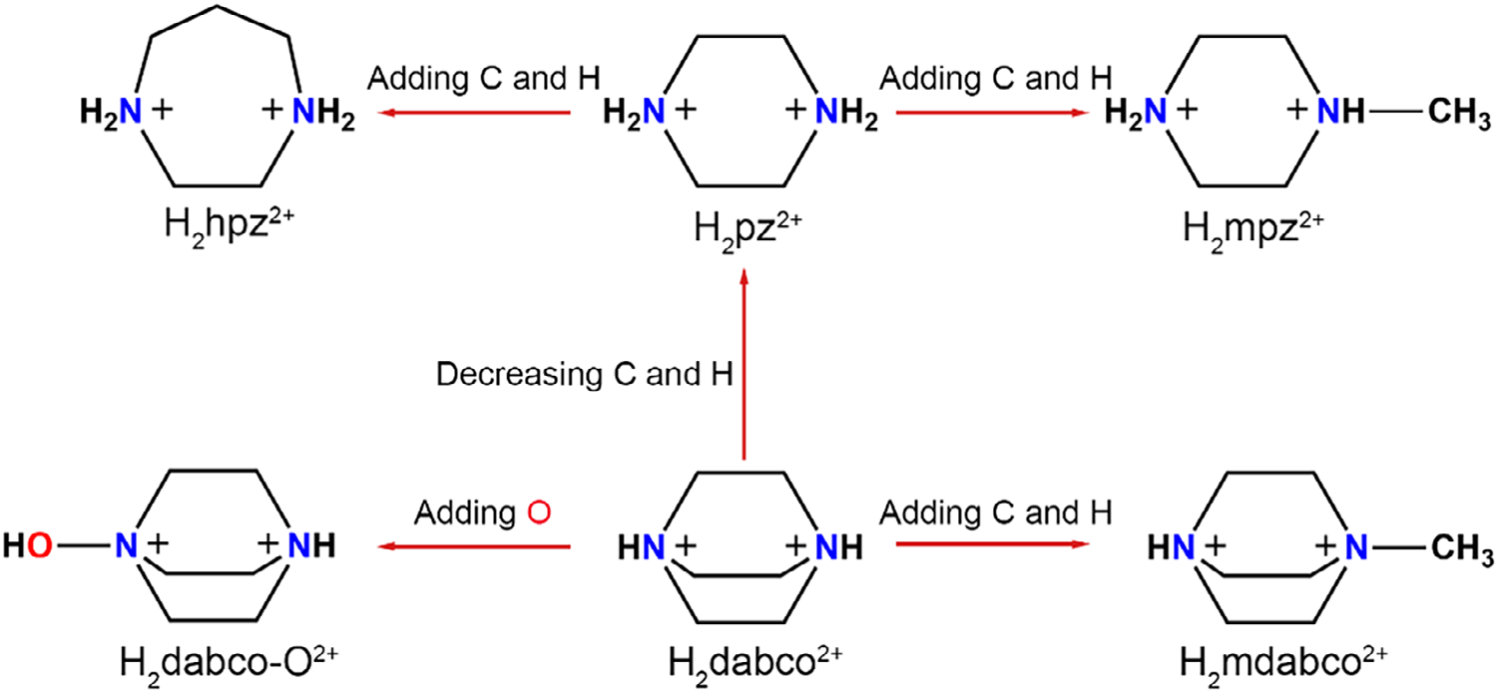
The relationship between reported A‐site cations.

A‐site cations, as reductive component, influence the thermal stability, sensitivity, detonation performance of PEMs. Traditionally, the oxygen balance, energy and sensitivity of PEMs can be manipulated by changing the content of C, O, and H of A‐site cations. However, high energy and low sensitivity of PEMs can hardly achieve simultaneously.^[^ ^16^ ^]^ Due to the size constrain of the external frameworks, the reported A‐site cations (**Figure** **1**) include only piperazine‐1,4‐diium (H_2_pz^2+^), 1,4‐diazabicyclo[2.2.2]octane‐1,4‐diium (H_2_dabco^2+^) and their derivatives.^[^ ^19^ ^]^ Given these limitations, adjusting the nitrogen content of A‐site cations might be a new strategy to regulate the energy and sensitivity of PEMs. Increasing of nitrogen content could enhance the enthalpy of formation and improve the oxygen balance of compounds.^[^ ^20^, ^21^ ^]^ At the same time, the increased nitrogen content contributes to relatively stronger intermolecular interactions, ultimately reducing the sensitivity.^[^ ^22^, ^23^, ^24^ ^]^

Therefore, 1,3,5,7‐tetraazatricy‐clo[3.3.1]decane (urotropine, Tazcd, C_6_H_12_N_4_), a precursor for synthesizing RDX and HMX, is included in the investigation.^[^ ^25^ ^]^ Compared with piperazine (Pz, C_4_H_10_N_2_) and 1,4‐diazabicyclo[2.2.2]octane (Dabco, C_6_H_12_N_2_), Tazcd contains twice as many N as Dabco and Pz. Additionally, and the number of C─N bonds in Tazcd is twice of that in Dabco and three times of that in Pz. Furthermore, the carbon and hydrogen content in Tazcd is equivalent to that in Dabco and greater than that in Pz. Therefore, Tazcd would lead to the higher enthalpy of formation and crystal density of EMs. The four N in Tazcd are arranged tetrahedrally, potentially causing structural changes in PEMs. Tazcd has been utilized to prepare a high hardness perovskite ([C_6_H_14_N_4_]NH_4_Br_3_), demonstrating its feasibility for preparation of PEMs.^[^ ^26^, ^27^ ^]^ However, the hydrolysis of Tazcd under acidic conditions introduces significant uncertainty and challenges in the preparation of new PEMs.^[^ ^28^ ^]^

In this study, the tolerance factor was initially employed to assess the compatibility of 1,3,5,7‐tetraazatricy‐clo[3.3.1]decane‐1,3‐diium (H_2_tazcd^2+^) as an A‐site cation. Subsequently, a promising method was developed to prepare urotropine‐based PEMs, including (C_6_H_14_N_4_)[M(ClO_4_)_3_] (M = Na^+^, K^+^, Rb^+^, and NH_4_^+^ for TAP‐1, TAP‐2, TAP‐3, and TAP‐4, respectively. More naming information can be found in Support Infoamation). In addition, the reaction of TAPs was monitored by ReactRaman to detect the hydrolysis of Tazcd. The crystal structure, thermal decomposition, sensitivity and detonation performances of TAPs were characterized. Finally, the mechanism by which A‐site cations influence the mechanical sensitivity of PEMs was proposed.

## 2 Results and Discussion

### 2.1 Crystal Structure

**Figure 2:**
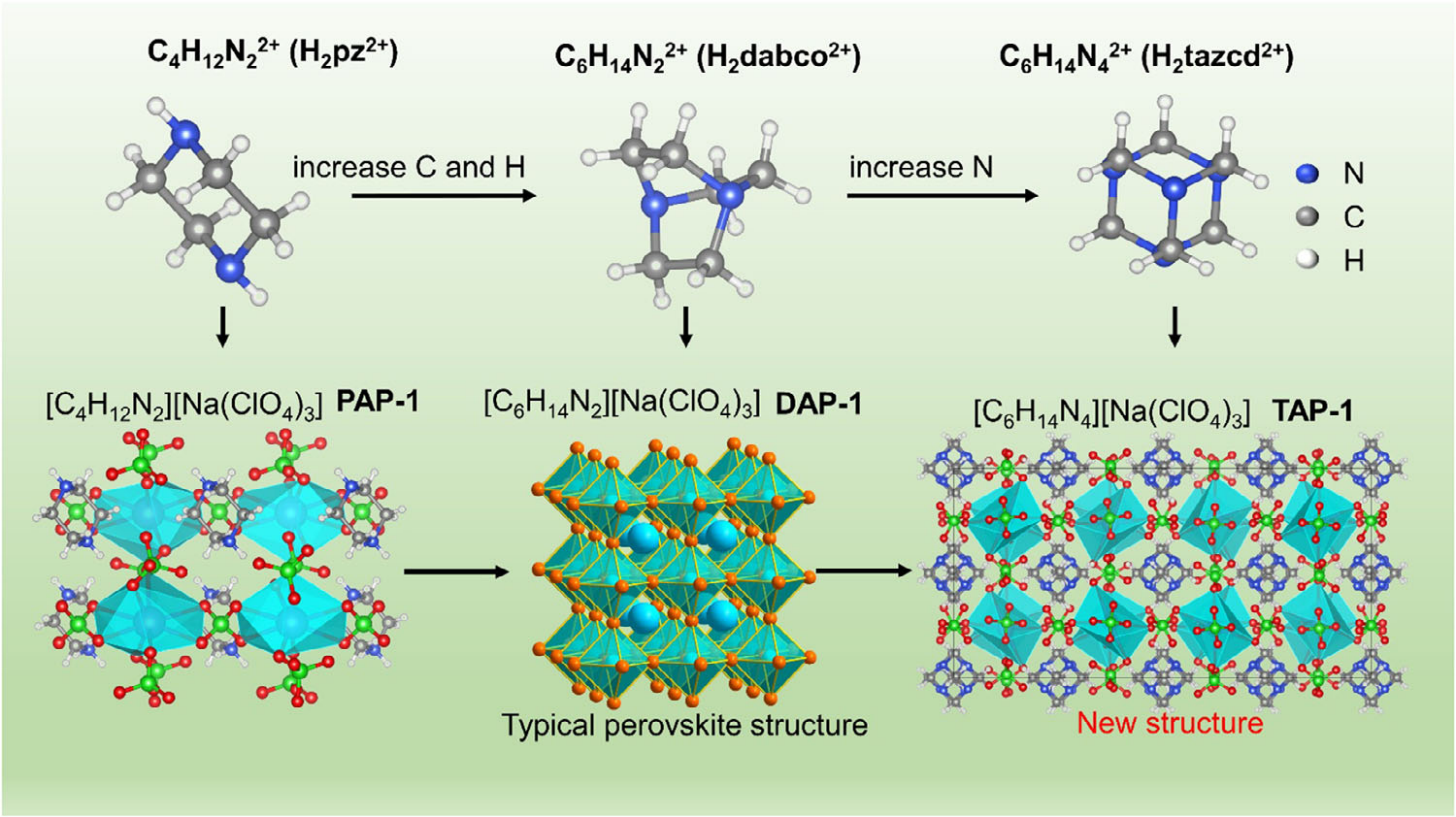
Structural comparison of the PEMs caused by the changes in A‐site ions along *b*‐axis.

The size and structure of A‐site cations are crucial factors causing the structure change of PEMs. As shown in **Figure** **2**, the H_2_dabco^2+^ cations are positioned in the center of the framework [Na(ClO_4_)_3_]^2−^ in [C_6_H_14_N_2_][Na(ClO_4_)_3_] (referred to as DAP‐1).^[^ ^15^ ^]^ In contrast, the H_2_pz^2+^ cations are located at the edge of the framework.^[^ ^29^ ^]^ The H_2_dabco^2+^ contains two more C and two more H atoms than H_2_pz^2+^, requring more space. Consequently, the octahedral cavities at the center of the framework are more suitable for H_2_dabco^2+^. In contrast, the smaller H_2_pz^2+^ cations tend to occupy the edges of the framework to support the framework of [Na(ClO_4_)_3_]^2−^. The four N atoms in H_2_tazcd^2+^ occupy the four vertices of the N‐tetrahedron, rather than two ends of the skeleton in H_2_dabco^2+^ and H_2_pz^2+^. This unique arrangement of N induces novel structural changes in PEMs. Although the hydrolysis of Tazcd in the acidic environment is a severe challenge, the urotropine‐based PEMs still have been successfully synthesized. Compared to DAP‐1, the unit cell length of urotropine‐based PEMs along the *c*‐axis was approximately twice that of the *a* or *b* axes. From a crystallographic perspective, TAPs with a tetragonal structure exhibited compression along the *c*‐axis compared to DAP‐1, which had a cubic structure.

Structure determination results indicated that TAP‐1 crystallized in tetragonal space group I4_1_/a with crystal density of 1.99 g cm^−3^ at 100 K. Although TAP‐1 shared the same molecular formula (ABX_3_) with DAP‐1 and PAP‐1, it had undergone significant structural changes. The previously reported DAP‐1 belongs to the cubic crystal system. In contrast, TAP‐1 belonged to the tetragonal crystal system with reduced crystal symmetry, where the length of the unit cell along *c*‐axis was twice that of either *a*‐axis or *b*‐axis. Each sodium ion was surrounded by six perchlorate anions, forming an octahedron. These octahedrons extended in three directions to create the framework structure of [Na(ClO_4_)_3_]^2−^. The H_2_tazcd^2+^ cations were distributed within the octahedral cavities. TAP‐2 and TAP‐4 also crystallized in tetragonal space group I4_1_/a, with crystal density of 2.09 and 1.97 g cm^−3^ at 100 K, respectively.

As expected, the crystal densities of TAP‐2 and TAP‐4 were higher than those of DAP‐2 ([C_6_H_14_N_2_][Na(ClO_4_)_3_], 2.03 g cm^−3^) and DAP‐4 ([C_6_H_14_N_2_][NH_4_(ClO_4_)_3_], 1.91 g cm^−3^), respectively.^[^ ^15^, ^19^ ^]^ However, the crystal density of TAP‐1 was lower than that of DAP‐1 (2.02 g cm^−3^). It is well known that PEMs consist of three parts, and the crystal density of PEMs is highly related to the tightness of the these components, the tighter the components, the higher the density. According to the Hard–Soft‐Acid–Base (HSAB) theory,^[^ ^30^ ^]^ C_6_H_14_N_4_^2+^ is the softer acid compared to C_6_H_14_N_2_^2+^, resulting weaker electrostatic attraction with the framework of [Na(ClO_4_)_3_]^2−^. Consequently, the distance between Na^+^ and ClO_4_^−^ is greater than that in DAP‐1. Experimental results also indicate that the Na─O bonds length in TAP‐1 (2.94–3.02 Å) is longer than that in DAP‐1 (2.876 Å), which is consistent with the aforementioned theory.

**Figure 3:**
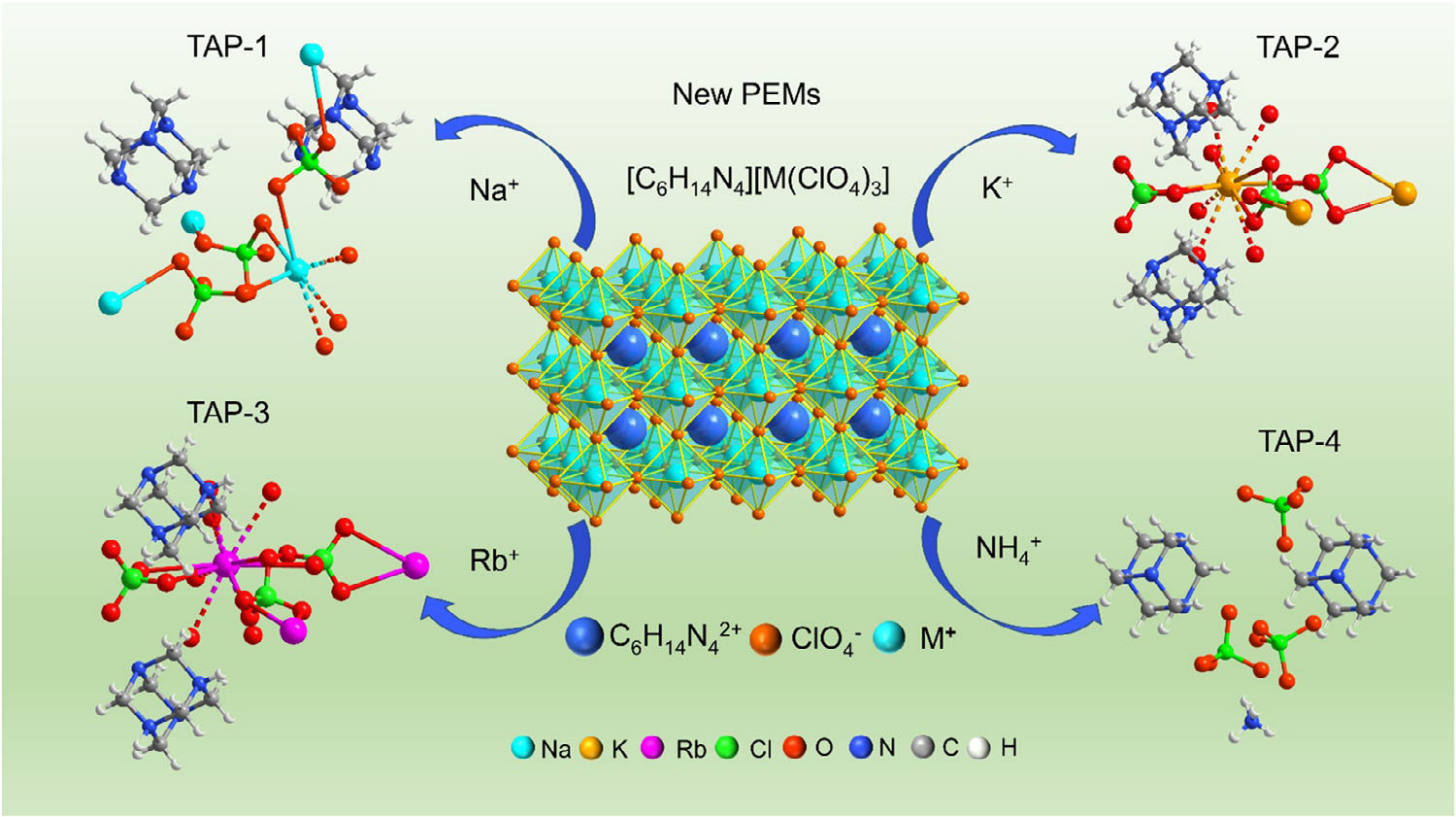
The asymmetrical units of different TAPs.

Different B‐site cations in TAPs existed in varying environments. As illustrated in **Figure** **3**, Na^+^ formed coordination bonds with five neighboring O atoms in TAP‐1. In contrast, K^+^ in TAP‐2 was situated in a twelve‐coordinated environment, with K─O bond lengths ranging from 2.92 to 3.19 Å. In TAP‐4, two independent N···Cl distances were observed: 3.654 and 3.637 Å.

The single crystal of TAP‐3 could not be obtained experimentally. Nevertheless, powder X‐ray diffraction (PXRD) patterns indicated that the main peak positions of TAP‐3 were closed to those of other TAPs (Figure S3a, Supporting Information). It is believed that TAP‐3 and TAP‐2 should possess similar structures. Finally, the structure of TAP‐3 was obtained based on the density functional theory (more details can be found in Support Information). The results indicated that TAP‐3 crystallized in tetragonal space group I4_1_/a with crystal density of 2.22 g cm^−3^. Rb^+^ was surrounded by twelve O atoms, forming Rb─O coordination with bond lengths ranging from 3.089 to 3.192 Å (Figure S3d, Supporting Information).

### 2.2 In Situ Reaction Progress with ReactRaman

**Figure 4:**
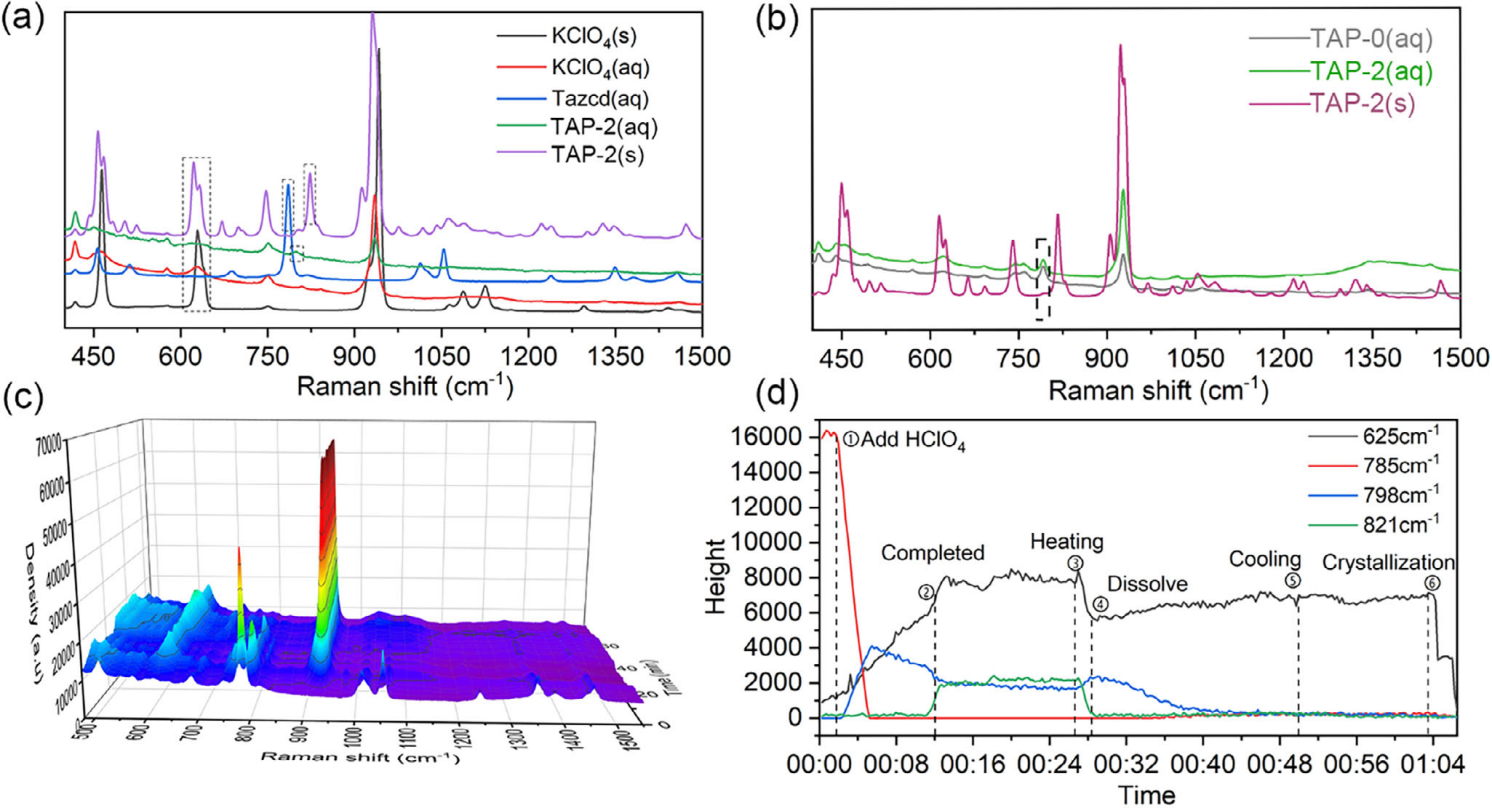
a) Raman spectra of the substances existed in the reaction process; b) Comparison of Raman spectra of TAP‐0, TAP‐2(aq) and TAP‐2(s) and c) 3D spectra surface of the complete reaction progress at the range of 480–1500 cm^−1^; d) Trends of characteristic peaks at 625, 785, 798, and 821 cm^−1^.

Due to the hydrolysis of Tazcd in acidic environment, ReactRaman was utilized to track the content change of reactants, intermediates, and products in both solid and liquid phases during the reaction process,^[^ ^31^ ^]^ as illustrated by the representative results for TAP‐2 in **Figure** **4**. First, the Raman spectra of the substances existed during the reaction process were compared (Figure 4a). The characteristic peaks of TAP‐2_(s)_, TAP‐2_(aq)_ and Tazcd_(aq)_ were at 821, 798, and 785 cm^−1^, respectively. At 625 cm^−1^, a peak overlap occurred between TAP‐2 and KClO_4_, indicating the presence of ClO_4_^−^. The 3D‐Spectra surface was shown in Figure 4b,c, and the trends of the four aforementioned peaks were illustrated in Figure 3d.

As shown in Figure 3d, the content of Tazcd rapidly decreased with the gradual dropwise addition of HClO_4_. Simultaneously, the trends at 798 and 625 cm^−1^ gradually increased, indicating the generation of TAP‐2 and the rise of ClO_4_^−^ in the solution, respectively. The curve at 798 cm^−1^ reached its maximum value at 05:27 and then began to decrease. However, the curve of 821 cm^−1^ remained unchanged until 10:57. Interestingly, the maximum value of the blue curve corresponded to be ≈1/3 amount of HClO_4_. In conjunction with Raman spectra of [C_6_H_13_N_4_]ClO_4_·xH_2_O (TAP‐0) and TAP‐2 in solution (Figure 4b), the reaction process of TAP‐2 has been proposed.

(1) $$C_{6}H_{12}N_{4}+\mathrm{HCI}O_{4}+nH_{2}O\to \left[C_{6}H_{13}N_{4}\right]\left(\mathrm{CI}O_{4}\right)\cdot nH_{2}O$$

(2) $$\begin{array}{ccc} & & \left[C_{6}H_{13}N_{4}\right]\left(\mathrm{CI}O_{4}\right)\cdot nH_{2}O+2\mathrm{HCI}O_{4}\\ & & \;+\;K^{+}\to \left[C_{6}H_{14}N_{4}\right]\left[K{\left(\mathrm{CI}O_{4}\right)}_{3}\right]+H^{+} \end{array}$$

Initially, Tazcd rapidly reacted with HClO_4_ to form TAP‐0 with the addition of HClO_4_. Subsequently, the solution would contain suitable ions meeting the requirements of ABX_3_ when the dropwise addition of HClO_4_ exceeded 1/3, resulting in the reaction of TAP‐0 with excessed HClO_4_ and K^+^ to form TAP‐2. Following this, a substantial amount of TAP‐2 was observed to form in the solution. This is evidenced in by the variation in the curve during the time range of 10:57–13:20. Notably, the increase rate of the curve at 625 cm^−1^ was significantly greater than observed previously. The peak intensity at this time point increased due to the presence of a large number of solids. At 17:16, the amount of TAP‐2 crystals further increased, remaining stable until the solution was heated. To accelerate the hydrolysis process, the solution was heated from 25 to 60 °C. Following the heating, the solids dissolved rapidly. The dissolution process was observed during the time range of 26:37–28:23, during which the trend at 798 cm^−1^ increased, while the trend at 821 cm^−1^ descended. TAP‐2 solids dissolved gradually until completely disappeared, indicating Tazcd began to hydrolyze. At 49:57, the clear solution began to be cooled down to 25 °C. During cooling, the solid was observed to crystallize starting at 01:03:29. Meanwhile, no change was observed in other trends, except for the trend at 625 cm^−1^. This suggestted that no TAP‐2 existed in either solution or solid and that Tazcd was completely hydrolyzed. Based on the observations of the reaction process, it was found that Tazcd undergoes rapid hydrolysis under heating conditions. Therefore, it is essential to maintain room temperature and control the reaction duration during preparation.

### 2.3 Thermal Stability Analysis

The thermal decomposition process of TAPs was tested using differential scanning calorimetry (DSC) and thermal gravimetric analyzer (TG) at a heating rate of 10 K min^−1^ under nitrogen atmosphere. All samples used for DSC‐TG test had a mass ranging from 0.6 to 0.7 mg. As shown in Figure S11a–d (Supporting Information), the thermal decomposition of all TAPs occured with an onset temperature ranging from 216 –226 °C. Specifically, TAP‐1, TAP‐2, TAP‐3 and TAP‐4 decomposed at ≈226, 216, 218, and 220 °C, respectively.

**Figure 5:**
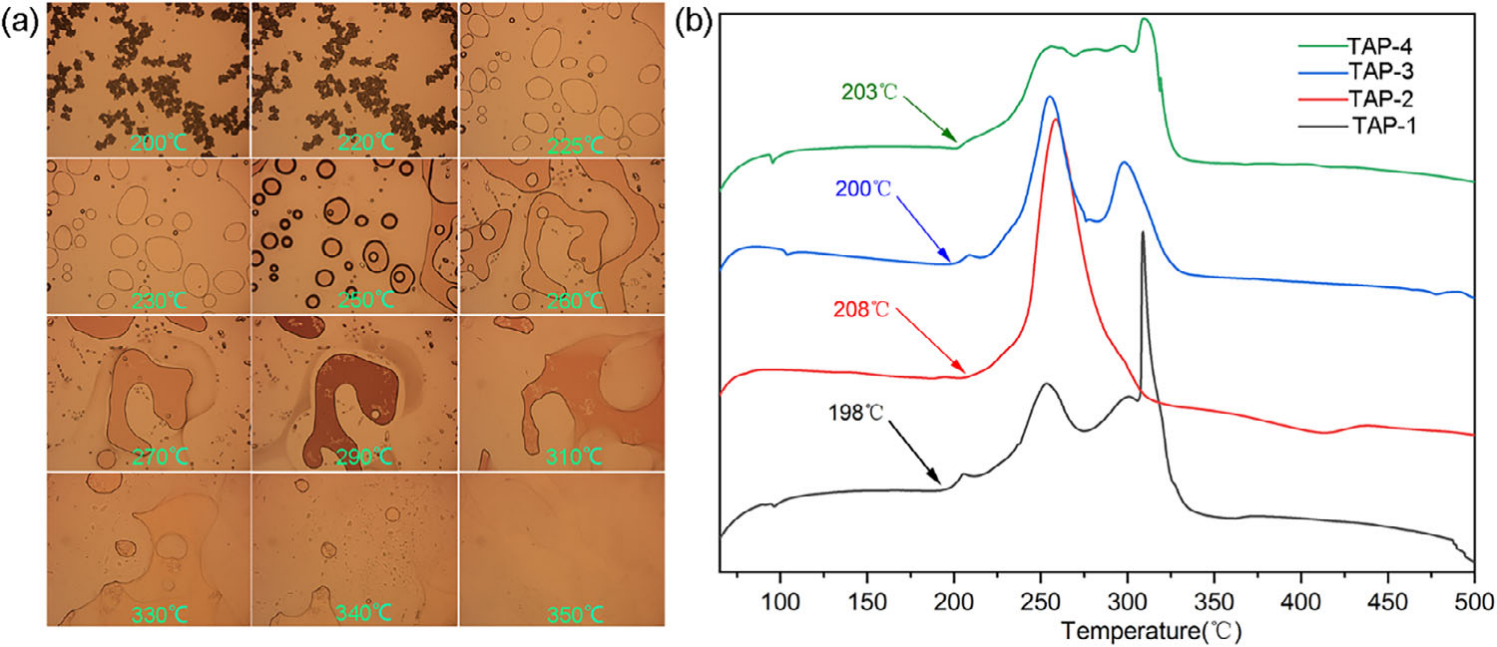
a) The hot stage microscope photos of TAP‐1; b) DSC curves of TAPs.

Interestingly, the decomposition process of TAPs was relatively slow, spanning up to 120 °C. To investigate this extended decomposition process, the entire thermal decomposition of TAP‐1 was observed using a hot stage microscope at a heating rate of 0.2 K s^−1^. As depicted in **Figure** **5**a, the sample exhibited nearly no change, with only a few particles melting before 220 °C. However, the sample instantly melted completely at 225 °C. As the sample temperature continueed to rise, the bubbles became clearly visible in the liquid phase until the end of decomposition, indicating that the decomposition of TAPs i accompanied with melting.

To further confirm the onset decomposition temperature, the mass of all samples used for DSC testing was increased to 1.2–1.3 mg. As shown in Figure 5b, the decomposition process of TAPs was relatively slow. TAP‐1 and TAP‐3 began to melt and decomposed at ≈198 °C. The onset decomposition of TAP‐4 occurred at 203 °C. TAP‐2 exhibited the highest onset decomposition temperature (208 °C) among them. Their initial decomposition temperatures are close to that of RDX (198 °C), which can still meet most military and civilian demands.

### 2.4 Sensitivity Characteristics

From the standpoint of safety and reliability, sensitivity pertains to the safety of products throughout manufacture, transportation, and storage. Impact and friction sensitivities are key parameters that influence the safety of explosives, while flame sensitivity primarily affects initiation reliability.

The comparison of sensitivities between TAPs and other explosives is presented in **Table** **1**. TAPs exhibited unusual sensitivities to stimulus and electrostatic discharge. Specifically, the friction sensitivity values for TAPs exceeded 216 N, significantly higher than that of HMX (120 N). TAP‐4 exhibited a friction sensitivity value of 214 N. Among these PEMs, TAP‐2 was markedly insensitive to friction stimulation, with a friction sensitivity value of 324 N. Although TAPs (4‐8 J) did not demonstrate the expected insensitivity to impact stimulation, the impact sensitivity value of TAP‐2 was close to that of HMX (7.4 J), the others were close to that of CL‐20 (4J).^[^ ^32^ ^]^ The ignition threshold of TAPs was also assessed based on the flame sensitivity. The results indicated that the ignition threshold of TAP‐4 (*H*_50_ = 5.0 cm) was higher than that of DAP‐4 (*H*_50_ = 9.5 cm). The *H*_50_ values of TAP‐1, TAP‐2 and TAP‐3 were 7.5, 10.0, and 12.0 cm, respectively, and were lower than that of TAP‐4. This suggests that the presence of metal ions is beneficial for reducing the ignition threshold. In general, the flame sensitivity of TAPs is insensitivity.

**Table 1:** Sensitivity of TAPs, DAP‐4, RDX, HMX, and CL‐20.

Compd	*T*_d_ ^a)^ [°C]	*IS* ^b)^ [J]	*FS* ^c)^ [N]	*FLS* ^d)^ [cm]
TAP‐1	198	4	216	7.5
TAP‐2	208	8	324	10.0
TAP‐3	200	5	324	12.0
TAP‐4	203	5	240	5.0
DAP‐4	369	23	36	9.5
RDX^e^ ^)^	210	7.4	120	–
HMX^e^ ^)^	279	7.4	120	–
CL‐20^e^ ^)^	215	4.0	60	–

^a)^ Onset decomposition temperature;

^b)^ Impact sensitivity according to the BAM drop hammer (method 1 of 6);

^c)^ Friction sensitivity according to the BAM friction tester (method 1 of 6);

^d)^ Flame sensitivity;

^e)^ Ref. [32].

### 2.5 Influencing Factors on Mechanical Sensitivity

**Figure 6:**
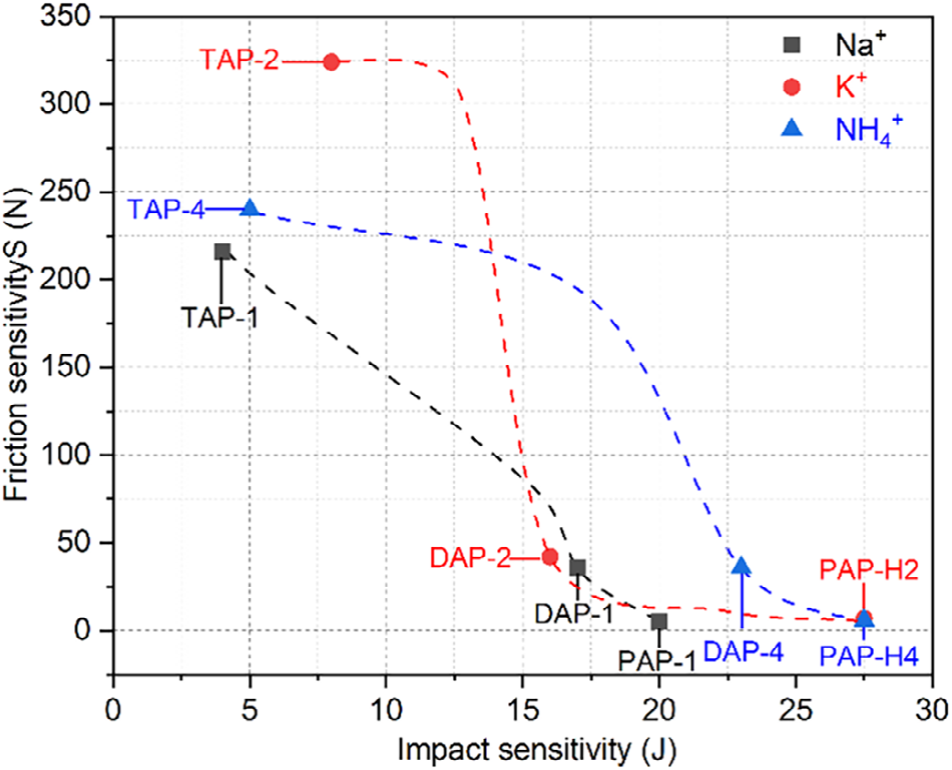
Trends on mechanical sensitivity changes of different series of PEMs ([C_5_H_14_N_2_][K(ClO_4_)_3_] for PAP‐H2, [C_6_H_14_N_2_][K(ClO_4_)_3_] for DAP‐2, [C_6_H_14_N_4_][K(ClO_4_)_3_] for TAP‐2, [C_5_H_14_N_2_][NH_4_(ClO_4_)_3_] for PAP‐H4, [C_6_H_14_N_2_][NH_4_(ClO_4_)_3_] for DAP‐4, [C_6_H_14_N_4_][NH_4_(ClO_4_)_3_] for TAP‐4).

Sensitivity is a key indicator for assessing the safety of compounds. Grasping the fundamental principles that influence sensitivity is essential for designing insensitive energetic materials. A comparison of the mechanical sensitivities between TAPs and DAPs revealed a notable phenomenon. As shown in **Figure** **6**, the impact sensitivity values of TAPs exhibited a significant downward trend. In contrast, the friction sensitivity value of TAPs demonstrated a significant upward trend.

**Figure 7:**
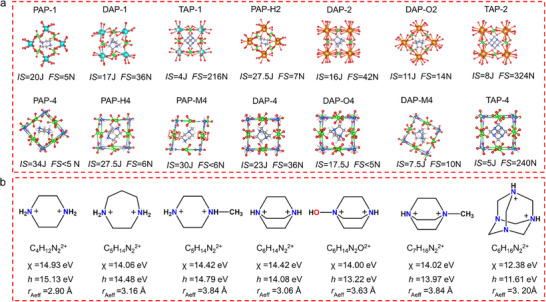
a) the frameworks and corresponding mechanical sensitivities of different PEMs (note: [C_6_H_14_N_2_O][K(ClO_4_)_3_] for DAP‐O2, [C_4_H_12_N_2_][NH_4_(ClO_4_)_3_] for PAP‐4, [C_5_H_14_N_2_][NH_4_(ClO_4_)_3_] for PAP‐M4, [C_6_H_14_N_2_O][NH_4_(ClO_4_)_3_] for DAP‐O4, [C_7_H_16_N_2_][NH_4_(ClO_4_)_3_] for DAP‐M4). b) The Mulliken electronegativity (χ), hardness (*h*) and effective radius (*r*_Aeff_) of different A‐site cations.

To investigate the factors influencing mechanism sensitivity, perchlorate‐based PEMs with B‐site is Na^+^, K^+^, and NH_4_^+^ were selected for comparison. As depicted in **Figure** **7**a, the impact sensitivity values decreased sequentially from PAP‐1 (20 J) to DAP‐1 (17 J) and then to TAP‐1 ([C_6_H_14_N_4_][NH_4_(ClO_4_)_3_], 4 J), while the friction sensitivity values exhibited the opposite trend (PAP‐1: 5 N; DAP‐1: 36 N; TAP‐1: 216 N). The similar trend was observed among PAP‐H2 (27.5 J, 7 N), DAP‐2 (16 J, 42 N), and TAP‐2 (8 J, 324 N), as well as among PAP‐H4 (27.5 J, 6 N), DAP‐4 (23 J, 36 N), and TAP‐4 (5 J, 240 N).

#### 2.5.1 Impact Sensitivity

A typical PEMs (ABX_3_) consists of a skeleton made from BX_3_^2−^, with organic amine cations incorporated within the framework. The BX_3_^2−^ framework of PEMs is stabilized by the attraction of A‐site cations. The strength of the entire framework depends on the binding strength between B‐site ions and X‐site ions, as well as the attraction of A‐site ions to BX_3_^2−^ framework. First, the distances from B‐site ion in a typical framework to the center of the adjacent perchlorate ions were assessed. Second, the framework was modeled as a parallelepiped and the volume of the framework was calculated based on the measured side lengths and angles. All the data are presented in Table S5 (Supporting Information). However, no significant correlation was observed between impact sensitivity and size of the framework. Hence, important related factors may not have been considered. To analyze the coulombic attraction of A‐site cations to the framework, the HSAB was introduced to quantify this attraction. Chemical potential (*μ*), electronegativity (*χ* = −*μ*) and chemical hardness (*h*) are commonly used to describe the strength of Lewis bases and Lewis acids.^[^ ^33^ ^]^

Various A‐site cations were calculated at the level of m062x/def2tzvp (Detailed information can be found in Supporting Information). Finally, *h* was seclected to characterize the hardness of these A‐site cations. For PAP‐H2, DAP‐2 DAP‐O2, and TAP‐2, the order of *h* values for their A‐site cations is as follows: C_5_H_14_N_2_^2+^ (homopiperazine‐1,4‐diium) > C_6_H_14_N_2_^2+^ > C_6_H_14_N_2_O^2+^ > C_6_H_14_N_4_^2+^. The above results are coincided with their impact sensitivity.

**Figure 8:**
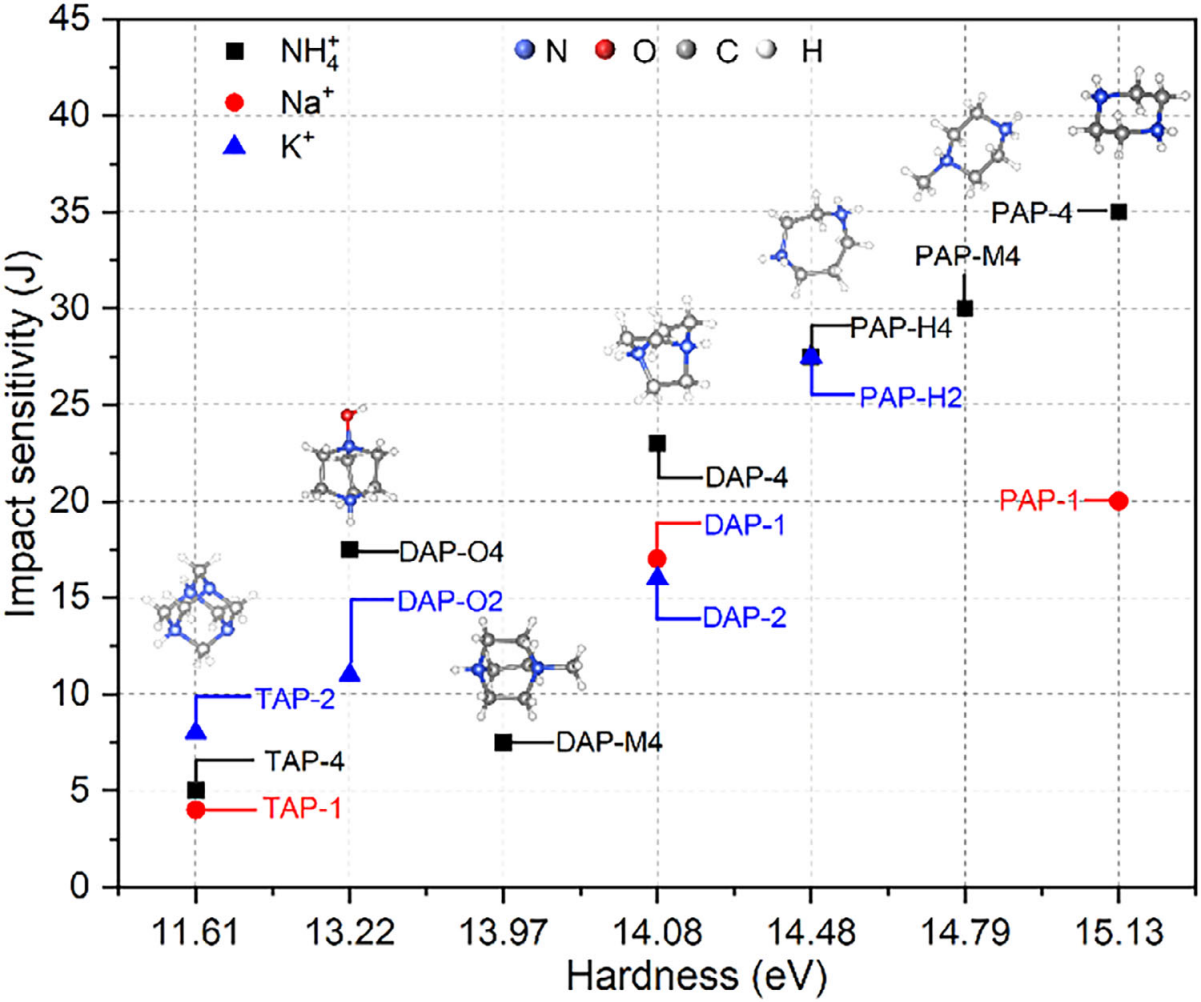
The hardness of different A‐site cations and impact sensitivity of corresponding PEMs.

As shown in **Figure** **8**, the order of *h* values for the seven A‐site cations was as follows: C_4_H_12_N_2_^2+^ > C_5_H_15_N_2_^2+^ (1‐methyl‐piperazine‐1,4‐diium) > C_5_H_14_N_2_^2+^ (homopiperazine‐1,4‐diium) > C_6_H_14_N_2_^2+^ > C_7_H_16_N_2_^2+^ > C_6_H_14_N_2_O^2+^ > C_6_H_14_N_4_^2+^. Correspondingly, their respective PEMs with NH_4_^+^ as the B‐site exhibited the same order of impact sensitivity, except for DAP‐O4 and DAP‐M4. This can be explained as follows. A comparison of their A‐site ion radii reveals that the radius of C_7_H_16_N_2_^2+^ is larger than that of C_6_H_14_N_2_O^2+^ (Figure 7b). Additionally, the distance from the B‐site ion to the perchlorate indicates that the framework of DAP‐M4 is larger than that of DAP‐O4 (detailed data in Table S6, Supporting Information). These results indicate that the size of the framework plays a dominant role in impact sensitivity of DAP‐O4 and DAP‐M4. In summary, the hardness of A‐site cations and the size of the framework are key factors influencing the impact sensitivity of PEMs. For PEMs with identical B‐site cations and X‐site anions, the hardness of the A‐site ion is the primary factor influencing impact sensitivity. A higher hardness of the A‐site cation corresponds to lower sensitivity to impact. If the volume of the A‐site cation is excessively large, it increases the distance between the B‐site cation and the X‐site anion, thereby weakening the attraction between them. Under the circumstances, impact sensitivity is influenced by both the volume and hardness of the A‐site ions.

#### 2.5.2 Friction Sensitivity

Interestingly, the friction sensitivity values increased sequentially from PAP‐1 to DAP‐1 and then to TAP‐1 in Figure 7a, Dabco, which has two more carbon atoms than Pz, resulted in four additional C─H bonds, while Tazcd contained two additional N atoms compared to Dabco.

First, the Hirshfeld surface analysis and fingerprint plots for the A‐site organic cations were performed to investigate their interactions with adjacent molecules using CrystalExplorer17.^[^ ^34^ ^]^ The colors of Hirshfeld surface were used to represent the proximity of close contacts (White: *d* equals to the van der Waals distance; Blue: *d* exceeds the van der Waals distance; Red: *d* is shorter than van der Waals distance).

**Figure 9:**
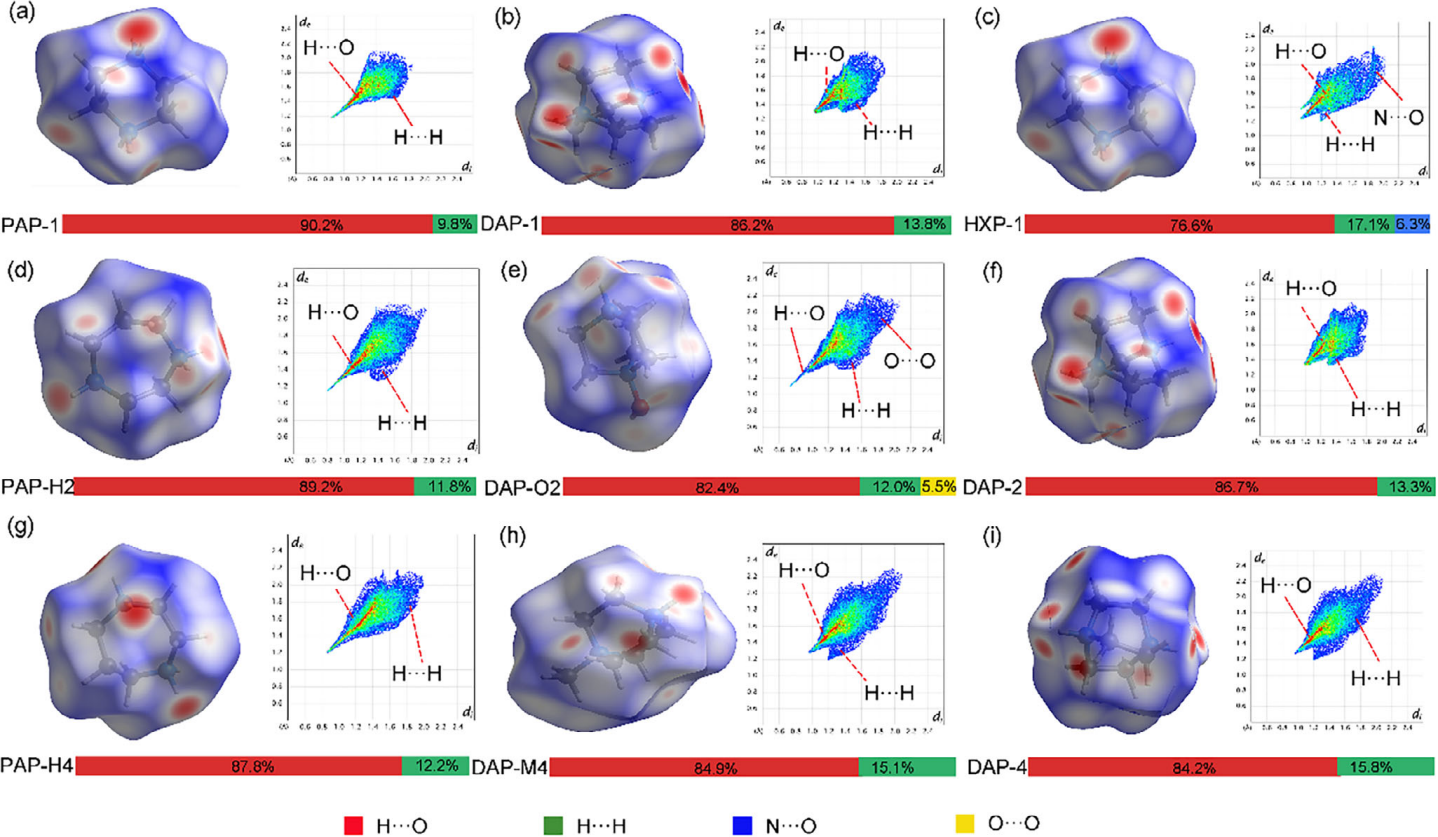
Hirshfeld surfaces mapped with *d*_norm_ and 2D fingerprint plots for A‐site anion: a) PAP‐1; b) DAP‐1; c) TAP‐1; d) PAP‐H2; e) DAP‐O2; f) DAP‐2; g) DAP‐O4; h) DAP‐M4 and i) TAP‐4.

As shown in **Figure** **9**, the Hirshfeld surfaces exhibited red color around C─H bond, indicating the presence of interactions between H atoms and adjacent O atoms. In addition to the conventional N─H···O hydrogen bonds, there were also a large number of C─H···O hydrogen bonds between A‐site organic cations and perchlorate anions. Furthermore, Van der Waals force were prevalent among A‐site cations in PAP‐1, DAP‐1, and TAP‐1. Additionally, N···O interactions (6.3%) were present in TAP‐1. Notably, C─H···O hydrogen bonds play a significant role in stabilizing crystal structures. C─H···O hydrogen bonds are widespread in biology.^[^ ^35^ ^]^ Although the energy of C─H···O hydrogen bonds is lower than that of conventional hydrogen bonds,^[^ ^35^, ^36^, ^37^ ^]^ they have been extensively utilized in designing hinge‐binding motifs of kinase inhibitors and other protein‐drug complexes.^[^ ^37^, ^38^ ^]^ In Figure 9, the environments surrounding the H atoms of the A‐site cations varied, leading to significant differences in their ability to attract negative charges.

**Figure 10:**
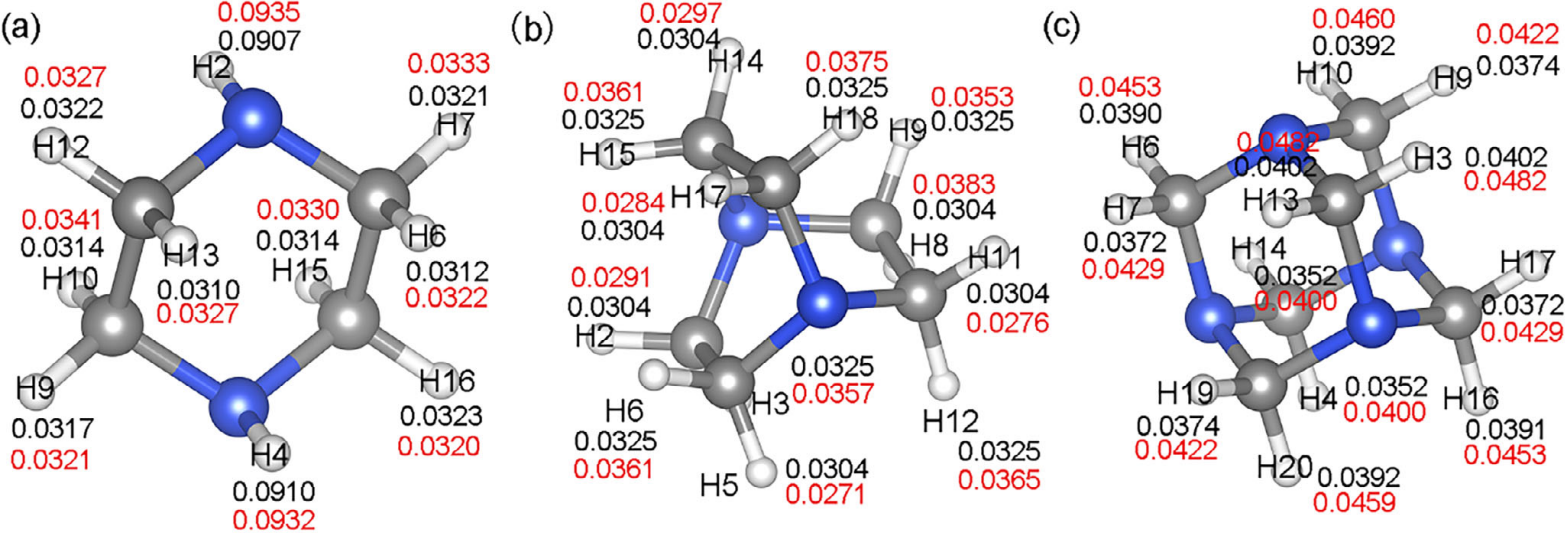
Hirshfeld charges (black) and VDD charges (red) of the H atoms in three types of organic amine molecules: a) Pz; b) Dabco; c) Tazcd.

The Hirshfeld charges and Voronoi Deformation Density (VDD) charges of each atom in small organic molecules (Pz, Dabco, and Tazcd) were calculated to verify differences in the charges of H atoms.^[^ ^39^, ^40^, ^41^ ^]^ As shown in **Figure** **10**, it was found that the Hirshfeld charge of H atoms in Pz and Dabco were nearly identical. However, the Hirshfeld charges of H atoms in Tazcd were significantly greater than those in PIP and Dabco. A larger Hirshfeld charge on H atoms indicates that its adjacent C atom has a stronger ability to attract electrons. Greater exposure of the H proton correlates with stronger electrostatic attraction to the O atom. Tables S7–S9 (Supporting Information) indicate that the Hirshfeld charges of C atoms in Pz and Dabco are negative, while those in Tazcd are positive. This difference suggests that the electron cloud of C atom in Tazcd is closer to N atoms, enhancing the attraction of C atoms to the electrons of H atoms. Consequently, the H atoms in Tazcd readily form stronger the stronger C─H···O type hydrogen bond with the surrounding O atoms.

A significant number of C─H···O hydrogen bonds existed between the A‐site cations and the cage frames (Figures S14–S22, Supporting Information). Compared to the cage frames in PAP‐1, DAP‐1, and TAP‐1, the number of C─H···O hydrogen bonds in PAP‐1 was significantly lower than in DAP‐1, and the number of C─H···O hydrogen bonds in TAP‐1 had an equal number of such bonds as DAP‐1. However, the bond energy of some C─H·· O hydrogen bonds were significantly less than 1.00 kcal mol^−1^. These weak bonds contributed minimally to the crystal stability and could therefore be ignored. C─H···O hydrogen bonds with the maximum bond energy were oriented along various C─H directions in the cage frames of different PEMs. The results are summarized in Tables S10–S21 (Supporting Information).

These hydrogen bond energy results indicated that the average bond energy of C─H···O hydrogen bond in the cage frame of PAP‐1 was 1.54 kcal mol^−1^, lower than that of DAP‐1 (1.77 kcal mol^−1^). Additionally, the number of C─H···O type hydrogen bonds with bond energies greater than 1.00 kcal mol^−1^ in the PAP‐1 and DAP‐1 cage frames was 14 and 15, respectively. The average bond energy of C─H···O hydrogen bonds in TAP‐1 cage frame was 1.78 kcal mol^−1^, close to that of C─H···O hydrogen bond in DAP‐1 cage frame. In TAP‐1, all C─H···O hydrogen bonds possesses bonding energies greater than 1.00 kcal mol^−1^, and the number of these bonds exceeded that in DAP‐1 (Table S22, Supporting Information).

Additionally, the distribution of C─H bonds in A‐site cations of PAP‐1, DAP‐1, and TAP‐1 was investigated. Notably, the spatial distribution of C─H bonds in DAP‐1 was more spherical than that in PAP‐1. Furthermore, the spatial distribution of C─H bonds in TAP‐1 was more uniform and closer to a sphere than that in DAP‐1. When subjected to frictional stimuli, the C─H bonds in the A‐site cation contract in response to vibration, which was beneficial for absorbing this stimulus. Consequently, the order of sustained release ability of three PEMs to frictional stimuli was as follows: TAP‐1 > DAP‐1 > PAP‐1. Moreover, the C─H···O hydrogen bond energy in TAP‐1 was exceeds 1.00 kcal mol^−1^. Thus, TAP‐1 exhibited the excellent friction sensitivity.

**Figure 11:**
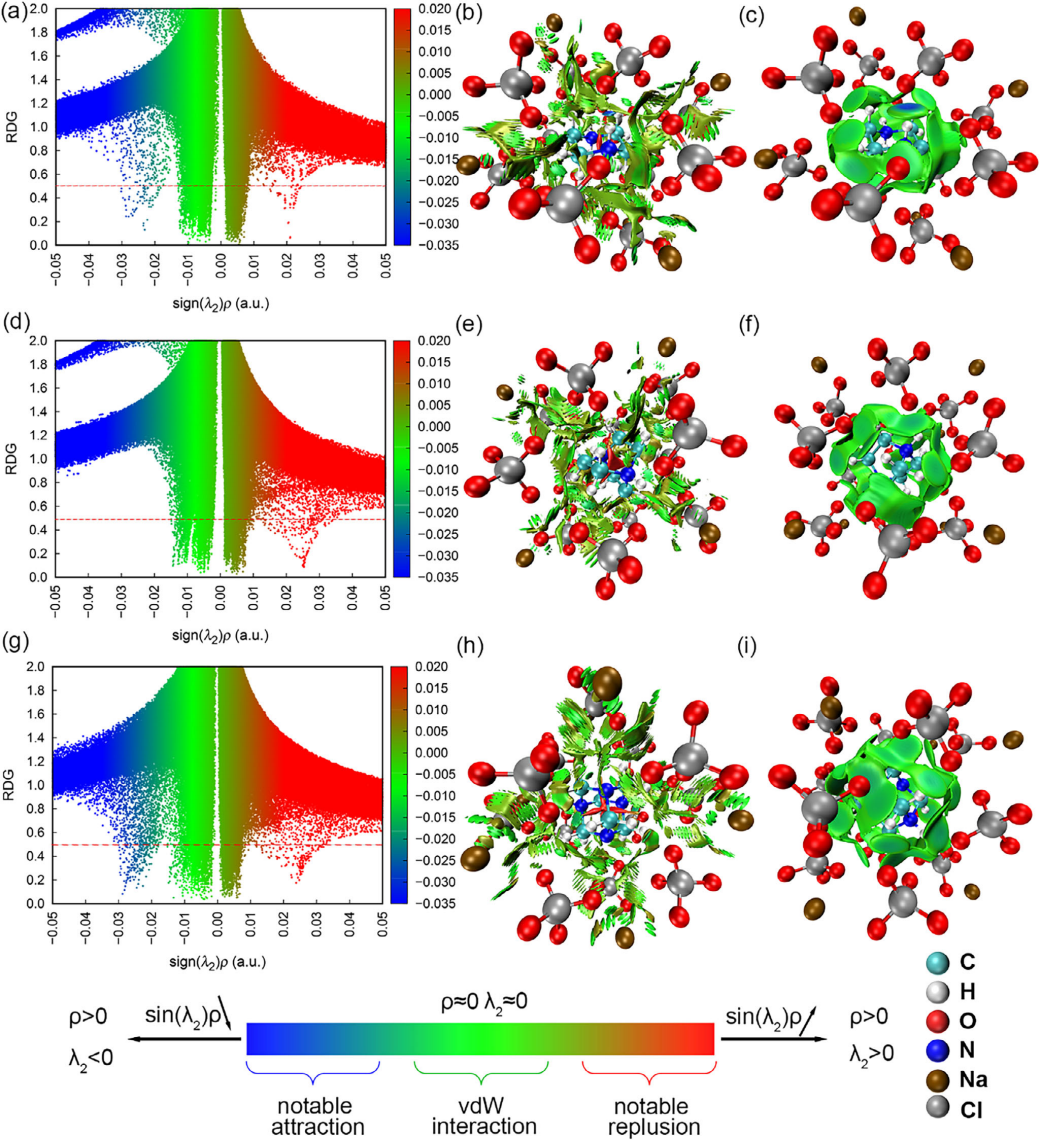
IGMH analysis of PAP‐1, DAP‐1 and TAP‐1. Plots of RDG versus sin(*λ*_2_)*ρ*: a) PAP‐1, d) DAP‐1 and g) TAP‐1; Gradient isosurfaces (*s* ^pro^ = 0.25) of b) PAP‐1, e) DAP‐1 and h) TAP‐1; sign*(λ_2_)ρ* colored isosurfaces of δ_g_^inter^ = 0.005 a.u. of c) PAP‐1, f) DAP‐1, and i) TAP‐1.

To further verify and analyze the influence of weak interaction on sensitivity, PAP‐1, DAP‐1, and TAP‐1 were selected as typical samples for visual analysis. Multiwfn software was used to analyze the Revealing Noncovalent Interactions (RDG) of the cage unit, providing an intuitive representation of interaction types and weak interaction regions.^[^ ^42^ ^]^ Concurrently, IGMH (independent gradient model based on Hirshfeld partition) was employed to illustrate the weak interaction region between the A‐site cation fragment within the cage frame unit and the surrounding B_8_X_12_^4‐^ anion fragment.^[^ ^43^ ^]^ The results are presented in **Figure** **11**.

As shown in Figure 11a,d,g, the leftmost spike represents the strongest hydrogen bonding between the A‐site cation and the surrounding framework among the four PEMs. The corresponding contour filling diagram allows for the clear identification of the position with the strongest hydrogen bonding. In PAP‐1, the *ρ*(r) of the blue spikes on the left side of PAP‐1 was more than 0.020, representing N─H···O hydrogen bonds. The *ρ*(r) value of the leftmost spike in TAP‐1 was 0.029. The *ρ*(r) values of the leftmost spikes in PAP‐1 and TAP‐1 were both greater than that in DAP‐1, indicating the differences in hydrogen bond strength between the A‐site cations and the surrounding framework among the four PEMs.

Comparing the widths of spikes with *ρ*(r) within 0.000–0.020 among the scatter plots of three PEMs among the scatter plots of the three PEMs revealed that the spike for TAP‐1 occupied a wider width, indicating a broader range of weak hydrogen bonds. In the case of the three PEMs, the red spike on the far right represented the repulsive force within the annular or cage structure, observable in the isosurface map at an isovalue of 0.5. The differences in *ρ*(r) values of the rightmost spikes among the three PEMs suggest that the piperazine cation experiences the least repulsive force. The dispersion of the rightmost spike indicates that the distribution of strong repulsive forces among the A‐site cations follows the order: TAP‐1 > DAP‐1 > PAP‐1. The *ρ*(r) values of the three peaks on the right side of the PEM indicate that the repulsive force is greatest in TAP‐1, suggesting that the interactions are relatively stronger.

The IGMH isosurface analysis of the A‐site cations and anion frames of the three PEMs reveals numerous weak interactions between the A‐site cations and anion frames, with the position of the strongest mutual attraction is clearly displayed (Figure 10c,f,i). The distribution of weak interaction around the A‐site cations was more uniform in TAP‐1 and DAP‐1 compared to PAP‐1. The order of spatial distribution sphericity of weak interactions around the A‐site cations was TAP‐1 > DAP‐1 > PAP‐1, consistent with the strength and spatial distribution of weak hydrogen bonds. The same situation existed in the other series of PEMs. The corresponding RDG scatter plots and contour surface coloring maps are presented in Figures S26 and S27 (Supporting Information).

### 2.6 Detonation Performance

**Figure 12:**
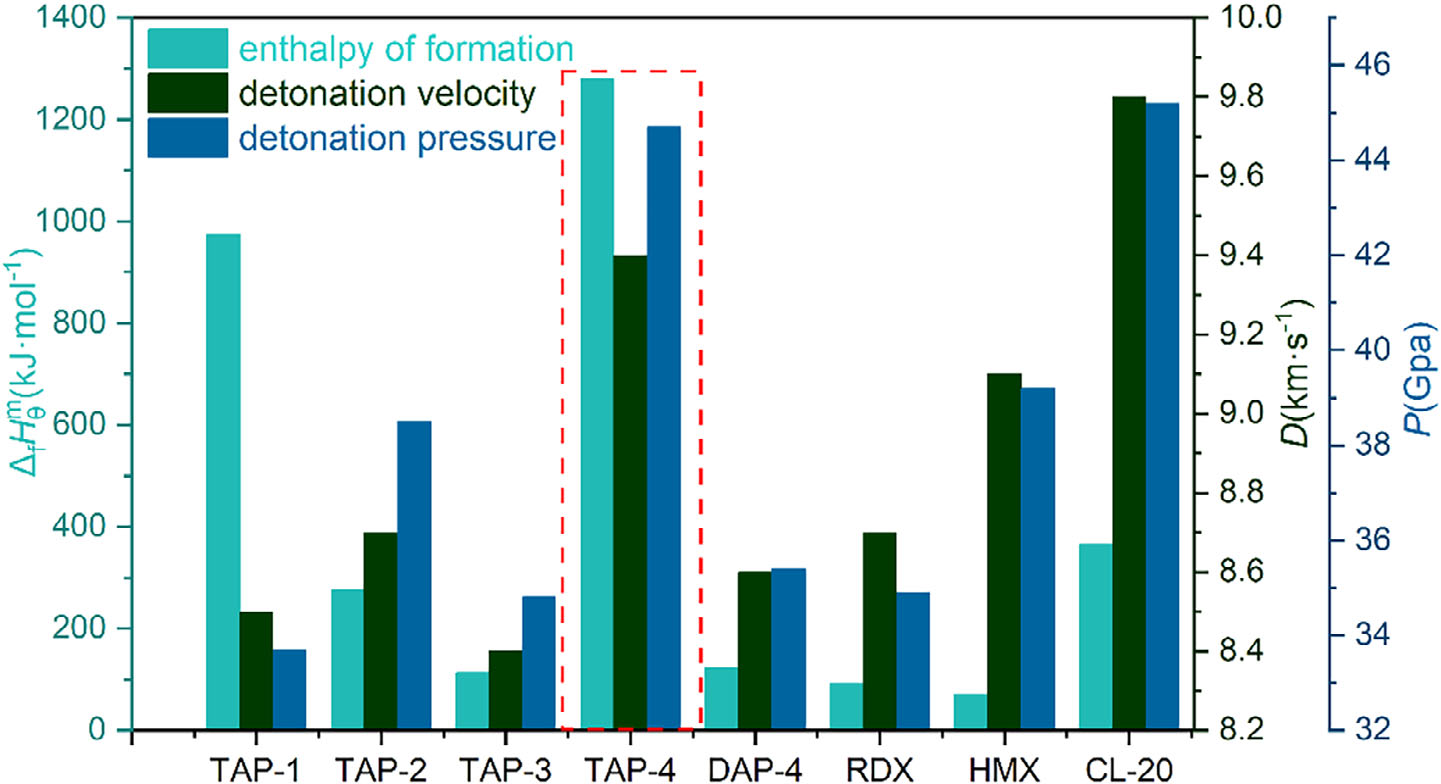
Comparison of enthalpy of formation and detonation performance.

High energy and insensitivity are key objectives for the development of energetic materials. A‐site cation is crucial for enhancing the detonation performance of PEMs. At the beginning of the design, A‐site cations with high enthalpy of formation ($\Delta_{f}H_{\theta }^{m}$) and high nitrogen content have always been preferred. The detonation performance of new PEMs was calculated through the K‐J equation and EXPLO5 (more calculations and discussion were provided in Supporting Information). As shown in **Figure** **12**, the enthalpy of formation ($\Delta_{f}H_{\theta }^{m}$) value of TAP‐1 and TAP‐4 were 974.3 and 1279.6 kJ mol^−1^, respectively. the $\Delta_{f}H_{\theta }^{m}$ values of other TAPs were 275.2 kJ mol^−1^ for TAP‐2, and 113.2 kJ mol^−1^ for TAP‐3. As expected, the $\Delta_{f}H_{\theta }^{m}$ of TAP‐4 was much higher than that of DAP‐4 (112.8 kJ mol^−1^). In addition to $\Delta_{f}H_{\theta }^{m}$, the crystal density is also a vital factor for determining detonation performance. Except for TAP‐1, the crystal density of each TAP was greater than that of the corresponding DAP. Furthermore, the results of the oxygen balance for TAPs indicate that, compared to DAPs with identical B‐site cations, the increase in nitrogen atom content contributes to improve oxygen balance, as evidenced by TAP‐4 (‐26.17%) and DAP‐4 (‐28.17%). The presence of nitrogen atoms in the form of N_2_ in the detonation equation allows for an increase in gas product content during detonation without consuming oxygen, which enhances detonation performance.

Detonation velocity (*D*) and detonation pressure (*P*) are two most important aspects of detonation performance. The calculation results indicated that TAPs had excellent detonation performance. The detonation velocities of TAPs were 8.5 km s^−1^ for TAP‐1, 8.7 km s^−1^ for TAP‐2, 8.4 km s^−1^ for TAP‐3, and 9.4 km s^−1^ for TAP‐4. Among them, the detonation velocity of TAP‐4 was particularly prominent, which was greater than that of RDX (9.0 km s^−1^) and close to that of CL‐20 (9.7 km s^−1^). Significantly, the increase in nitrogen content of A‐site cations of TAPs caused obvious changes in explosion pressure. The detonation pressures of TAPs were within the range of 33.7 to 44.7 GPa. The detonation pressure of TAP‐4 (44.7 GPa) was higher than that of DAP‐4 (35.4 GPa) and RDX (34.9 GPa). The detonation pressure of TAP‐1 (33.7 GPa) was the smallest among four TAPs, and the rest are 38.5 GPa for TAP‐2, and 34.8 GPa for TAP‐3. Among the reported PEMs, DAP‐4 has the highest energy. As a metal‐free explosive, TAP‐4 has the excellent detonation performance and combustion heat that is significantly exceeding DAP‐4. These results show that introducing Tazcd as the A‐site cation leads to a significant improvement in detonation performance. The result of lead plate perforation experiment also shows that Tazcd, as an A‐site cation, contributes to the increase in detonation performance (more information can be found in Support Information). Considering the characteristics of Tazcd, the selection of A‐site cations can be directed toward high nitrogen content.

## 3 Conclusion

In summary, the urotropine‐based PEMs with distinct structural features were successfully prepared by replacing the A‐site cation. Different from the reported cubic PEMs, TAPs undergo lattice compression along *c*‐axis. As designed, the crystal density of TAPs increased compared to the corresponding DAPs, except for TAP‐1. The onset decomposition temperature of all TAPs began at 198–208 °C, which was close to that of RDX. And the thermal decomposition of TAPs occurred simultaneously with melting. Notably, the friction sensitivity of TAPs was very insensitive (216–324 N). However, the impact sensitivity of TAPs was not satisfactory (4–8 J). The hardness and size of A‐site cations significantly influenced the impact sensitivity of PEMs. Moreover, The C─H···O hydrogen bond played a critical in reducing the friction sensitivity of PEMs. Based on the calculated results, TAPs exhibited excellent detonation performance (8.4–9.4 km s^−1^, 33.7–44.7 GPa). Among the reported PEMs, TAP‐4 exhibited the highest detonation performance (9.4 km s^−1^ and 44.7 GPa). The exceptional performances is attributed to the introduction of H_2_tazcd^2+^ as A‐site cation. Considering the excellent detonation performance and insensitivity of TAPs, the selection of A‐site cations for PEMs can focus on increasing nitrogen content direction. Meanwhile, Simultaneously, overcoming strict size restrictions presents the greatest challenge in the design of PEMs.

## 4 Experimental Section

### Chemicals and Materials

All raw materials were reagent grade and used without further purification. 1,3,5,7‐tetraazatricy‐clo[3.3.1]decane (99%) and perchloric acid (70%) were commercially available from Meryer Co., Ltd (China). Sodium chloride, potassium chloride, ammonium chloride and ethanol were purchased from Sinopharm Chemical Reagent Co., Ltd (China). Rubidium chloride was acquired from Tianjin HEOWNS Technology Co., Ltd. The deionized water was from the lab.

### Synthesis of [C_6_H_14_N_4_][M(ClO_4_)_3_] (TAP‐1, 2, 3, 4; M = Na^+^, K^+^, Rb^+^, NH_4_^+^)

(C_6_H_14_N_4_)[Na(ClO_4_)_3_], as a typical example, was synthesized by following steps. First, Tazcd (1.40 g, 10 mmol) and NaCl (0.58 g, 10 mmol) were dissolved in 15.0 mL of deionized water at room temperature under fast stirring, forming clear solution in a silanized glass vial. HClO_4_ (4.30 g, 30 mmol) was added dropwise into the previous solution. After the addition, the mixture was stirred at room temperature for 25 min. The white precipitate was obtained by filtration and washed three times with ethanol, then dried to give TAP‐1 (yield 43.86%). [C_6_H_14_N_4_][NH_4_(ClO_4_)_3_], (C_6_H_14_N_4_)[K(ClO_4_)_3_] and (C_6_H_14_N_4_)[Rb(ClO_4_)_3_] were synthesized with similar procedure as (C_6_H_14_N_4_)[Na(ClO_4_)_3_], except that 0.54 g (10 mmol) of NH_4_Cl, 0.74 g (10 mmol) of KCl and 1.20 g (10 mmol) of RbCl were used to substitute for NaCl, in yield of 90.67%, 99.66%, and 62.94%, respectively.

### Measurements

Powder X‐ray diffraction (XRD) pattern was acquired on a Bruker D8‐Advance diffractometer using a Cu source scanned from 5°–80° with a step size of 0.02°. Differential scanning calorimetry (DSC) analysis and thermal gravimetric analyzer (TG) were implemented on a TGA/DSC3+ to determine the thermal decomposition of the sample at the rate of 10 K min^−1^ under nitrogen atmosphere. After increasing the quantity of the sample, the thermal decomposition analysis was examined by DSC (NETZSCH 204), and the other testing conditions were same with the above TG‐DSC. The combustion heat of the sample was measured by oxygen bomb calorimeter (IKA‐C2000, Germany) under oxygen atmosphere at the pressure of 1.23 MPa. The reaction and crystallization processes of TAP‐2 were tracked through in situ Raman (ReactRaman 802L, METTLER TOLEDO, Switzerland).

### Sensitivity Tests

Impact sensitivity was measured according to the BAM drop hammer (method 1 of 6). Friction sensitivity was measured according to the BAM friction test (method 1 of 6). The electrostatic sensitivity was measured using electrostatic spark sensitivity tester (JGY‐50III) according to GJB 891.27‐2006. The charging capacitor was 500 pF, and the gap between the electrodes was 0.12 mm. ≈20 mg of powder were loaded. The flame sensitivity was tested according to GJB.5891.25‐2006. ≈20 mg of the sample was compacted to a copper cap under a pressure of 20 MPa and was ignited by a black powder pellet.

### Lead Plate Perforation Test

In this test, a 500 mg sample was used as the secondary charge to detonate a 5 mm thick lead plate. The detonation performance was evaluated by measuring the perforation diameter of the lead plate. A 220 mg RDX was applied as the transition charge on the upper layer of the secondary charge under soft pressure. Finally, 100 mg of nickel hydrazine azide (NHA) was loaded as the primary explosive into an 8# industrial detonator and ignited using a standard electrical igniter. The primary charge and secondary explosive were compressed under static pressures of 30 and 50 MPa, respectively. Each sample undergone two experiments, where the maximum and minimum pore sizes are measured independently, and the average of these values is used to represent the pore size. The maximum value from the two experiments is selected to represent the detonation performance under the given condition.

[CCDC 2254489, CCDC 2321804, and CCDC 2226281 contain the supplementary crystallographic data for this paper. These data can be obtained free of charge from the Cambridge Crystallographic Data Centre via www.ccdc.cam.ac.uk/data_request/cif.]

## Conflict of Interest

The authors declare no conflict of interest.

## Supporting information



Supporting Information

Supporting Information

Supporting Information

Supporting Information

Supporting Information

Supporting Information

## Data Availability

The data that support the findings of this study are available in the supplementary material of this article.
