# Supplementary material for: Tetrahedral Nitrogen Atoms Arrangement in A‐Site Cations: A New Approach for Regulating Sensitivity and Energy of Perovskite Energetic Materials
Source: Adv Sci (Weinh). 2025 Mar 24;12(19):2415680. doi: 10.1002/advs.202415680 (PMC12097078; doi:10.1002/advs.202415680)
Supplement: Supplementary file 1 — Supporting Information [file ADVS-12-2415680-s006.docx]

**Tetrahedral Nitrogen Atoms Arrangement in A-site Cations: A New Approach for Regulating Sensitivity and Energy of Perovskite Energetic Materials**

*Shiyong Chen, Yuan Gao, Cheng Dong, Lixiao Shen, Yinning Zeng, Peng Bao, Yan Li, Zhenxin Yi, Houhe Chen, Shunguan Zhu and Lin Zhang**

School of Chemistry and Chemical Engineering, Nanjing University of Science and Technology, Nanjing, 210094, China
E-mail: zhangl@njust.edu.cn

**Contents**

- Annotation Table
- Calculation of tolerance factor
- Crystal data and structure refinement
- Calculation method of TAP-3
- *In-situ* reaction progress with PATs
- Hygroscopicity
- Thermal Stability Analysis
- Calculation of ionic hardness
- Hydrogen bonds statistics
- Calculation of detonation performance

**Annotation Table**

To facilitate reading, a separate annotation table was provided for the numerous abbreviations used in the manuscript.

Table 1 the abbreviations and corresponding molecular formulas of PEMs in the manuscript.

| Abbreviation | Formula |
| --- | --- |
| PAP-1 | [C_4_H_12_N_2_][Na(ClO_4_)_3_] |
| DAP-1 | [C_6_H_14_N_2_][Na(ClO_4_)_3_] |
| TAP-1 | [C_6_H_14_N_4_][Na(ClO_4_)_3_] |
| PAP-H2 | [C_5_H_14_N_2_][K(ClO_4_)_3_] |
| DAP-2 | [C_6_H_14_N_2_][K(ClO_4_)_3_] |
| DAP-O2 | [C_6_H_14_N_2_O][K(ClO_4_)_3_] |
| TAP-2 | [C_6_H_14_N_4_][K(ClO_4_)_3_] |
| PAP-4 | [C_4_H_12_N_2_][NH_4_(ClO_4_)_3_] |
| PAP-H4 | [C_5_H_14_N_2_][NH_4_(ClO_4_)_3_] |
| PAP-M4 | [C_5_H_14_N_2_][NH_4_(ClO_4_)_3_] |
| DAP-4 | [C_6_H_14_N_2_][NH_4_(ClO_4_)_3_] |
| DAP-O4 | [C_6_H_14_N_2_O][NH_4_(ClO_4_)_3_] |
| DAP-M4 | [C_7_H_16_N_2_][NH_4_(ClO_4_)_3_] |
| TAP-4 | [C_6_H_14_N_4_][NH_4_(ClO_4_)_3_] |

For the above compound abbreviations: the first letter denotes the first letter of the A-site cation name; the middle letter A indicates alkaline cations; the third letter P denotes the first letter of perchlorate ions. In PAP-M and DAP-M, M represents the methyl group (Me) attached to the N atom. In DAP-O, O indicates the hydroxyl group. In PAP-H, H represents the excess methylene group based on piperazine.

Table 2 the abbreviations and formula of organic amine and organic amine cations

| Abbreviation | Name | Formula |
| --- | --- | --- |
| Pz | piperazine | C_4_H_10_N_2_ |
| H_2_pz^2+^ | piperazine-1,4-diium | C_4_H_12_N_2_^2+^ |
| H_2_hpz^2+^ | homopiperazine-1,4-diium | C_5_H_14_N_2_^2+^ |
| H_2_mpz^2+^ | 1-methyl-piperazine-1,4-diium | C_5_H_14_N_2_^2+^ |
| Dabco | 1,4-diazabicyclo[2.2.2]octane | C_6_H_12_N_2_ |
| H_2_dabco^2+^ | 1,4-diazabicyclo[2.2.2]octane-1,4-diium | C_6_H_14_N_2_^2+^ |
| H_2_dabco-O^2+^ | 1-hydroxy-1,4-diazabicyclo[2.2.2]octane-1,4-diium | C_6_H_14_N_2_O ^2+^ |
| H_2_mdabco^2+^ | 1-methyl-1,4-diazabicyclo-[2.2.2]octane-1,4-diium | C_7_H_16_N_2_^2+^ |
| Tazcd | 1,3,5,7-tetraazatricy-clo[3.3.1]decane | C_6_H_12_N_4_ |
| H_2_tazcd^2+^ | 1,3,5,7-tetraazatricy-clo[3.3.1]decane-1,3-diium | C_6_H_14_N_4_^2+^ |

**Calculation of tolerance factor**

In contrast to inorganic perovskites, organic-inorganic hybrid perovskites incorporate organic cations or organic acid anions. The size of organic ions is defined by the equivalent radius (*r*_Aeff_), assuming spherical or columnar shapes. The specific calculation formula is provided in Equation S1.

$r_{\text{Aeff}}=r_{\text{mass}}+r_{\text{ion}}$ (S1)

Here, *r*_mass_ denotes the maximum distance from the ion`s center of mass to surrounding atoms (excluding hydrogen atoms), while *r*_ion_ represents the radius of the farthest from the center of mass.

The centroid coordinates of the A-site cation were calculated as the mass-weighted average of the coordinates of all atoms (excluding hydrogen atoms). Subsequently, the equivalent ionic radius of the A-site cation was then determined using the centroid coordinates, the coordinates of the farthest atom, and the ionic radius of that atom. The result is listed in **Table S3**.

Table S3 Calculation results of equivalent ionic radii for different A-site cations

| A-site cations |  |  |  |  |
| --- | --- | --- | --- | --- |
| *r*_mass_ (Å) | 1.60 | 1.74 | 2.28 | 2.46 |
| *r*_ion_ (Å) | 1.46 | 1.46 | 1.35 | 1.38 |
| *r*_Aeff_ (Å) | 3.06 | 3.20 | 3.63 | 3.84 |

The tolerance factor proposed by Goldschmidt was used to predict the structural stability of hybrid perovskites by modeling the organic ions as either spheres or cylinders. The calculation formula is given in Equation S2^[1-2]^:

$t=\left(R_{\text{Aeff}}+R_{\text{Xeff}}\right)/\left(R_{\text{B}}+0.5h_{\text{Xeff}}\right)$ (S2)

*R*_Aeff_ and *R*_Xeff_ represent the equivalent radii of A-site and X-site ions, respectively, while *h*_Xeff_ denotes equivalent height of X-site ions. Based on the tolerance factor calculation (**Table S4**), it is feasible to utilize Urotropine as the A-site cation.

Table S4 Calculation results of tolerance factor for perovskite type energetic materials

| Compd | Molecular formula | *t* |
| --- | --- | --- |
| DAP-1 | [C_6_H_14_N_2_][Na(ClO_4_)_3_] | 1.086 |
| DAP-2 | [C_6_H_14_N_2_][K(ClO_4_)_3_] | 0.934 |
| DAP-3 | [C_6_H_14_N_2_][Rb(ClO_4_)_3_] | 0.917 |
| DAP-4 | [C_6_H_14_N_2_][NH_4_(ClO_4_)_3_] | 0.980 |
| TAP-1 | [C_6_H_14_N_4_][Na(ClO_4_)_3_] | 1.117 |
| TAP-2 | [C_6_H_14_N_4_][K(ClO_4_)_3_] | 0.956 |
| TAP-3 | [C_6_H_14_N_4_][Rb(ClO_4_)_3_] | 0.939 |
| TAP-4 | [C_6_H_14_N_4_][ NH_4_(ClO_4_)_3_] | 0.997 |
| DAP-M4 | [C_6_H_16_N_2_][ NH_4_(ClO_4_)_3_] | 1.103 |

**Crystal data and structure refinement**

Due to the hydrolysis of Tazcd under acidic conditions, the single crystals of these compounds can’t be obtained by evaporating the reaction solution at room temperature. In order to overcome the difficulty, the diluted reaction solution was placed under low temperature conditions and single crystals were produced through slow evaporation except for TAP-3. The crystal structure of TAPs was determined by the single crystal diffraction, and analyzed by the Olex2 program. The details on crystallographic data can be found in **Table S5**.

Table S5. Crystallographic data and structural reﬁnements for TAP-1, TAP-2 and TAP-4.

| CCDC | 2226285 | 2321804 | 2226281 |
| --- | --- | --- | --- |
| Compd | TAP-1 | TAP-2 | TAP-4 |
| formula | C_6_H_14_N_4_NaCl_3_O_12_ | C_6_H_14_N_4_KCl_3_O_12_ | C_6_H_18_N_4_Cl_3_O_12_ |
| Formula weight | 463.55 | 479.66 | 457.59 |
| Temperature/K | 100.00(3) | 293(2) | 296.15(10) |
| crystal system | tetragonal | tetragonal | tetragonal |
| space group | I4_1_/a | I4_1_/a | I4_1_/a |
| a (Å) | 14.5909(3) | 14.5088(3) | 14.5863(3) |
| b (Å) | 14.5909(3) | 14.5088(3) | 14.5863(3) |
| c (Å) | 29.0818(8) | 28.9637(13) | 29.1042(8) |
| α (deg) | 90 | 90 | 90 |
| β (deg) | 90 | 90 | 90 |
| γ (deg) | 90 | 90 | 90 |
| V (Å^3^) | 6194.4(3) | 6097.0(4) | 6192.2(3) |
| Z | 16 | 16 | 16 |
| Dc (g cm^−3^) | 1.989 | 2.090 | 1.963 |
| no. reﬂections collected | 17117 | 2698 | 3173 |
| no. of unique reﬂections | 17117 | 2698 | 3173 |
| Rint | 0.0520 | 0.0785 | 0.0314 |
| R_1_ [I >2σ(I)]^a)^ | 0.0520 | 0.0785 | 0.0533 |
| wR_2_ [I >2σ(I)]^b)^ | 0.1499 | 0.2336 | 0.1174 |
| R_1_ (all data) | 0.0597 | 0.0804 | 0.0561 |
| wR_2_ (all data) | 0.1578 | 0.2363 | 0.1189 |
| Goodness-of-fit on F^2^ | 1.139 | 1.218 | 1.197 |

^a)^ R_1_ = ∑||F_0_| − |F_c_||/∑|F_0_|; ^b)^ wR_2_ ={∑w[(F_0_)^2^ − (F_c_)^2^]2/∑w[(F_0_)^2^]^2^}^1/2^.

CCDC 2226285, 2321804 and 2226281 contain the supplementary crystallographic data for this paper. These data can be obtained free of charge from the Cambridge Crystallographic Data Centre via [www.ccdc.cam.ac.uk/data_request/cif](http://www.ccdc.cam.ac.uk/data_request/cif).


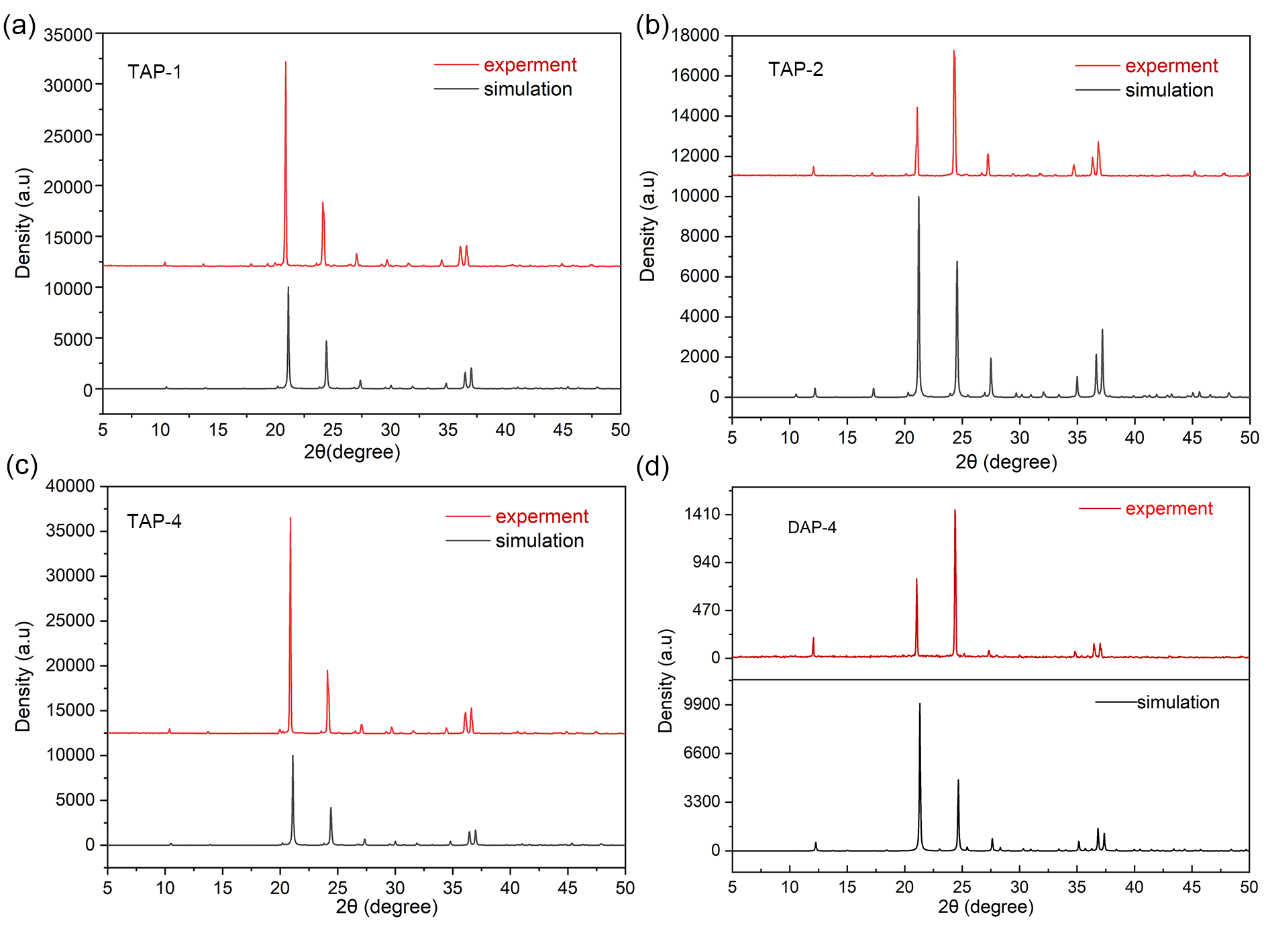
Figure S1. the comparison of experimental and simulated PXRD patterns: (a) TAP-1, (b) TAP-2, (c) TAP-4 and (d) DAP-4.

**Calculation method of TAP-3**

Single crystal of TAP-3 was not able to be obtained for structural analysis. However, it was found from **Figure S3a** that TAP-3 has the similar PXRD patterns with other TAPs (TAP-1, TAP-2 and TAP-4). In addition, the crystal structures of DAPs are similar, thus sharing similar PXRD patterns (**Figure S2**). It is believed that TAP-3 and TAP-2 should have similar crystal structures. The crystal cell model was constructed for structural optimization (**Figure S3b**). After optimization, the simulated PXRD was generated by using the optimized structure, and compared with the experimental pattern. The results indicate that the simulated PXRD is consistent with the experimental PXRD (Figure S2c). The calculation model and steps are as follows:

The calculations were performed based on the density functional theory (DFT) in periodic boundary conditions, as implemented in the CP2K/Quickstep package.^[3]^ The hybrid Gaussian and plane wave (GPW) method was used to treat the ion-electron interactions.^[4]^ The Gaussian functions consisting of a double-ζ plus polarization (DZVP) basis set in combination of norm conserving Goedecker-Teter-Hutter (GTH) pseudo-potentials was used to describe all elements.^[5-7]^ The crystals were optimized with the exchange-correlation functional of Perdew-Burke-Ernzerhof (PBE) under generalized gradient approximation (GGA).^[8]^ The energy cutoff was set to be 1000 Ry and the energy convergent standard was 5×10^-7^ Hartree. The structures and lattice parameters were fully relaxed until the maximum force on each atom was less than 2.0◊10^-4^ Hartree/Bohr. The vdW correction is considered with the Grimme approach (DFT-D3BJ).^[9-10]^ The energy convergent standard was 110^-8^ Hartree.


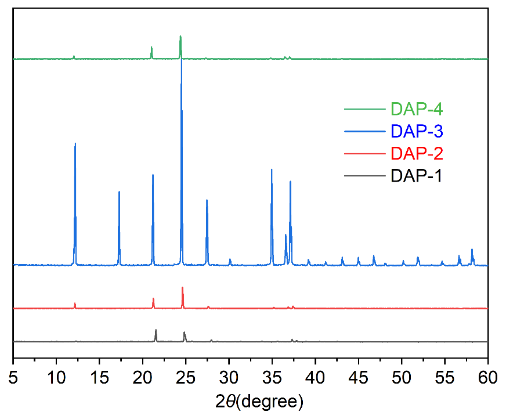


Figure S2. PXRD patterns of DAPs.


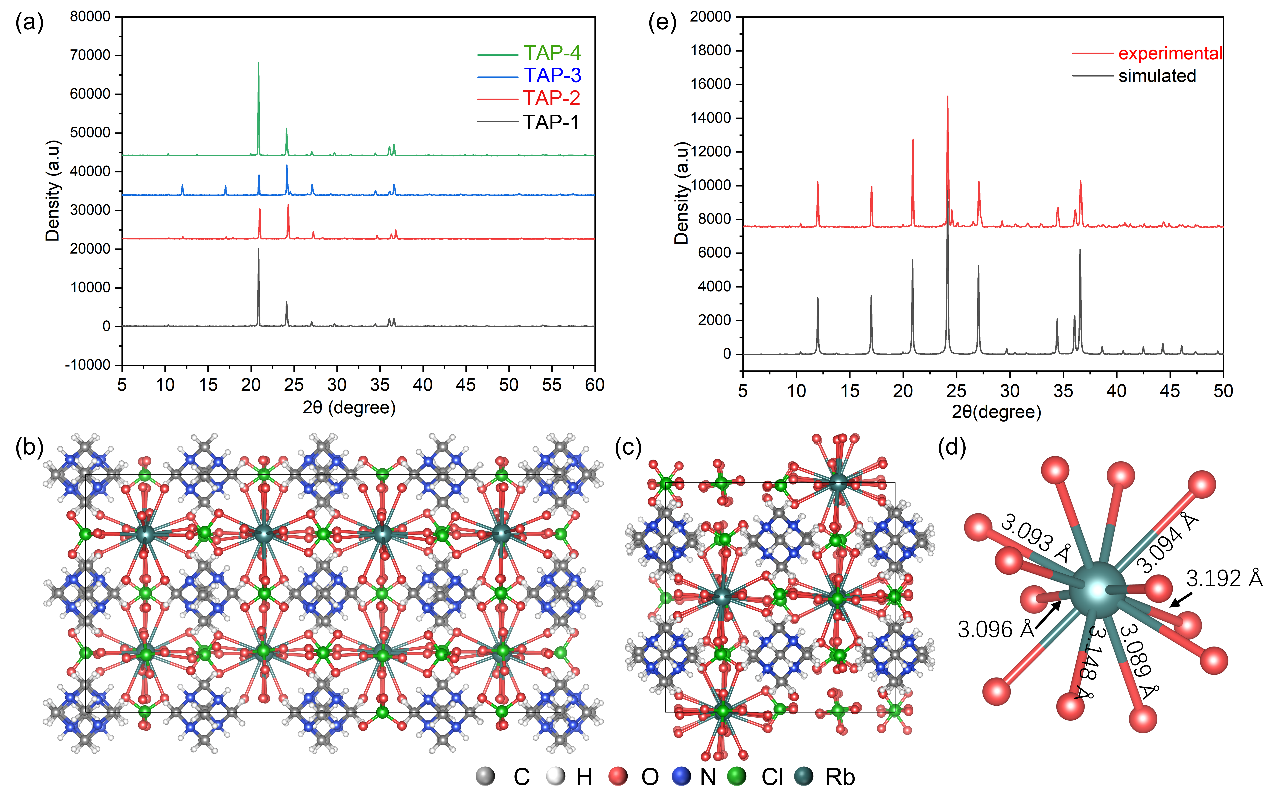


Figure S3 (a) PXRD patterns of TAPs; (b) Unit cell of TAP-3 along *b*-axis after structure optimization; (c) Unit cell of TAP-3 along *c*-axis; (d) Coordination environment of Rb^+^; (e) Experimental and simulated PXRD patterns of TAP-3.

***In-situ* reaction progress with PATs**


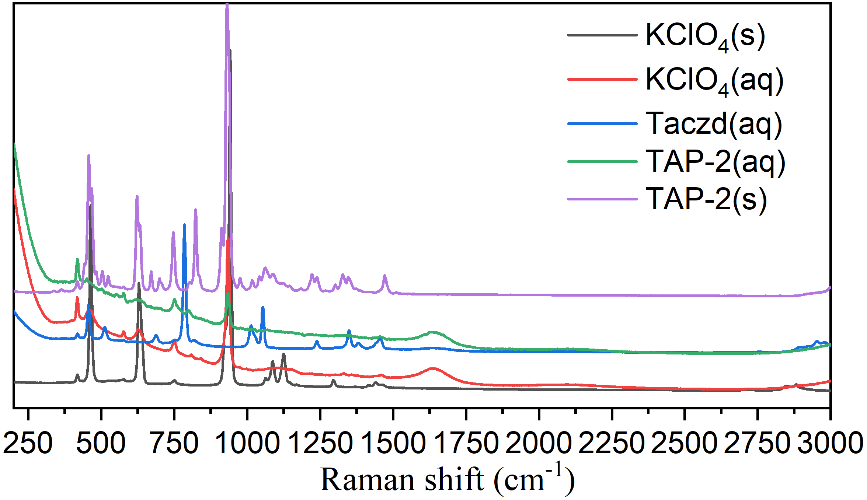


Figure S4. Raman spectra of the substances existed in the reaction process.


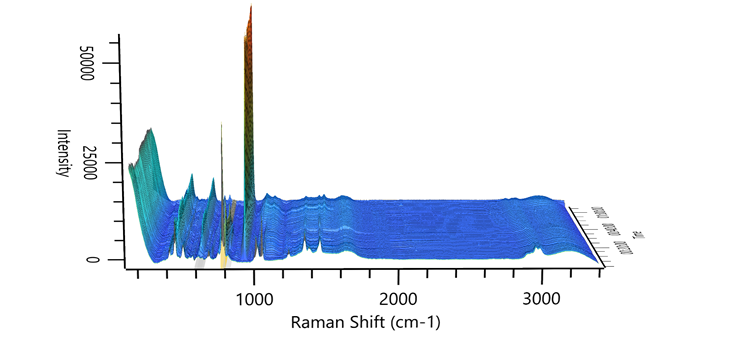


Figure S5. 3D spectra surface of the reaction progress.

**Hygroscopicity**

The hygroscopicity of TAPs was measured according to GJB 5891.9-2006. The sample is placed in an environment at 30 ℃ and 90% relative humidity until the mass change is less than 0.0002 g or a decrease occurs. The experiment is then immediately terminated.


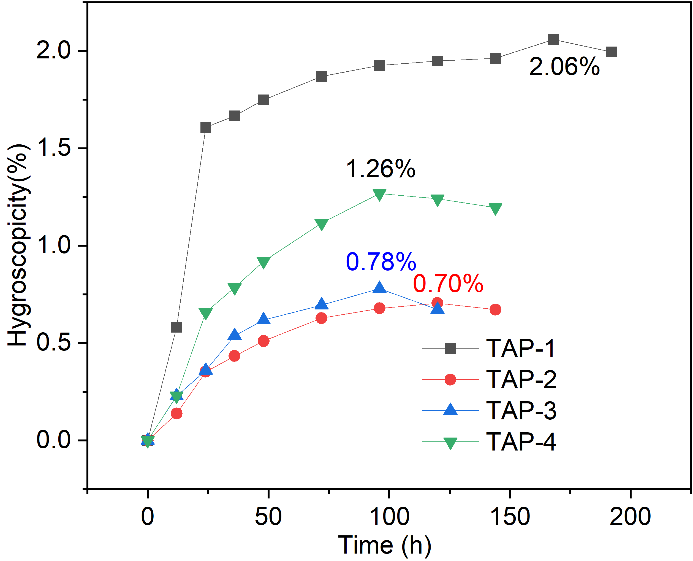


Figure S6 the hygroscopicity curve of TAPs at 90% relative humidity

As shown in Fig S6, the maximum hygroscopicity values for TAP-1, TAP-2, TAP-3, and TAP-4 are 2.02%, 0.70%, 0.78%, and 1.26%, respectively.

**Thermal Stability Analysis**

To confirm the initial decomposition temperature, the first derivative of the TG curve was performed to obtain the DTG curve.


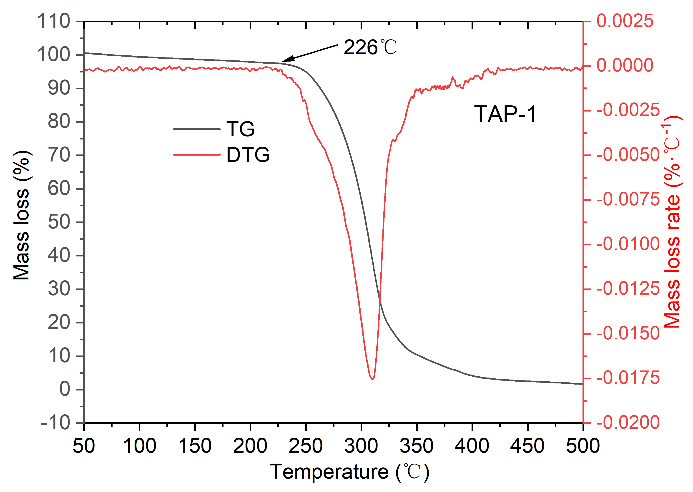


Figure S7. TG and DTG curves of TAP-1.


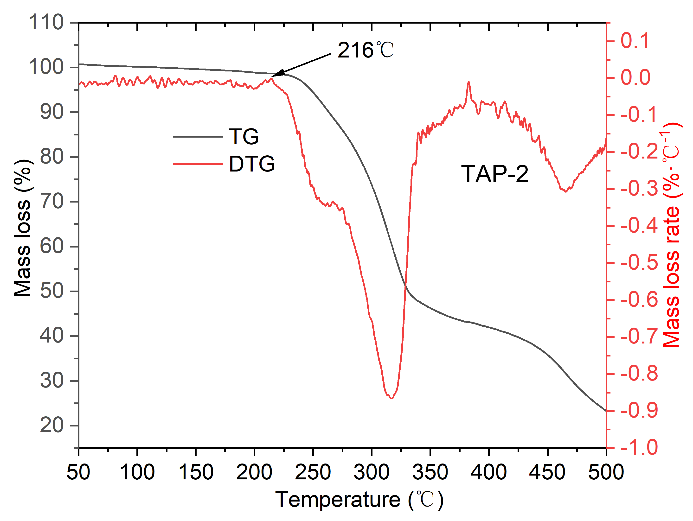


Figure S8. TG and DTG curves of TAP-2.


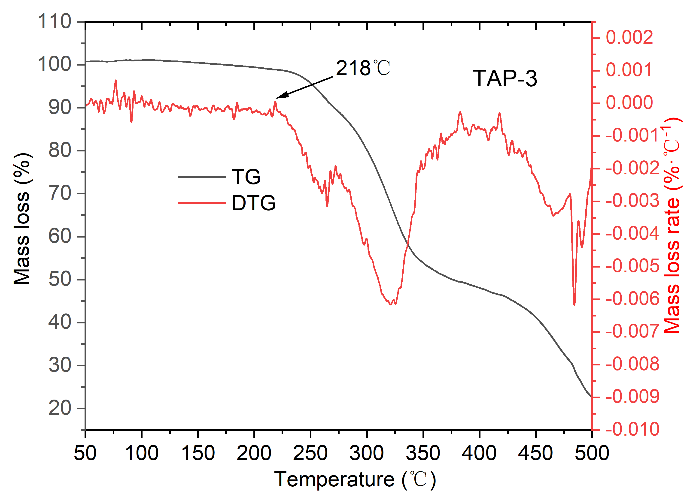


Figure S9. TG and DTG curves of TAP-3.


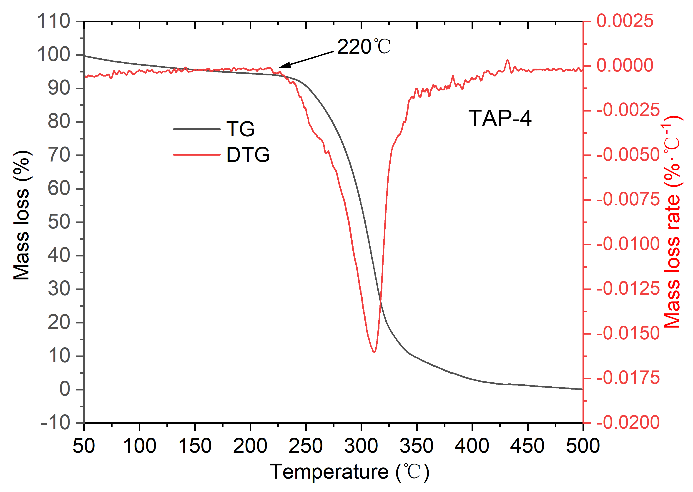


Figure S10. TG and DTG curves of TAP-4.


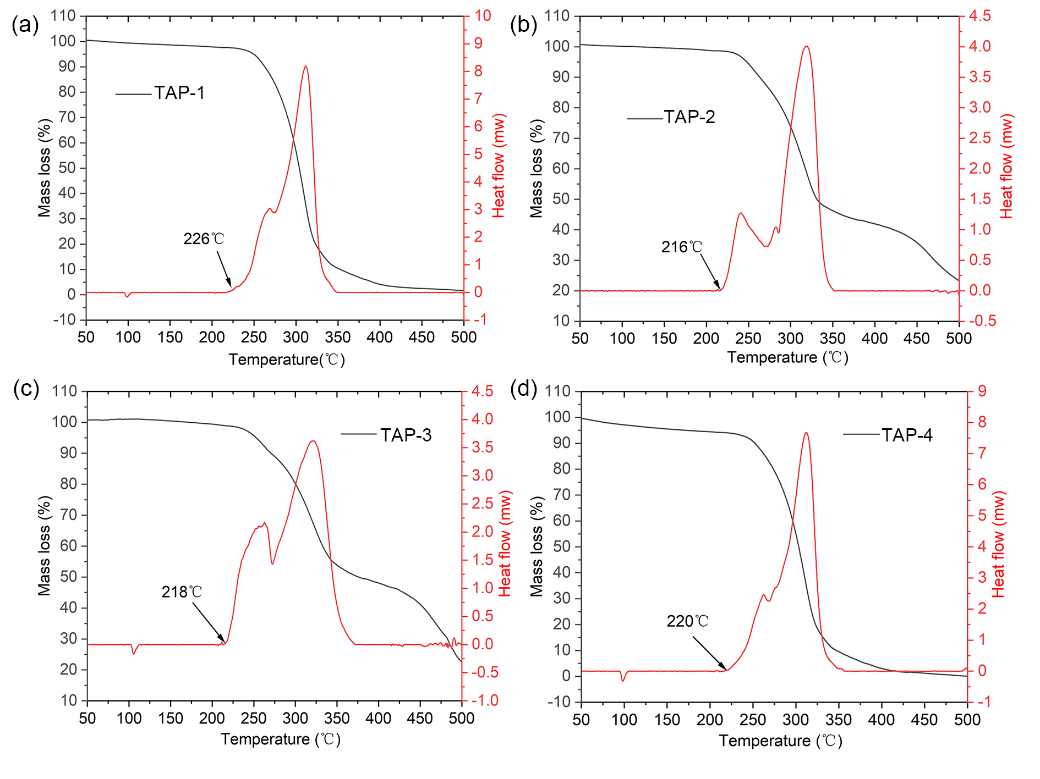


Figure S11. DSC-TG curves of the urotropine-based PEMs: (a) TAP-1; (b) TAP-2; (c) TAP-3; (d) TAP-4.

**Ionic hardness calculation**

Table S6. The distance between B-site cation and the adjacent Cl atom in the framework and the volume of framework around A-site cation in different PEMs.

| Compd | *d*_1_ [Å]^a)^ | *d*_2_ [Å]^a)^ | *d*_3_ [Å]^a)^ | *V* [Å^3^] |
| --- | --- | --- | --- | --- |
| PAP-1 | 3.479 | 3.462 | 3.321 | 329.208 |
| DAP-1 | 3.511 | 3.511 | 3.511 | 354.444 |
| TAP-1 | 3.663 | 3.631 | 3.619 | 390.434 |
| PAP-H2 | 3.725 | 3.520 | 3.594 | 374.856 |
| DAP-2 | 3.592 | 3.592 | 3.592 | 369.219 |
| DAP-O2 | 3.690 | 3.625 | 3.636 | 385.831 |
| TAP-2 | 3.616 | 3.659 | 3.633 | 383.777 |
| PAP-4 | 3.641 | 3.641 | 3.641 | 386.146 |
| PAP-H4 | 3.64 | 3.625 | 3.621 | 377.813 |
| PAP-M4 | 3.744 | 3.629 | 3.692 | 404.496 |
| DAP-4 | 3.612 | 3.612 | 3.612 | 375.273 |
| DAP-O4 | 3.690 | 3.690 | 3.690 | 402.111 |
| DAP-M4 | 3.717 | 3.740 | 3.740 | 410.736 |
| TAP-4 | 3.697 | 3.645 | 3.66 | 389.924 |

Note: ^a)^ The distance between Na atom and the adjacent Cl atom in the framework; ^b)^ the volume of framework around A-site cation.

Since Pearson proposed the HSAB theory in 1963, there is currently no physical quantity that can perfectly quantitatively evaluate the softness and hardness of acids and bases. However, chemical potential (*μ*), electronegativity (*χ*), chemical hardness (*η*), and softness (*S*) are usually used to describe the softness and hardness of Lewis acids and bases.^[11]^ Among them, *μ*, *χ*, and *η* are parameters that describe the global physicochemical properties of Lewis acids or bases, and the local softness (s) is a parameter that describes the local physicochemical properties of Lewis acids.^[12]^ In this work, Gaussian and Multiwfn 3.9 software were used to calculate density functional physical quantities related to A-site cations.^[13-14]^ The physical quantity parameters were selected to describe the softness and hardness of acids and bases, which were obtained through Equations S3-S10:

$VIP=E\left(N-1\right)-E(N)$ (S3)

$VEN=E\left(N\right)-E(N+1)$ (S4)

$\chi=(VIP+VEN)/2$ (S5)

$\mu=-\chi$ (S6)

$\eta=VIP\text{-}VEA$ (S7)

$S=1/\eta$ (S8)

$f^{-}(r)=\rho_{N}(r)-\rho_{N-1}(r)$ (S9)

$s^{-}\left(r\right)=Sf^{-}\left(r\right)$ (S10)

Among them, *VIP* is the vertical ionization potential, *VEN* is the vertical electron affinity, *f* ^-^ is the Fukui functions for electrophilic attack, and *s*^-^ is the local flexibility of electrophilic attack.

Firstly, the A-site cations were optimized using Gaussian software, and the functional and basis sets were determined using m062x/def2tzvp. Based on the optimized structure, the wave function files for A-site cation in *N*, *N*-1, and *N*+1 states were obtained at the same computational level. Among them, *N* is the number of electrons carried by a molecule in its most stable state. Based on the wave function files in *N*, *N*-1, and *N*+1 states, the density functional related physical quantities *μ*, *χ*, *η*, and *S* of ions can be easily obtained using Multiwfn 3.9 software.


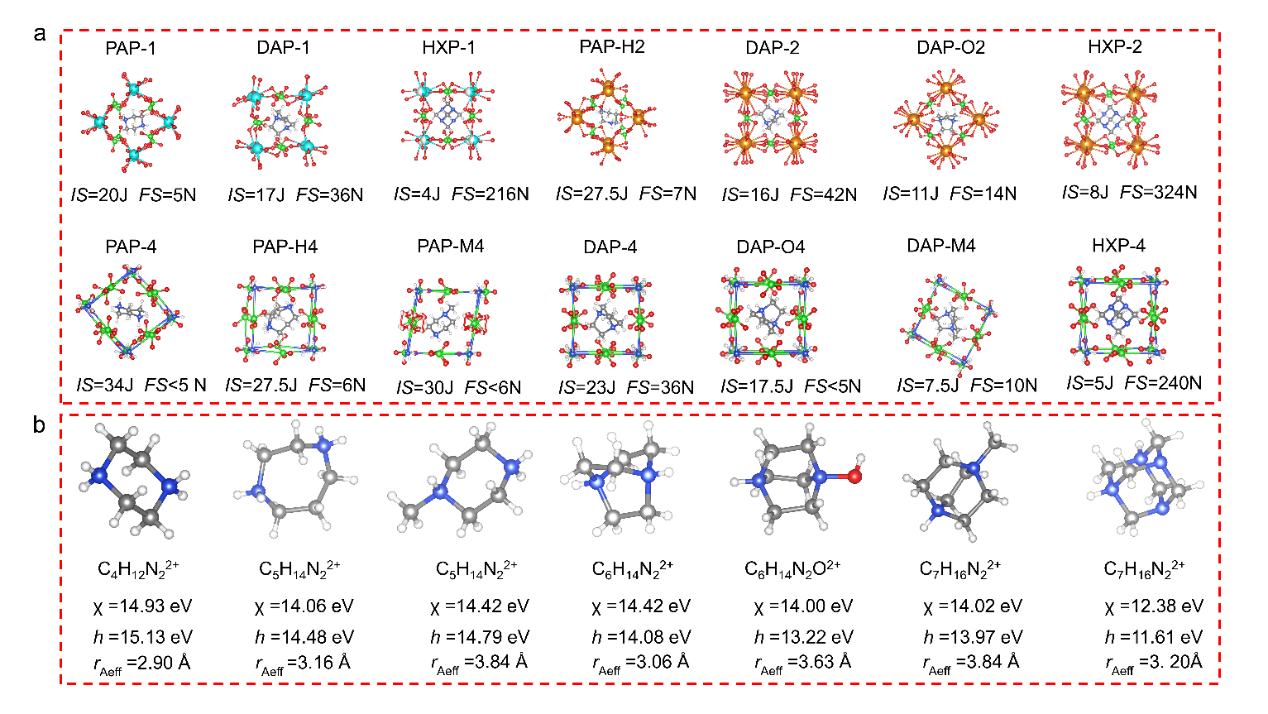


Figure S12 (a) the frameworks and corresponding mechanical sensitivities of different PEMs. (b) The Mulliken electronegativity (χ), hardness (*h*) and effective radius (*r*_Aeff_) of different A-site cations.

**Hydrogen bonds statistics**

A-site cations were optimized using Gaussian software, and the functional and basis sets were determined using m062x/def2tzvp. Based on the optimized structure, the wave function files for A-site cation were obtained at the same computational level. The different forms of charges can be easily obtained using Multiwfn 3.9 software.


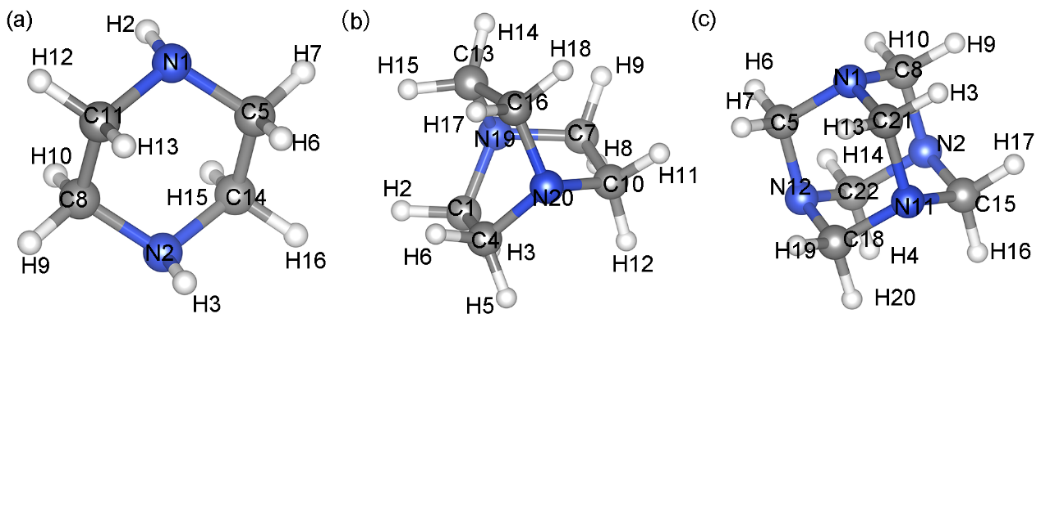


Figure S13. Three types of organic amine molecules as A-site cations: (a) Pz; (b) Dabco; (c) Tazcd.

Table S7. Hirshfeld charges and VDD charges of each atom in Pz.

| Atom | Hirshfeld charge | VDD charge | Atom | Hirshfeld charge | VDD charge |
| --- | --- | --- | --- | --- | --- |
| N1 | -0.174 | -0.205 | H9 | 0.0317 | 0.0321 |
| H2 | 0.0907 | 0.0935 | H10 | 0.0314 | 0.0341 |
| N3 | -0.175 | -0.204 | C11 | -0.0218 | -0.0107 |
| H4 | 0.0910 | 0.0933 | H12 | 0.0322 | 0.0327 |
| C5 | -0.0213 | -0.00777 | H13 | 0.0310 | 0.0327 |
| H6 | 0.0312 | 0.0321 | C14 | -0.0212 | -0.0107 |
| H7 | 0.0321 | 0.0333 | H15 | 0.0314 | 0.0330 |
| C8 | -0.0217 | -0.0102 | H16 | 0.0322 | 0.0320 |

Table S8. Hirshfeld charges and VDD charges of each atom in Dabco.

| Atom | Hirshfeld charge | VDD charge | Atom | Hirshfeld charge | VDD charge |
| --- | --- | --- | --- | --- | --- |
| C1 | -0.0203 | -0.00295 | H11 | 0.0304 | 0.0276 |
| H2 | 0.0304 | 0.0291 | H12 | 0.0325 | 0.0365 |
| H3 | 0.0325 | 0.0357 | C13 | -0.0203 | -0.00450 |
| C4 | -0.0202 | -0.00392 | H14 | 0.0304 | 0.0279 |
| H5 | 0.0304 | 0.0271 | H15 | 0.0325 | 0.0361 |
| H6 | 0.0325 | 0.0361 | C16 | -0.0202 | -0.00236 |
| C7 | -0.0203 | -0.00530 | H17 | 0.0304 | 0.0284 |
| H8 | 0.0304 | 0.0383 | H18 | 0.0325 | 0.0375 |
| H9 | 0.0325 | 0.0353 | N19 | -0.128 | -0.182 |
| C10 | -0.0201 | -0.00466 | N20 | -0.128 | -0.180 |

Table S9. Hirshfeld charges and VDD charges of each atom in Tazcd.

| Atom | Hirshfeld charge | VDD charge | Atom | Hirshfeld charge | VDD charge |
| --- | --- | --- | --- | --- | --- |
| N1 | -0.136 | -0.192 | N12 | -0.0167 | -0.231 |
| N2 | -0.167 | -0.231 | H13 | 0.0402 | 0.0482 |
| H3 | 0.0402 | 0.0482 | H14 | 0.0352 | 0.0400 |
| H4 | 0.0352 | 0.0400 | C15 | 0.0253 | 0.0527 |
| C5 | 0.0253 | 0.0527 | H16 | 0.0391 | 0.0454 |
| H6 | 0.0390 | 0.0453 | H17 | 0.0372 | 0.0429 |
| H7 | 0.0372 | 0.0429 | C18 | 0.0254 | 0.0533 |
| C8 | 0.0254 | 0.0532 | H19 | 0.0374 | 0.0422 |
| H9 | 0.0374 | 0.0422 | H20 | 0.392 | 0.0458 |
| H10 | 0.0392 | 0.0460 | N21 | 0.0320 | 0.0578 |
| N11 | -0.136 | -0.191 | N22 | 0.0170 | 0.0462 |

In order to investigate the effect of N-H···O and C-H···O hydrogen bonds on sensitivity, the hydrogen bonds of different PEMs were counted. The bond length and bond angle were measured by Mercury software. The framework units and specific data were shown in Table S10-S21.

Based on the experimentally obtained unit cell structure, the cage ion clusters of AB_8_X_12_^2-^ were selected. In Gaussian, m062x functional and def2tzvp basis set were used for optimization and the wave function file was obtained. The position of non-hydrogen atoms was fixed during the optimization process. Subsequently, Multiwfn software was used for AIM analysis to obtain the electron density at the critical point of bonding (BCP), and the bond energy of hydrogen bonds was estimated using empirical formulas (Equation S11-S12).^[15]^

$\Delta E \approx-223.08\times\rho_{\text{r}(\text{BCP})}+0.7423$ (S11)

$\Delta E \approx-332.34\times\rho_{\text{r}(\text{BCP})}-1.061$ (S12)


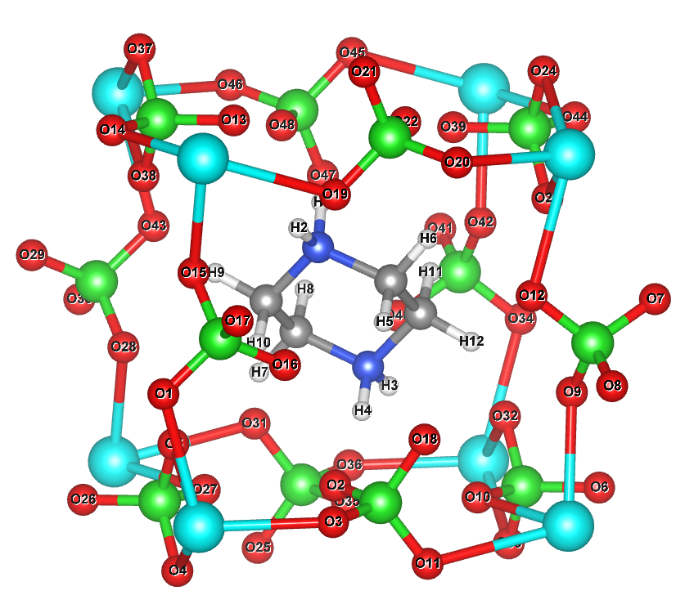


Figure S14. The framework units of PAP-1 and atom labels with hydrogen bonding.

Table S10. Hydrogen bonding and estimated bond energy between A-site cation and surrounding framework in PAP-1.

| N/C-H···O | d (H···Y) (Å) | Angle (°) | ρr(BCP) | ΔE (kcal·mol^-1)^ |
| --- | --- | --- | --- | --- |
| N35-H37···O19 | 2.340 | 124.90 | 0.0108 | -4.650 |
| N35-H37···O21 | 2.650 | 132.92 | 0.0056 | -2.922 |
| N35-H37···O28 | 2.306 | 141.79 | 0.0108 | -4.650 |
| N35-H36···O75 | 2.907 | 125.69 | 0.0032 | -2.124 |
| N35-H36···O86 | 1.930 | 154.60 | 0.0235 | -8.871 |
| N38-H39···O68 | 2.480 | 121.87 | 0.0086 | -3.919 |
| N38-H39···O76 | 2.437 | 124.21 | 0.0087 | -3.952 |
| N38-H39···O64 | 2.232 | 132.92 | 00109 | -4.684 |
| N38-H40···O3 | 1.853 | 170.59 | 0.0287 | -10.599 |
| C41-H42···O22 | 2.486 | 137.36 | 0.00802 | -1.047 |
| C41-H42···O27 | 2.398 | 142.13 | 0.00977 | -1.437 |
| C41-H43···O18 | 2.481 | 109.44 | 0.00963 | -1.406 |
| C41-H43…O31 | 2.385 | 131.37 | 0.0115 | -1.823 |
| C41-H43···O33 | 2.517 | 129.87 | 0.00863 | -1.183 |
| C44-H45···O58 | 2.412 | 147.15 | 0.00948 | -1.372 |
| C44-H45···O59 | 2.561 | 121.42 | 0.00782 | -1.002 |
| C44-H45···O62 | 2.308 | 129.08 | 0.0124 | -2.024 |
| C44-H46···O76 | 2.633 | 118.27 | 0.00682 | -0.779 |
| C44-H46···O79 | 3.072 | 104.79 | 0.00328 | 0.0106 |
| C44-H46···O85 | 2.428 | 164.49 | 0.00873 | -1.205 |
| C47-H48…O59 | 2.781 | 113.82 | 0.00552 | -0.489 |
| C47-H48···O72 | 2.298 | 145.26 | 0.0116 | -1.845 |
| C47-H48···O79 | 2.758 | 131.26 | 0.00530 | -0.440 |
| C47-H49···O7 | 2.435 | 130.85 | 0.00906 | -1.279 |
| C47-H49···O22 | 2.415 | 138.77 | 0.0104 | -1.578 |
| C50-H51···O75 | 2.751 | 126.15 | 0.00548 | -0.480 |
| C50-H51···O76 | 2.933 | 115.60 | 0.00460 | -0.284 |
| C50-H51···O78 | 2.455 | 156.19 | 0.00864 | -1.185 |
| C50-H52···O13 | 2.387 | 162.49 | 0.00974 | -1.430 |
| C50-H52···O14 | 2.941 | 117.73 | 0.00426 | -0.208 |
| C50-H52···O64 | 2.823 | 104.61 | 0.00587 | -0.567 |


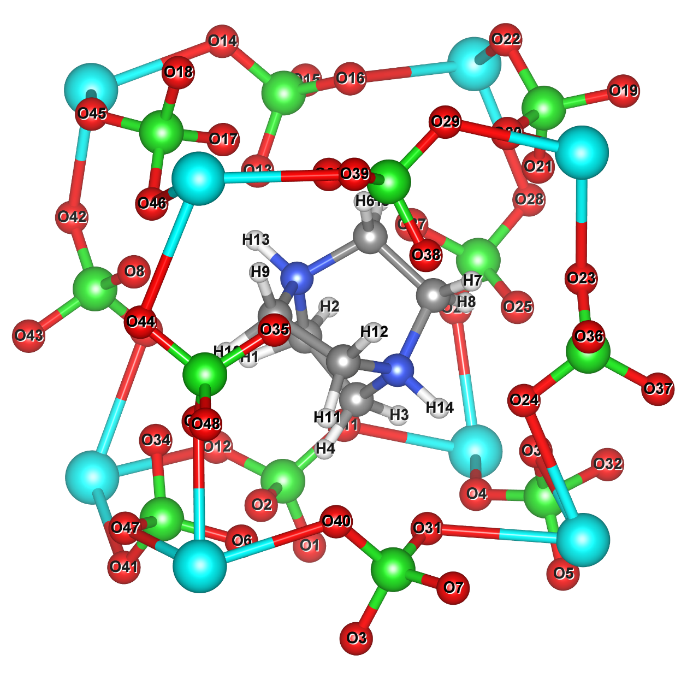


Figure S15. The framework units of DAP-1 and atom labels with hydrogen bonding.

Table S11. Hydrogen bonding and estimated bond energy between A-site cation and surrounding framework in DAP-1.

| N/C-H···O | d (H···Y) [Å] | Angle [°] | ρr(BCP) | E [kcal·mol^-1^] |
| --- | --- | --- | --- | --- |
| N62-H63···O11 | 2.244 | 123.90 | 0.0129 | -5.348 |
| N62-H63···O17 | 2.244 | 123.89 | 0.0129 | -5.348 |
| N62-H63···O22 | 2.244 | 123.89 | 0.0129 | -5.348 |
| N64-H65···O30 | 2.237 | 128.94 | 0.0126 | -5.248 |
| N64-H65···O51 | 2.237 | 128.94 | 0.0126 | -5.248 |
| N64-H65···O53 | 2.237 | 128.94 | 0.0126 | -5.248 |
| C31-H32···O3 | 2.899 | 109.68 | 0.00479 | -0.326 |
| C31-H32···O11 | 2.644 | 116.12 | 0.00778 | -0.993 |
| C31-H32···O12 | 2.360 | 139.52 | 0.0111 | -1.734 |
| C31-H32···O61 | 2.487 | 135.27 | 0.00844 | -1.140 |
| C31-H33···O14 | 2.818 | 102.32 | 0.00569 | -0.527 |
| C31-H33···O40 | 2.356 | 155.71 | 0.0107 | -1.645 |
| C34-H35···O6 | 2.210 | 155.03 | 0.0144 | -2.470 |
| C34-H35···O14 | 2.691 | 114.29 | 0.00652 | -0.712 |
| C34-H35···O53 | 2.706 | 114.86 | 0.00702 | -0.824 |
| C34-H36···O3 | 2.750 | 105.08 | 0.00605 | -0.607 |
| C34-H36···O8 | 2.286 | 171.06 | 0.0118 | -1.890 |
| C42-H43···O17 | 2.644 | 116.12 | 0.00778 | -0.993 |
| C42-H43···O20 | 2.360 | 139.51 | 0.0111 | -1.734 |
| C42-H43···O27 | 2.899 | 109.69 | 0.00479 | -0.326 |
| C42-H43···O40 | 2.487 | 135.28 | 0.00844 | -1.140 |
| C42-H44···O26 | 2.818 | 102.32 | 0.00569 | -0.527 |
| C42-H44···O50 | 2.356 | 155.72 | 0.0107 | -1.645 |
| C45-H46···O26 | 2.691 | 114.29 | 0.00652 | -0.712 |
| C45-H46···O29 | 2.210 | 155.03 | 0.0144 | -2.470 |
| C45-H46···O30 | 2.707 | 114.87 | 0.00702 | -0.824 |
| C45-H47···O27 | 2.750 | 105.07 | 0.00604 | -0.605 |
| C45-H47···O38 | 2.286 | 171.05 | 0.0118 | -1.890 |
| C54-H55···O22 | 2.644 | 116.13 | 0.00777 | -0.991 |
| C54-H55···O50 | 2.487 | 135.27 | 0.00844 | -1.140 |
| C54-H55···O69 | 2.899 | 109.68 | 0.00479 | -0.326 |
| C54-H55···O84 | 2.360 | 139.52 | 0.0111 | -1.734 |
| C54-H56···O13 | 2.818 | 102.32 | 0.00569 | -0.527 |
| C54-H56···O61 | 2.356 | 155.72 | 0.0107 | -1.644 |
| C57-H58···O13 | 2.691 | 114.29 | 0.00652 | -0.712 |
| C57-H58···O51 | 2.706 | 114.87 | 0.00702 | -0.824 |
| C57-H58···O75 | 2.210 | 155.03 | 0.0144 | -2.470 |
| C57-H59···O69 | 2.750 | 105.07 | 0.00604 | -0.605 |
| C57-H59···O73 | 2.286 | 171.06 | 0.0118 | -1.890 |


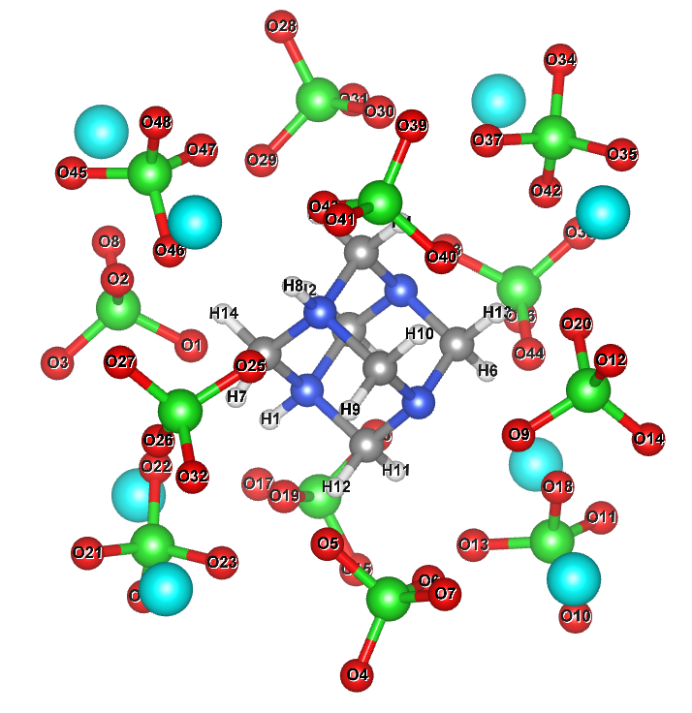


Figure S16. The framework units of TAP-1 and atom labels with hydrogen bonding.

Table S12. Hydrogen bonding and estimated bond energy between A-site cation and surrounding framework in TAP-1.

| N/C-H···O | d (H···Y) [Å] | Angle [°] | ρr(BCP) | E [kcal·mol^-1^] |
| --- | --- | --- | --- | --- |
| N17-H18···O54 | 2.237 | 128.94 | 0.0132 | -5.448 |
| N17-H18···O4 | 2.679 | 127.67 | 0.00979 | -4.315 |
| N17-H18···O30 | 2.537 | 115.88 | 0.0194 | -7.508 |
| N40-H41···O85 | 2.827 | 168.74 | 0.0194 | -7.508 |
| N40-H41···O62 | 2.408 | 144.98 | 0.00979 | -4.315 |
| N40-H41···O90 | 2.840 | 132.49 | 0.0133 | -5.481 |
| C32-H33···O4 | 2.397 | 118.05 | 0.0113 | -1.778 |
| C32-H33···O66 | 2.460 | 148.88 | 0.00864 | -1.185 |
| C32-H34···O27 | 2.347 | 150.22 | 0.0108 | -1.667 |
| C32-H34···O75 | 2.692 | 105.05 | 0.00676 | -0.766 |
| C32-H34···O86 | 2.780 | 131.35 | 0.00507 | -0.389 |
| C35-H36···O79 | 2.223 | 160.39 | 0.0137 | -2.314 |
| C35-H37···O66 | 2.702 | 140.88 | 0.00589 | -0.572 |
| C35-H37···O67 | 2.551 | 109.70 | 0.00866 | -1.190 |
| C35-H37···O91 | 2.405 | 143.73 | 0.00957 | -1.392 |
| C43-H44···O8 | 2.460 | 148.88 | 0.00864 | -1.185 |
| C43-H44···O62 | 2.397 | 118.05 | 0.0113 | -1.778 |
| C43-H45···O19 | 2.692 | 105.04 | 0.00676 | -0.766 |
| C43-H45···O31 | 2.779 | 131.35 | 0.00507 | -0.389 |
| C43-H45···O82 | 2.347 | 150.23 | 0.0108 | -1.667 |
| C46-H47···O23 | 2.223 | 160.39 | 0.0137 | -2.314 |
| C46-H48···O8 | 2.702 | 140.89 | 0.00590 | -0.574 |
| C46-H48···O9 | 2.551 | 109.69 | 0.00866 | -1.190 |
| C46-H48···O55 | 2.405 | 143.73 | 0.00957 | -1.392 |
| C57-H38···O29 | 2.421 | 119.92 | 0.0106 | -1.622 |
| C57-H38···O86 | 2.376 | 143.47 | 0.0101 | -1.511 |
| C57-H49···O31 | 2.376 | 143.46 | 0.0101 | -1.511 |
| C57-H49···O84 | 2.421 | 119.93 | 0.0106 | -1.622 |
| C58-H39···O54 | 2.564 | 102.17 | 0.00972 | -1.426 |
| C58-H39···O63 | 2.254 | 155.27 | 0.0129 | -2.135 |
| C58-H50···O5 | 2.254 | 155.27 | 0.0129 | -2.135 |
| C58-H50···O90 | 2.563 | 102.17 | 0.00972 | -1.426 |


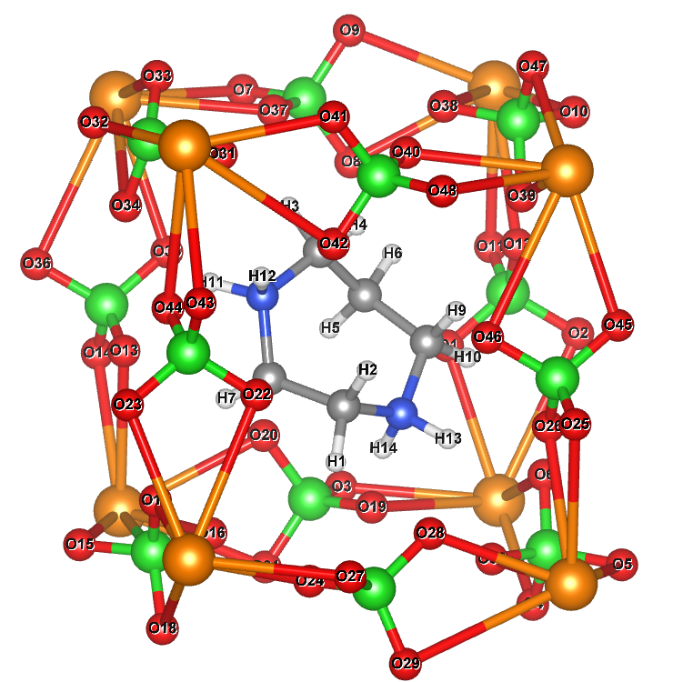


Figure S17. The framework units of PAP-H2 and atom labels with hydrogen bonding.

Table S13. Hydrogen bonding and estimated bond energy between A-site cation and surrounding framework in PAP-H2.

| N/C-H···O | d (H···Y) [Å] | Angle [°] | ρr(BCP) | E [kcal·mol^-1^] |
| --- | --- | --- | --- | --- |
| N50-H51···O71 | 2.065 | 141.57 | 0.0186 | -7.243 |
| N50-H51···O19 | 2.443 | 131.93 | 0.00849 | -3.883 |
| N50-H52···O84 | 2.324 | 122.70 | 0.0114 | -4.850 |
| N50-H52···O68 | 2.340 | 114.92 | 0.0121 | -5.082 |
| N50-H52···O82 | 2.380 | 146.18 | 0.00880 | -3.986 |
| N53-H54···O59 | 2.112 | 147.41 | 0.0159 | -6.345 |
| N53-H54···O65 | 2.347 | 126.85 | 0.0108 | -4.650 |
| N53-H55···O28 | 1.863 | 166.19 | 0.0280 | -10.367 |
| C35-H36···O56 | 2.390 | 175.96 | 0.00977 | -1.437 |
| C35-H36···O63 | 2.586 | 125.80 | 0.00758 | -0.949 |
| C35-H37···O82 | 2.843 | 142.03 | 0.00397 | -0.1439 |
| C35-H37···O87 | 2.474 | 140.62 | 0.00836 | -1.123 |
| C38-H39···O72 | 3.031 | 118.17 | 0.00319 | 0.0307 |
| C38-H39···O74 | 2.261 | 155.86 | 0.0121 | -1.957 |
| C38-H40···O76 | 3.124 | 103.59 | 0.00298 | 0.0775 |
| C38-H40···O80 | 2.353 | 164.53 | 0.0104 | -1.578 |
| C41-H42···O29 | 2.458 | 160.39 | 0.00877 | -1.214 |
| C41-H43···O2 | 2.945 | 102.24 | 0.00471 | -0.308 |
| C41-H43···O11 | 2.712 | 136.45 | 0.00524 | -0.427 |
| C41-H43···O17 | 2.597 | 136.11 | 0.00708 | -0.837 |
| C41-H43···O76 | 2.913 | 127.67 | 0.00403 | -0.157 |
| C44-H45···O25 | 2.305 | 145.19 | 0.0121 | -1.957 |
| C44-H45···O32 | 2.775 | 116.21 | 0.00596 | -0.5877 |
| C44-H45···O84 | 2.654 | 119.30 | 0.00733 | -0.893 |
| C44-H46···O19 | 2.664 | 110.30 | 0.00693 | -0.804 |
| C44-H46···O24 | 2.578 | 108.11 | 0.00873 | -1.2054 |
| C44-H46···O29 | 2.494 | 165.33 | 0.00793 | -1.027 |
| C47-H48···O77 | 2.391 | 127.96 | 0.00976 | -1.435 |
| C47-H48···O87 | 2.811 | 135.01 | 0.00509 | -0.393 |
| C47-H49···O2 | 2.722 | 126.39 | 0.00594 | -0.583 |
| C47-H49···O8 | 2.539 | 130.43 | 0.00755 | -0.942 |
| C47-H49···O17 | 2.836 | 117.30 | 0.00487 | -0.3442 |


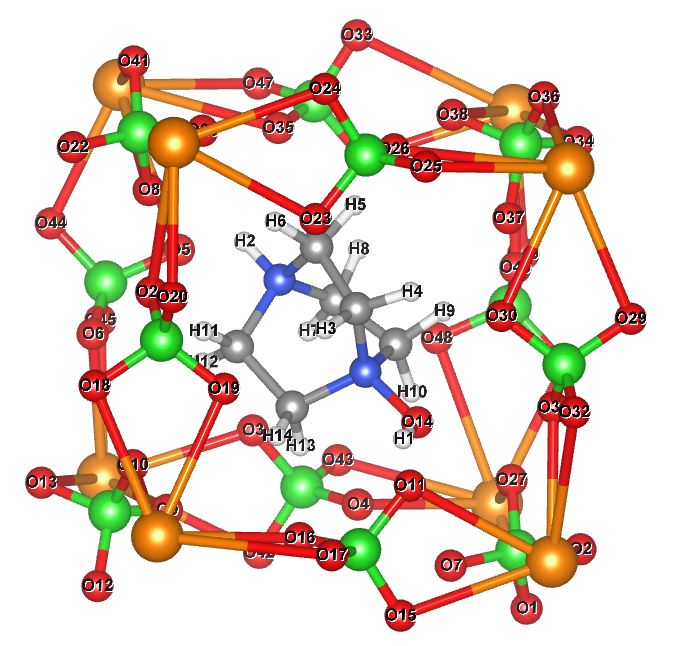


Figure S18. The framework units of DAP-O2 and atom labels with hydrogen bonding.

Table S14. Hydrogen bonding and estimated bond energy between A-site cation and surrounding framework in DAP-O2.

| N/C-H···O | d (H···Y) [Å] | Angle [°] | ρr(BCP) | E [kcal·mol^-1^] |
| --- | --- | --- | --- | --- |
| O19-H20···O16 | 1.722 | 158.02 | 0.0194 | -3.585 |
| N25-H26···O11 | 2.211 | 127.96 | 0.01334 | -5.494 |
| N25-H26···O67 | 2.332 | 124.85 | 0.0104 | -4.517 |
| N25-H26···O8 | 2.338 | 127.53 | 0.0104 | -4.517 |
| C27-H28···O47 | 2.566 | 161.90 | 0.00686 | -0.788 |
| C27-H28···O53 | 2.731 | 107.49 | 0.00619 | -0.639 |
| C27-H29···O56 | 2.788 | 104.77 | 0.00608 | -0.614 |
| C27-H29···O61 | 2.658 | 137.85 | 0.00565 | -0.518 |
| C27-H29···O69 | 2.480 | 133.82 | 0.00811 | -1.067 |
| C30-H31···O69 | 2.854 | 121.92 | 0.00482 | -0.333 |
| C30-H31···O70 | 2.541 | 174.12 | 0.00704 | -0.828 |
| C30-H32···O11 | 2.694 | 115.01 | 0.00719 | -0.862 |
| C30-H32···O49 | 2.984 | 128.83 | 0.00348 | -0.0340 |
| C30-H32···O53 | 2.900 | 112.51 | 0.00467 | -0.299 |
| C30-H32···O59 | 2.230 | 154.61 | 0.0137 | -2.314 |
| C33-H34···O5 | 2.359 | 160.34 | 0.0104 | -1.578 |
| C33-H35···O67 | 2.676 | 117.93 | 0.00715 | -0.853 |
| C33-H35···O85 | 2.283 | 153.79 | 0.0123 | -2.002 |
| C36-H37···O69 | 2.437 | 153.54 | 0.00876 | -1.212 |
| C36-H37···O73 | 2.528 | 114.39 | 0.00867 | -1.192 |
| C36-H38···O6 | 2.756 | 143.42 | 0.00481 | -0.331 |
| C36-H38···O58 | 2.453 | 142.19 | 0.00835 | -1.120 |
| C36-H38···O87 | 2.831 | 104.17 | 0.00571 | -0.531 |
| C39-H41···O5 | 2.932 | 125.57 | 0.00364 | -0.0697 |
| C39-H41···O9 | 2.318 | 141.64 | 0.0119 | -1.912 |
| C39-H41···O14 | 2.777 | 115.19 | 0.00570 | -0.529 |
| C39-H40···O49 | 2.346 | 169.49 | 0.0103 | -1.555 |
| C42-H43···O5 | 2.993 | 115.19 | 0.00349 | -0.0362 |
| C42-H43···O6 | 2.813 | 158.08 | 0.00420 | -0.1946 |
| C42-H43···O14 | 2.742 | 106.66 | 0.00607 | -0.612 |
| C42-H44···O15 | 2.690 | 109.82 | 0.00686 | -0.788 |
| C42-H44···O22 | 2.343 | 145.04 | 0.0106 | -1.622 |
| C42-H44···O47 | 2.858 | 133.42 | 0.00420 | -0.195 |


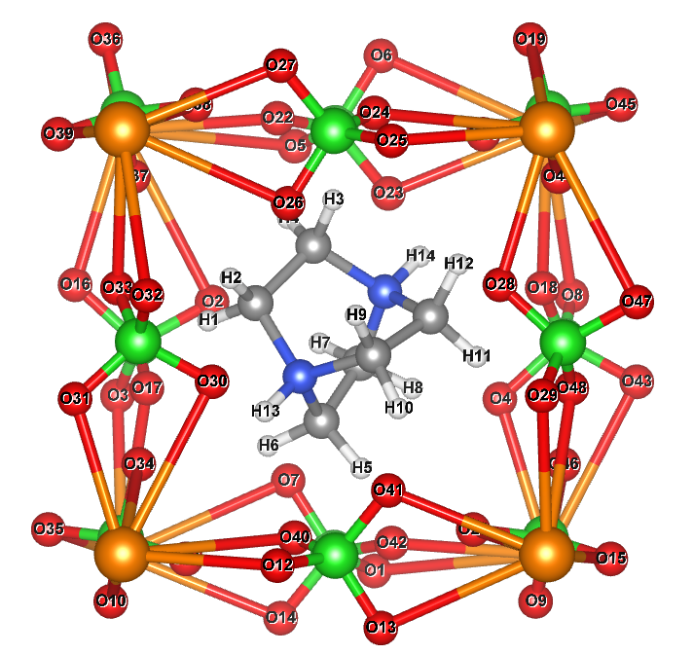


Figure S19. The framework units of DAP-2 and atom labels with hydrogen bonding.

Table S15. Hydrogen bonding and estimated bond energy between A-site cation and surrounding framework in DAP-2.

| N/C-H···O | d (H···Y) [Å] | Angle [°] | ρr(BCP) | E [kcal·mol^-1^] |
| --- | --- | --- | --- | --- |
| N76-H78···O31 | 2.408 | 129.00 | 0.00872 | -3.959 |
| N76-H78···O29 | 2.408 | 129.00 | 0.00872 | -3.959 |
| N76-H78···O41 | 2.408 | 129.00 | 0.00872 | -3.959 |
| N75-H77···O56 | 2.384 | 127.96 | 0.00927 | -4.142 |
| N75-H77···O68 | 2.384 | 127.96 | 0.00927 | -4.142 |
| N75-H77···O61 | 2.384 | 127.96 | 0.00927 | -4.142 |
| C23-H26···O45 | 2.619 | 134.39 | 0.00640 | -0.685 |
| C23-H26···O56 | 2.839 | 116.72 | 0.00534 | -0.449 |
| C23-H26···O59 | 2.385 | 141.08 | 0.0103 | -1.555 |
| C23-H26···O66 | 3.035 | 111.05 | 0.00356 | -0.0519 |
| C23-H25···O22 | 2.463 | 156.77 | 0.00849 | -1.155 |
| C23-H25···O65 | 2.815 | 102.08 | 0.00556 | -0.498 |
| C24-H27···O43 | 2.460 | 170.73 | 0.00821 | -1.089 |
| C24-H27···O66 | 2.898 | 105.50 | 0.00441 | -0.241 |
| C24-H28···O40 | 2.275 | 152.42 | 0.0125 | -2.046 |
| C24-H28···O65 | 2.749 | 112.90 | 0.00583 | -0.558 |
| C34-H36···O33 | 2.463 | 156.78 | 0.00849 | -1.152 |
| C34-H36···O70 | 2.815 | 102.07 | 0.00556 | -0.498 |
| C34-H37···O9 | 3.035 | 111.05 | 0.00356 | -0.0519 |
| C34-H37···O14 | 2.384 | 141.08 | 0.0103 | -1.555 |
| C34-H37···O22 | 2.619 | 134.39 | 0.00640 | -0.685 |
| C34-H37···O61 | 2.839 | 116.72 | 0.00534 | -0.449 |
| C35-H38···O2 | 2.460 | 170.73 | 0.00821 | -1.089 |
| C35-H38···O9 | 2.898 | 105.50 | 0.00441 | -0.241 |
| C35-H39···O4 | 2.275 | 152.43 | 0.0125 | -2.046 |
| C35-H39···O70 | 2.749 | 112.91 | 0.00583 | -0.558 |
| C47-H49···O45 | 2.463 | 156.78 | 0.00849 | -1.152 |
| C47-H49···O53 | 2.815 | 102.07 | 0.00556 | -0.498 |
| C47-H50···O33 | 2.619 | 134.39 | 0.00640 | -0.685 |
| C47-H50···O68 | 2.839 | 116.71 | 0.00534 | -0.449 |
| C47-H50···O69 | 2.385 | 141.07 | 0.0103 | -1.555 |
| C47-H50···O86 | 3.035 | 111.05 | 0.00356 | -0.0519 |
| C48-H51···O83 | 2.460 | 170.73 | 0.00821 | -1.089 |
| C48-H51···O86 | 2.898 | 105.50 | 0.00441 | -0.241 |
| C48-H52···O53 | 2.749 | 112.90 | 0.00583 | -0.558 |
| C48-H52···O81 | 2.275 | 152.42 | 0.0125 | -2.046 |


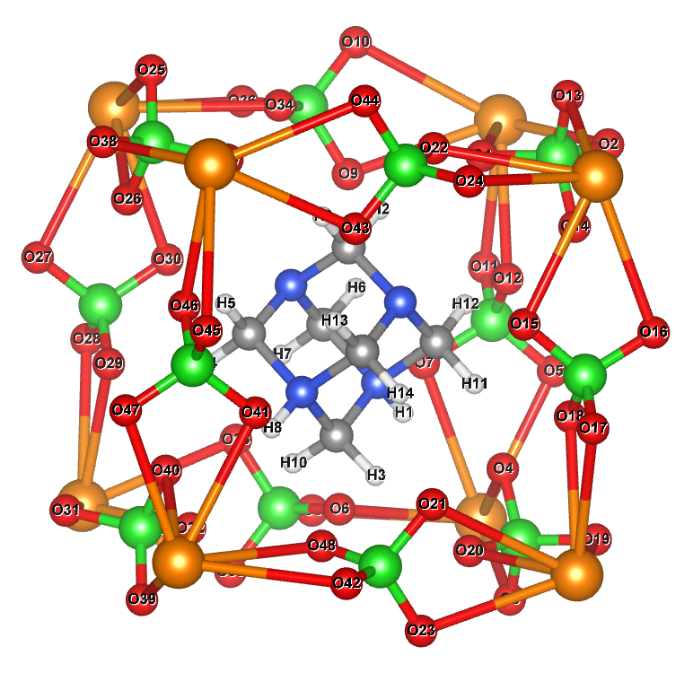


Figure S20. The framework units of TAP-2 and atom labels with hydrogen bonding.

Table S16. Hydrogen bonding and estimated bond energy between A-site cation and surrounding framework in TAP-2.

| N/C-H···O | d (H···Y) [Å] | Angle [°] | ρr(BCP) | E [kcal·mol^-1^] |
| --- | --- | --- | --- | --- |
| N60-H61···O9 | 2.420 | 121.66 | 0.00914 | -4.099 |
| N60-H61···O10 | 2.646 | 123.93 | 0.00588 | -3.015 |
| N60-H61···O5 | 2.072 | 133.93 | 0.0184 | -7.176 |
| N81-H82···O57 | 2.072 | 133.38 | 0.0184 | -7.176 |
| N81-H82···O70 | 2.646 | 123.93 | 0.00588 | -3.015 |
| N81-H82···O79 | 2.420 | 121.66 | 0.00914 | -4.099 |
| C64-H65···O40 | 2.265 | 150.31 | 0.0126 | -2.069 |
| C64-H65···O57 | 2.644 | 104.06 | 0.00842 | -1.1369 |
| C64-H66···O36 | 2.686 | 113.76 | 0.00639 | -0.6839 |
| C64-H66···O54 | 2.704 | 132.41 | 0.00592 | -0.5789 |
| C64-H66···O75 | 2.452 | 148.15 | 0.00844 | -1.1409 |
| C67-H68···O10 | 2.716 | 111.72 | 0.00639 | -0.6839 |
| C67-H68···O12 | 2.346 | 141.00 | 0.0103 | -1.5559 |
| C67-H68···O15 | 2.897 | 137.05 | 0.00416 | -0.186 |
| C67-H69···O43 | 2.737 | 107.89 | 0.00601 | -0.598 |
| C67-H69···O48 | 2.404 | 156.55 | 0.00940 | -1.355 |
| C85-H86···O5 | 2.644 | 104.06 | 0.00842 | -1.136 |
| C85-H86···O24 | 2.265 | 150.31 | 0.0126 | -2.069 |
| C85-H87···O2 | 2.704 | 132.40 | 0.00592 | -0.578 |
| C85-H87···O15 | 2.452 | 148.15 | 0.00845 | -1.143 |
| C85-H87···O18 | 2.686 | 113.75 | 0.00639 | -0.6833 |
| C88-H89···O70 | 2.716 | 111.73 | 0.00638 | -0.681 |
| C88-H89···O72 | 2.346 | 140.99 | 0.0103 | -1.555 |
| C88-H89···O75 | 2.897 | 137.06 | 0.00416 | -0.186 |
| C88-H90···O21 | 2.737 | 107.89 | 0.00601 | -0.598 |
| C88-H90···O31 | 2.404 | 156.55 | 0.00941 | -1.357 |
| C91-H62···O2 | 2.164 | 149.71 | 0.0152 | -2.649 |
| C91-H62···O49 | 2.937 | 111.17 | 0.00386 | -0.119 |
| C91-H83···O32 | 2.937 | 111.17 | 0.00386 | -0.119 |
| C91-H83···O54 | 2.164 | 149.71 | 0.0152 | -2.649 |
| C92-H63···O28 | 2.205 | 147.66 | 0.0144 | -2.4709 |
| C92-H63···O79 | 2.549 | 112.36 | 0.00881 | -1.223 |
| C92-H84···O9 | 2.549 | 112.36 | 0.00881 | -1.223 |
| C92-H84···O46 | 2.205 | 147.66 | 0.0144 | -2.470 |

**
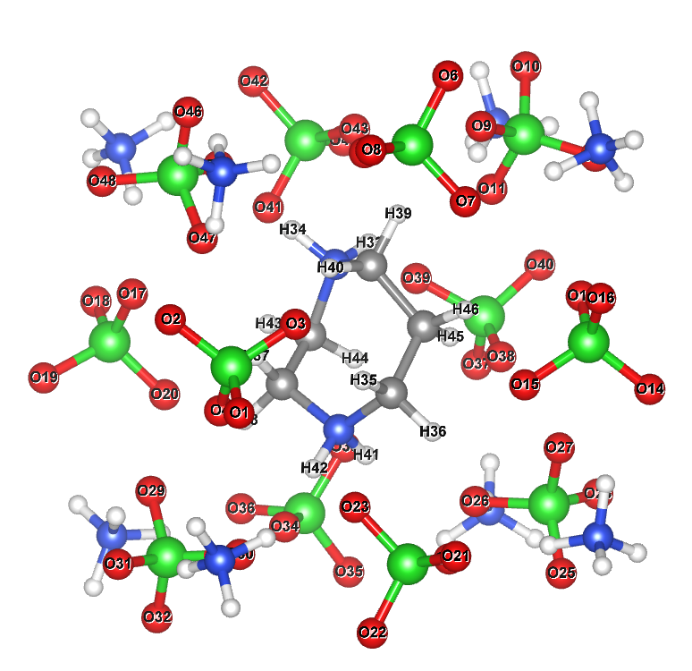
**

Figure S21. The framework units of PAP-H4 and atom labels with hydrogen bonding.

Table S17. Hydrogen bonding and estimated bond energy between A-site cation and surrounding framework in PAP-H4.

| N/C-H···O | d (H···Y) [Å] | Angle [°] | ρr(BCP) | E [kcal·mol^-1^] |
| --- | --- | --- | --- | --- |
| N101-H102···O84 | 2.352 | 123.35 | 0.0111 | -4.750 |
| N101-H102···O79 | 2.367 | 136.48 | 0.00960 | -4.251 |
| N101-H102···O14 | 2.586 | 126.48 | 0.00609 | -3.085 |
| N101-H103···O92 | 1.868 | 173.66 | 0.0279 | -10.333 |
| N113-H115···O48 | 2.115 | 152.35 | 0.0139 | -5.681 |
| N113-H115···O39 | 2.578 | 123.22 | 0.00670 | -3.288 |
| N113-H114···O43 | 2.231 | 143.16 | 0.0960 | -4.251 |
| N113-H114···O53 | 2.735 | 116.03 | 0.00508 | -2.749 |
| C104-H105···O4 | 2.321 | 163.11 | 0.0114 | -1.801 |
| C104-H106···O29 | 2.815 | 111.56 | 0.00473 | -0.313 |
| C104-H106···O40 | 2.479 | 141.97 | 0.00909 | -1.285 |
| C104-H106···O43 | 2.888 | 109.91 | 0.00520 | -0.418 |
| C104-H106···O44 | 2.600 | 131.42 | 0.00726 | -0.877 |
| C107-H108···O5 | 2.526 | 125.62 | 0.00830 | -1.109 |
| C107-H108···O94 | 2.606 | 147.56 | 0.00616 | -0.632 |
| C107-H109···O35 | 2.731 | 126.23 | 0.00552 | -0.489 |
| C107-H109···O47 | 2.301 | 139.27 | 0.0121 | -1.957 |
| C107-H109···O53 | 2.625 | 122.01 | 0.00719 | -0.862 |
| C110-H111···O7 | 2.632 | 110.81 | 0.00721 | -0.866 |
| C110-H111···O12 | 2.325 | 163.37 | 0.0113 | -1.779 |
| C110-H111···O14 | 2.499 | 115.90 | 0.0101 | -1.511 |
| C110-H112···O4 | 2.795 | 144.10 | 0.00458 | -0.279 |
| C110-H112···O94 | 2.485 | 136.99 | 0.00894 | -1.252 |
| C116-H117···O32 | 2.665 | 129.73 | 0.00663 | -0.737 |
| C116-H117···O35 | 2.535 | 134.02 | 0.00796 | -1.033 |
| C116-H117…O82 | 2.552 | 119.76 | 0.00766 | -0.966 |
| C116-H118···O52 | 2.467 | 125.89 | 0.00899 | -1.263 |
| C116-H118···O79 | 2.583 | 115.77 | 0.00833 | -1.116 |
| C119-H120···O78 | 2.506 | 174,72 | 0.00742 | -0.913 |
| C119-H121···O27 | 2.351 | 156.91 | 0.0106 | -1.622 |

**
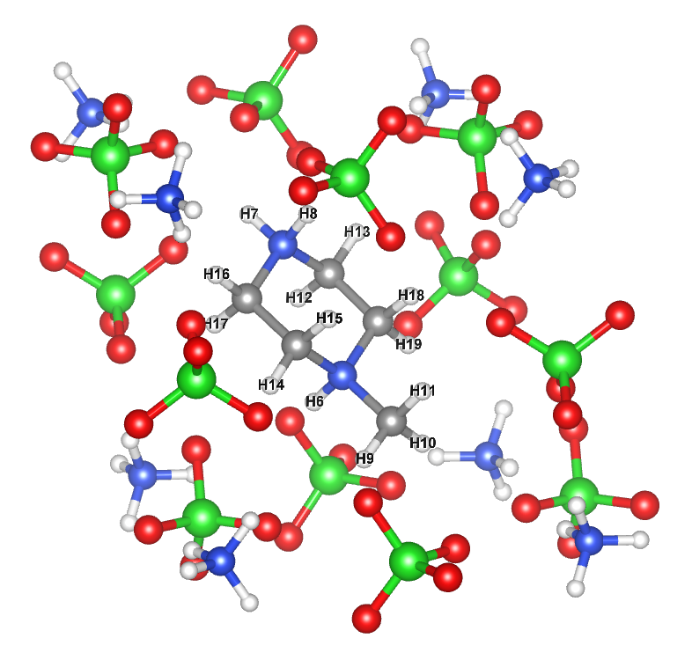
**

Figure S22. The framework units of PAP-M4 and atom labels with hydrogen bonding.

Table S18. Hydrogen bonding and estimated bond energy between A-site cation and surrounding framework in PAP-M4.

| N/C-H···O | d (H···Y) [Å] | Angle [°] | ρr(BCP) | E [kcal·mol^-1^] |
| --- | --- | --- | --- | --- |
| N23-H24···O44 | 2.011 | 149.17 | 0.0208 | -7.974 |
| N23-H24···O8 | 2.597 | 124.01 | 0.00674 | -3.301 |
| N25-H26···O117 | 2.098 | 137.93 | 0.0168 | -6.644 |
| N25-H26···O68 | 2.262 | 125.08 | 0.0120 | -5.049 |
| N25-H26···O114 | 2.627 | 119.45 | 0.00574 | -2.969 |
| N25-H27···O93 | 1.854 | 152.87 | 0.0290 | -10.699 |
| C28-H29···O9 | 2.801 | 133.31 | 0.00473 | -0.313 |
| C28-H29···O54 | 2.387 | 139.49 | 0.00987 | -1.459 |
| C28-H29···O57 | 2.878 | 110.20 | 0.00456 | -0.275 |
| C28-H30···O48 | 2.569 | 130.59 | 0.00718 | -0.859 |
| C28-H30···O59 | 2.542 | 149.28 | 0.00649 | -0.705 |
| C28-H31···O3 | 2.411 | 171.54 | 0.00955 | -1.388 |
| C32-H33···O44 | 2.514 | 127.36 | 0.00824 | -1.096 |
| C32-H33···O68 | 2.699 | 115.01 | 0.00650 | -0.708 |
| C32-H34···O19 | 2.900 | 124.84 | 0.00428 | -0.212 |
| C32-H34···O103 | 2.527 | 131.88 | 0.00826 | -1.100 |
| C32-H34···O108 | 2.582 | 121.69 | 0.00759 | -0.951 |
| C35-H36···O8 | 2.621 | 109.22 | 0.00843 | -1.138 |
| C35-H36···O84 | 2.695 | 132.17 | 0.00580 | -0.552 |
| C35-H36···O85 | 2.756 | 105.64 | 0.00628 | -0.657 |
| C35-H37···O89 | 2.320 | 162.10 | 0.0115 | -1.823 |
| C38-H39···O85 | 2.544 | 124.83 | 0.00716 | -0.855 |
| C38-H39···O110 | 2.433 | 156.11 | 0.00883 | -1.227 |
| C38-H40···O8 | 2.551 | 121.07 | 0.00728 | -0.882 |
| C38-H40···O68 | 2.675 | 114.67 | 0.00740 | -0.908 |
| C38-H40···O70 | 2.290 | 158.81 | 0.0122 | -1.979 |
| C41-H42···O87 | 2.359 | 142.19 | 0.0101 | -1.511 |
| C41-H43···O10 | 3.802 | 145.14 | 0.000566 | 0.616 |
| C41-H43···O22 | 2.462 | 129.38 | 0.00932 | -1.337 |

**
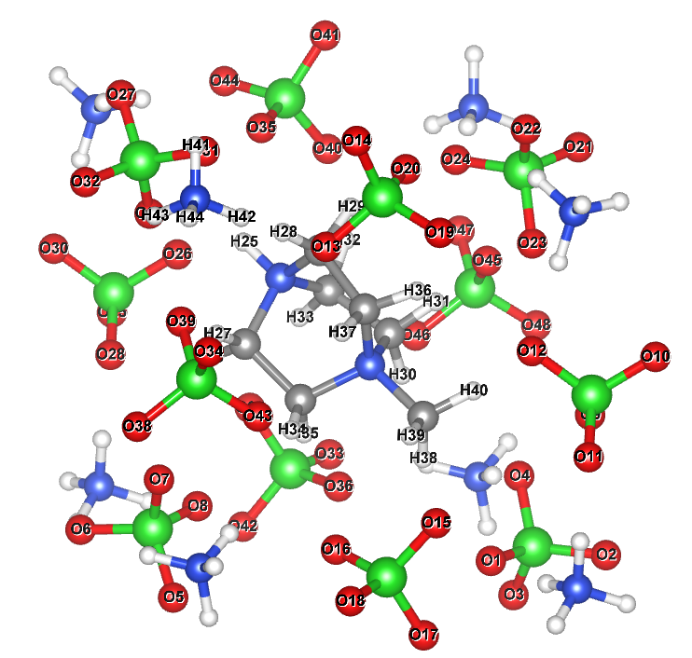
**

Figure S23. The framework units of DAP-M4 and atom labels with hydrogen bonding.

Table S19. Hydrogen bonding and estimated bond energy between A-site cation and surrounding framework in DAP-M4.

| N/C-H···O | d (H···Y) [Å] | Angle [°] | ρr(BCP) | E [kcal·mol^-1^] |
| --- | --- | --- | --- | --- |
| N72-H73···O62 | 2.236 | 125.91 | 0.0132 | -5.448 |
| N72-H73···O101 | 2.322 | 127.35 | 0.0106 | -4.584 |
| N72-H73···O59 | 2.321 | 122.43 | 0.0110 | -4.717 |
| C74-H75···O59 | 2.746 | 116.30 | 0.00645 | -0.697 |
| C74-H75···O61 | 2.269 | 157.65 | 0.0125 | -2.0467 |
| C74-H76···O62 | 2.714 | 105.00 | 0.00685 | -0.786 |
| C74-H76···O105 | 2.329 | 166.63 | 0.0109 | -1.689 |
| C77-H78···O33 | 2.696 | 114.04 | 0.00664 | -0.739 |
| C77-H78···O64 | 2.312 | 150.54 | 0.0117 | -1.868 |
| C77-H79···O50 | 2.434 | 151.90 | 0.00907 | -1.281 |
| C80-H81···O10 | 3.005 | 133.18 | 0.00289 | 0.0976 |
| C80-H81···O102 | 2.726 | 138.69 | 0.00548 | -0.480 |
| C80-H81···O118 | 2.727 | 114.65 | 0.00618 | -0.636 |
| C80-H82···O49 | 2.526 | 159.40 | 0.00763 | -0.960 |
| C80-H82···O50 | 2.799 | 132.68 | 0.00507 | -0.389 |
| C80-H82···O117 | 2.515 | 110.87 | 0.00910 | -1.288 |
| C83-H84···O50 | 2.974 | 121.76 | 0.00355 | -0.0496 |
| C83-H84···O106 | 2.252 | 149.04 | 0.0133 | -2.225 |
| C83-H85···O59 | 2.680 | 108.20 | 0.00699 | -0.817 |
| C83-H85···O103 | 2.341 | 160.40 | 0.0106 | -1.622 |
| C86-H87···O14 | 2.743 | 111.43 | 0.00613 | -0.625 |
| C86-H87···O36 | 2.652 | 135.63 | 0.00561 | -0.509 |
| C86-H87···O109 | 2.600 | 141.38 | 0.0066 | -0.730 |
| C86-H88···O15 | 2.526 | 113.85 | 0.00874 | -1.207 |
| C86-H88···O102 | 2.450 | 163.08 | 0.00865 | -1.187 |
| C89-H90···O25 | 2.892 | 133.11 | 0.00358 | -0.0563 |
| C89-H90···O45 | 2.798 | 107.55 | 0.00595 | -0.585 |
| C89-H90···O49 | 2.672 | 143.29 | 0.00358 | -0.0563 |
| C89-H91···O33 | 2.504 | 112.04 | 0.00954 | -1.386 |
| C89-H91···O109 | 2.664 | 155.28 | 0.00580 | -0.551 |
| C92-H93···O7 | 2.392 | 132.58 | 0.0105 | -1.600 |
| C92-H93···O10 | 2.664 | 123.75 | 0.00666 | -0.743 |
| C92-H94···O35 | 2.406 | 144.26 | 0.00987 | -1.459 |
| C92-H94···O36 | 2.654 | 120.99 | 0.00709 | -0.839 |
| C92-H95···O22 | 2.376 | 139.61 | 0.0106 | -1.622 |
| C92-H95···O25 | 2.655 | 121.17 | 0.00704 | -0.828 |

**
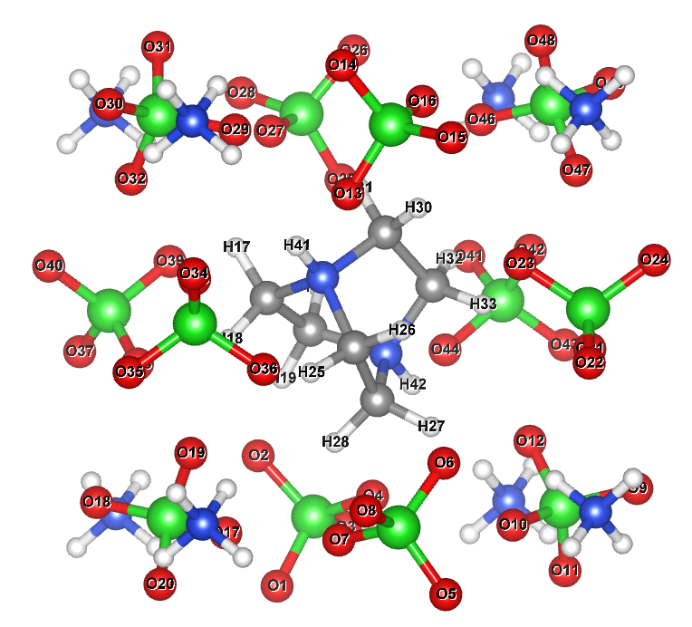
**

Figure S24. The framework units of DAP-4 and atom labels with hydrogen bonding.

Table S20. Hydrogen bonding and estimated bond energy between A-site cation and surrounding framework in DAP-4.

| N/C-H···O | d (H···Y) [Å] | Angle [°] | ρr(BCP) | E [kcal·mol^-1^] |
| --- | --- | --- | --- | --- |
| N101-H102···O83 | 2.360 | 130.04 | 0.00970 | -4.285 |
| N101-H102···O35 | 2.344 | 123.54 | 0.0100 | -4.384 |
| N101-H102···O89 | 2.359 | 173.07 | 0.00974 | -4.298 |
| N103-H104···O117 | 2.329 | 128.71 | 0.0103 | -4.484 |
| N103-H104···O30 | 2.338 | 128.79 | 0.0107 | -4.617 |
| N103-H104···O12 | 2.332 | 128.66 | 0.0103 | -4.484 |
| C41-H42···O78 | 2.648 | 132.73 | 0.00600 | -0.596 |
| C41-H42···O86 | 2.321 | 140.99 | 0.0119 | -1.912 |
| C41-H42···O96 | 3.036 | 111.12 | 0.00369 | -0.0809 |
| C41-H43···O50 | 2.509 | 157.14 | 0.00775 | -0.987 |
| C44-H45···O10 | 2.317 | 149.67 | 0.0115 | -1.8237 |
| C44-H45···O12 | 2.775 | 116.73 | 0.00602 | -0.601 |
| C44-H45···O95 | 2.603 | 112.38 | 0.00789 | -1.018 |
| C44-H46···O76 | 2.572 | 174.23 | 0.00656 | -0.721 |
| C44-H46···O78 | 2.942 | 124.15 | 0.00400 | -0.150 |
| C44-H46···O96 | 2.916 | 104.03 | 0.00441 | -0.241 |
| C56-H57···O20 | 3.063 | 111.07 | 0.00353 | -0.0452 |
| C56-H57···O50 | 2.649 | 132.69 | 0.00600 | -0.596 |
| C56-H57···O92 | 2.321 | 141.05 | 0.0119 | -1.912 |
| C56-H58···O65 | 2.515 | 158.22 | 0.00765 | -0.964 |
| C59-H60···O19 | 2.589 | 112.00 | 0.00820 | -1.087 |
| C59-H60···O28 | 2.311 | 150.36 | 0.0117 | -1.868 |
| C59-H60···O30 | 2.782 | 116.96 | 0.00595 | -0.585 |
| C59-H61···O20 | 2.935 | 104.15 | 0.00423 | -0.201 |
| C59-H61···O48 | 2.573 | 174.39 | 0.00655 | -0.719 |
| C59-H61···O50 | 2.939 | 124.24 | 0.00402 | -0.154 |
| C68-H69···O35 | 2.787 | 116.91 | 0.00589 | -0.572 |
| C68-H69···O38 | 2.343 | 140.52 | 0.0113 | -1.779 |
| C68-H69···O65 | 2.674 | 133.27 | 0.00569 | -0.527 |
| C68-H69···O121 | 3.054 | 111.39 | 0.00351 | -0.0407 |
| C68-H70···O78 | 2.509 | 157.11 | 0.00775 | -0.987 |
| C71-H72···O115 | 2.316 | 149.65 | 0.0116 | -1.845 |
| C71-H72···O117 | 2.774 | 116.74 | 0.00604 | -0.605 |
| C71-H72···O120 | 2.585 | 112.25 | 0.0116 | -1.845 |
| C71-H73···O63 | 2.528 | 173.79 | 0.00717 | -0.857 |
| C71-H73···O121 | 2.936 | 104.25 | 0.00420 | -0.195 |

**
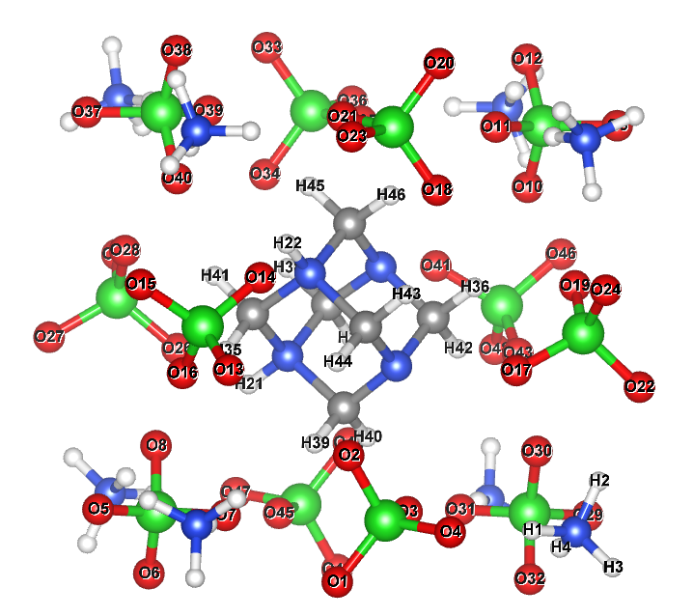
**

Figure S25. The framework units of TAP-4 and atom labels with hydrogen bonding.

Table S21. Hydrogen bonding and estimated bond energy between A-site cation and surrounding framework in TAP-4.

| N/C-H···O | d (H···Y) [Å] | Angle [°] | ρr(BCP) | E [kcal·mol^-1^] |
| --- | --- | --- | --- | --- |
| N114-H67···O82 | 2.425 | 119.68 | 0.00940 | -4.185 |
| N114-H67···O28 | 2.343 | 129.16 | 0.0105 | -4.551 |
| N114-H67···O42 | 2.692 | 120.20 | 0.00565 | -2.939 |
| N104-H56···O20 | 2.425 | 119.68 | 0.00940 | -4.185 |
| N104-H56···O59 | 2.343 | 129.16 | 0.0105 | -4.551 |
| N104-H56···O94 | 2.692 | 120.20 | 0.00565 | -2.939 |
| C107-H108···O90 | 2.646 | 112.94 | 0.00670 | -0.752 |
| C107-H108···O91 | 2.384 | 150.10 | 0.00968 | -1.417 |
| C107-H109···O59 | 2.715 | 107.70 | 0.00684 | -0.784 |
| C107-H109···O70 | 2.411 | 145.41 | 0.00937 | -1.348 |
| C110-H111···O9 | 2.655 | 109.59 | 0.00681 | -0.777 |
| C110-H111···O19 | 2.374 | 163.45 | 0.00998 | -1.484 |
| C110-H112···O65 | 2.349 | 147.76 | 0.0105 | -1.600 |
| C110-H112···O94 | 2.559 | 114.97 | 0.00843 | -1.138 |
| C117-H118···O38 | 2.646 | 112.94 | 0.00670 | -0.752 |
| C117-H118···O39 | 2.384 | 150.11 | 0.00968 | -1.417 |
| C117-H119···O8 | 2.411 | 145.11 | 0.00937 | -1.348 |
| C117-H119···O28 | 2.715 | 107.70 | 0.00684 | -0.784 |
| C120-H121···O71 | 2.655 | 109.59 | 0.00681 | -0.777 |
| C120-H121···O81 | 2.374 | 163.46 | 0.00998 | -1.484 |
| C120-H122···O24 | 2.349 | 147.76 | 0.0105 | -1.600 |
| C120-H122···O42 | 2.559 | 114.97 | 0.00843 | -1.138 |
| C123-H105···O20 | 2.444 | 115.18 | 0.0106 | -1.622 |
| C123-H105···O30 | 2.474 | 148.04 | 0.00815 | -1.076 |
| C123-H115···O61 | 2.474 | 148.03 | 0.00815 | -1.076 |
| C123-H115···O82 | 2.444 | 115.19 | 0.0106 | -1.622 |
| C124-H106···O23 | 2.635 | 109.67 | 0.00728 | -0.882 |
| C124-H106···O40 | 2.352 | 149.87 | 0.0103 | -1.555 |
| C124-H116···O64 | 2.635 | 109.67 | 0.00728 | -0.882 |
| C124-H116···O92 | 2.352 | 149.88 | 0.0103 | -1.555 |

Table S22. Hydrogen bonds and highest bond energy along different C-H directions in the cage frame of PAP-1, DAP-1 and TAP-1.

| Compd | C-H···O | d (H···Y) [Å] | Angle [°] | E [kcal·mol^-1^] |
| --- | --- | --- | --- | --- |
| PAP-1 | C41-H42···O27 | 2.398 | 142.13 | -1.437 |
|  | C41-H43···O31 | 2.385 | 131.37 | -1.823 |
|  | C44-H45···O58 | 2.412 | 147.15 | -1.372 |
|  | C44-H45···O62 | 2.308 | 129.08 | -2.024 |
|  | C44-H46···O85 | 2.428 | 164.49 | -1.205 |
|  | C47-H48···O72 | 2.298 | 145.26 | -1.845 |
|  | C47-H49···O22 | 2.415 | 138.77 | -1.578 |
|  | C50-H51···O78 | 2.455 | 156.19 | -1.185 |
|  | C50-H52···O13 | 2.387 | 162.49 | -1.430 |
| DAP-1 | C31-H32···O12 | 2.360 | 139.52 | -1.734 |
|  | C31-H33···O40 | 2.356 | 155.71 | -1.645 |
|  | C34-H35···O6 | 2.210 | 155.03 | -2.470 |
|  | C34-H36···O8 | 2.286 | 171.06 | -1.890 |
|  | C42-H43···O20 | 2.360 | 139.51 | -1.734 |
|  | C42-H44···O50 | 2.356 | 155.72 | -1.645 |
|  | C45-H46···O29 | 2.210 | 155.03 | -2.470 |
|  | C45-H47···O38 | 2.286 | 171.05 | -1.890 |
|  | C54-H55···O84 | 2.360 | 139.52 | -1.734 |
|  | C54-H56···O61 | 2.356 | 155.72 | -1.644 |
|  | C57-H58···O75 | 2.210 | 155.03 | -2.470 |
|  | C57-H59···O73 | 2.286 | 171.06 | -1.890 |
| TAP-1 | C32-H33···O4 | 2.397 | 118.05 | -1.778 |
|  | C32-H34···O27 | 2.347 | 150.22 | -1.667 |
|  | C35-H36···O79 | 2.223 | 160.39 | -2.314 |
|  | C35-H37···O91 | 2.405 | 143.73 | -1.392 |
|  | C43-H44···O62 | 2.397 | 118.05 | -1.778 |
|  | C43-H45···O82 | 2.347 | 150.23 | -1.667 |
|  | C46-H47···O23 | 2.223 | 160.39 | -2.314 |
|  | C46-H48···O55 | 2.405 | 143.73 | -1.392 |
|  | C57-H38···O29 | 2.421 | 119.92 | -1.622 |
|  | C46-H48···O55 | 2.405 | 143.73 | -1.392 |
|  | C57-H38···O29 | 2.421 | 119.92 | -1.622 |
|  | C57-H49···O84 | 2.421 | 119.93 | -1.622 |
|  | C58-H39···O63 | 2.254 | 155.27 | -2.135 |
|  | C58-H50···O5 | 2.254 | 155.27 | -2.135 |

Table S23. Hydrogen bonds and highest bond energy along different C-H directions in the cage frame of PAP-H2, DAP-2, DAP-O2 and TAP-2.

| Compd | C-H···O | d (H···Y) [Å] | Angle [°] | E [kcal·mol^-1^] |
| --- | --- | --- | --- | --- |
| PAP-H2 | C35-H36···O56 | 2.390 | 175.96 | -1.437 |
|  | C35-H37···O87 | 2.474 | 140.62 | -1.123 |
|  | C38-H39···O74 | 2.261 | 155.86 | -1.957 |
|  | C38-H40···O80 | 2.353 | 164.53 | -1.578 |
|  | C41-H42···O29 | 2.458 | 160.39 | -1.214 |
|  | C41-H43···O17 | 2.597 | 136.11 | -0.837 |
|  | C44-H45···O25 | 2.305 | 145.19 | -1.957 |
|  | C44-H46···O24 | 2.578 | 108.11 | -1.2054 |
|  | C47-H48···O77 | 2.391 | 127.96 | -1.435 |
| DAP-2 | C23-H26···O59 | 2.385 | 141.08 | -1.555 |
|  | C23-H25···O22 | 2.463 | 156.77 | -1.1552 |
|  | C24-H27···O43 | 2.460 | 170.73 | -1.089 |
|  | C24-H28···O40 | 2.275 | 152.42 | -2.046 |
|  | C34-H36···O33 | 2.463 | 156.78 | -1.152 |
|  | C34-H37···O14 | 2.384 | 141.08 | -1.555 |
|  | C35-H38···O2 | 2.460 | 170.73 | -1.089 |
|  | C35-H39···O4 | 2.275 | 152.43 | -2.046 |
|  | C47-H49···O45 | 2.463 | 156.78 | -1.152 |
|  | C47-H50···O69 | 2.385 | 141.07 | -1.555 |
|  | C48-H51···O83 | 2.460 | 170.73 | -1.089 |
| DAP-O2 | C27-H28···O47 | 2.566 | 161.90 | -0.788 |
|  | C27-H29···O69 | 2.480 | 133.82 | -1.067 |
|  | C30-H31···O70 | 2.541 | 174.12 | -0.828 |
|  | C30-H32···O59 | 2.230 | 154.61 | -2.314 |
|  | C33-H34···O5 | 2.359 | 160.34 | -1.578 |
|  | C33-H35···O85 | 2.283 | 153.79 | -2.002 |
|  | C36-H37···O69 | 2.437 | 153.54 | -1.212 |
|  | C36-H38···O58 | 2.453 | 142.19 | -1.120 |
|  | C39-H41···O9 | 2.318 | 141.64 | -1.912 |
|  | C39-H40···O49 | 2.346 | 169.49 | -1.555 |
|  | C42-H43···O14 | 2.742 | 106.66 | -0.612 |
|  | C42-H44···O22 | 2.343 | 145.04 | -1.622 |
| TAP-2 | C64-H65···O40 | 2.265 | 150.31 | -2.069 |
|  | C64-H66···O75 | 2.452 | 148.15 | -1.1409 |
|  | C67-H68···O12 | 2.346 | 141.00 | -1.5559 |
|  | C67-H69···O48 | 2.404 | 156.55 | -1.355 |
|  | C85-H86···O24 | 2.265 | 150.31 | -2.069 |
|  | C85-H87···O15 | 2.452 | 148.15 | -1.143 |
|  | C88-H89···O72 | 2.346 | 140.99 | -1.555 |
|  | C88-H90···O31 | 2.404 | 156.55 | -1.357 |
|  | C91-H62···O2 | 2.164 | 149.71 | -2.649 |
|  | C91-H83···O54 | 2.164 | 149.71 | -2.649 |
|  | C92-H63···O28 | 2.205 | 147.66 | -2.4709 |

Table S24. Hydrogen bonds and highest bond energy along different C-H directions in the cage frame of PAP-H4, PAP-M4, DAP-4, DAP-M4 and TAP-4.

| Compd | C-H···O | d (H···Y) [Å] | Angle [°] | E [kcal·mol^-1^] |
| --- | --- | --- | --- | --- |
| PAP-H4 | C104-H105···O4 | 2.321 | 163.11 | -1.801 |
|  | C104-H106···O40 | 2.479 | 141.97 | -1.285 |
|  | C107-H108···O5 | 2.526 | 125.62 | -1.109 |
|  | C107-H109···O47 | 2.301 | 139.27 | -1.957 |
|  | C110-H111···O12 | 2.325 | 163.37 | -1.779 |
|  | C110-H112···O94 | 2.485 | 136.99 | -1.252 |
|  | C116-H117···O35 | 2.535 | 134.02 | -1.033 |
|  | C116-H118···O52 | 2.467 | 125.89 | -1.263 |
|  | C119-H120···O78 | 2.506 | 174,72 | -0.913 |
|  | C119-H121···O27 | 2.351 | 156.91 | -1.622 |
| PAP-M4 | C28-H29···O54 | 2.387 | 139.49 | -1.459 |
|  | C28-H30···O48 | 2.569 | 130.59 | -0.859 |
|  | C28-H31···O3 | 2.411 | 171.54 | -1.388 |
|  | C32-H33···O44 | 2.514 | 127.36 | -1.096 |
|  | C32-H34···O103 | 2.527 | 131.88 | -1.100 |
|  | C35-H36···O8 | 2.621 | 109.22 | -1.138 |
|  | C35-H37···O89 | 2.320 | 162.10 | -1.823 |
|  | C38-H39···O110 | 2.433 | 156.11 | -1.227 |
|  | C38-H40···O70 | 2.290 | 158.81 | -1.979 |
|  | C41-H42···O87 | 2.359 | 142.19 | -1.511 |
|  | C41-H43···O22 | 2.462 | 129.38 | -1.337 |
| DAP-4 | C41-H42···O86 | 2.321 | 140.99 | -1.912 |
|  | C44-H45···O10 | 2.317 | 149.67 | -1.8237 |
|  | C44-H46···O76 | 2.572 | 174.23 | -0.721 |
|  | C56-H57···O92 | 2.321 | 141.05 | -1.912 |
|  | C56-H58···O65 | 2.515 | 158.22 | -0.964 |
|  | C59-H60···O28 | 2.311 | 150.36 | -1.868 |
|  | C59-H61···O48 | 2.573 | 174.39 | -0.719 |
|  | C68-H69···O38 | 2.343 | 140.52 | -1.779 |
|  | C68-H70···O78 | 2.509 | 157.11 | -0.987 |
|  | C71-H72···O115 | 2.316 | 149.65 | -1.845 |
|  | C71-H73···O63 | 2.528 | 173.79 | -0.857 |
| DAP-M4 | C74-H75···O61 | 2.269 | 157.65 | -2.047 |
|  | C74-H76···O105 | 2.329 | 166.63 | -1.689 |
|  | C77-H78···O64 | 2.312 | 150.54 | -1.868 |
|  | C77-H79···O50 | 2.434 | 151.90 | -1.281 |
|  | C80-H81…O118 | 2.727 | 114.65 | -0.636 |
|  | C80-H82···O117 | 2.515 | 110.87 | -1.288 |
|  | C83-H84···O106 | 2.252 | 149.04 | -2.225 |
|  | C83-H85···O103 | 2.341 | 160.40 | -1.622 |
|  | C86-H87···O109 | 2.600 | 141.38 | -0.730 |
|  | C92-H93···O7 | 2.392 | 132.58 | -1.600 |
|  | C92-H94···O35 | 2.406 | 144.26 | -1.459 |
|  | C92-H95···O22 | 2.376 | 139.61 | -1.622 |
| TAP-4 | C107-H108···O91 | 2.384 | 150.10 | -1.417 |
|  | C107-H109···O70 | 2.411 | 145.41 | -1.348 |
|  | C110-H111···O19 | 2.374 | 163.45 | -1.484 |
|  | C110-H112···O94 | 2.559 | 114.97 | -1.138 |
|  | C117-H118···O39 | 2.384 | 150.11 | -1.417 |
|  | C117-H119···O8 | 2.411 | 145.11 | -1.348 |
|  | C120-H121···O81 | 2.374 | 163.46 | -1.484 |
|  | C120-H122···O24 | 2.349 | 147.76 | -1.600 |
|  | C123-H105···O20 | 2.444 | 115.18 | -1.622 |
|  | C124-H106···O40 | 2.352 | 149.87 | -1.555 |
|  | C124-H116···O92 | 2.352 | 149.88 | -1.555 |

For the four PEMs with K^+^ (PAP-H2, DAP-2, DAP-O2, TAP-2), The average bond energy of C-H···O hydrogen bonds in the cage frame of PAP-H2 is 1.42 kcal mol^-1^, which is close to that of C-H···O hydrogen bond in the cage frame of DAP-2 (1.41 kcal mol^-1^). The average bond energy of C-H···O hydrogen bond in the cage frame larger than DAP-O2 (1.38 kcal mol^-1^), while the average bond energy of C-H···O type hydrogen bond in the above three cage frames is lower than that of C-H···O type hydrogen bond in the TAP-2 cage frame (1.82 kcal mol^-1^).

As can be seen from **Table S23**, the number of C-H···O type hydrogen bonds with the energy greater than 1.00 kcal mol^-1^ in PAP-H2, DAP-2, DAP-O2 and TAP-2 is 10, 12, 10 and 16, respectively. The maximum bond energy of C41-H43 hydrogen bond in the PAP-H2 cage frame is 0.84 kcal·mol^-1^, which is less than 1.00 kcal mol^-1^. The maximum bond energies of hydrogen bonds in the direction of C27-H30 and C30-H31 in the DAP-O2 cage frame are 0.79 kcal mol^-1^ and 0.82 kcal mol^-1^, respectively, indicating the weakest buffering effect on frictional stimuli along this direction. Therefore, the friction sensitivity values of PAP-H2 and DAP-O2 are lower than those of DAP-2 and TAP-2. Compared with DAP-2, both the maximum bond energy along each C-H bond direction and the number of C-H···O type hydrogen bonds with energy greater than 1.00 kcal mol^-1^ are superior to DAP-2. In addition, the A-position cations in the TAP-2 cage frame are more spherical than the A-position cations in the DAP-2 cage frame. Therefore, the friction sensitivity of TAP-2 is more insensitive than that of DAP-2 influenced by a combination of multiple factors. Although the average bond energy of DAP-O2 is slightly less than that of PAP-H2, the A-site cations in DAP-O2 are closer to sphere than the A-site cations in PAP-2, and are therefore more insensitive to frictional stimuli.

For the five PEMs with NH_4_^+^ (PAP-H4, PAP-M4, DAP-4, DAP-M4, and TAP-4), The average bond energies of C-H···O type hydrogen bonds in the cage frame are 1.40 kcal mol^-1^, 1.36 kcal mol^-1^, 1.40 kcal mol^-1^, 1.50 kcal mol^-1^ and 1.50 kcal mol^-1^, respectively. The C-H···O hydrogen bonds in TAP-4 cage framework are superior to the those of the other four PEMs in terms of average bond energy, the C-H···O hydrogen bonds is all greater than 1.00 kcal mol^-1^**(Tables S24)**. Meanwhile, the A-site cations in TAP-4 are closer to sphere than the A-site cations in other PEMs. Besides, both the average bond energy of C-H···O type hydrogen bonds and the sphericity of A-site cations in DAP-4 and DAP-M4 cage frameworks are superior to those in PAP-H4 and PAP-M4, which are more insensitive to frictional stimuli.

**
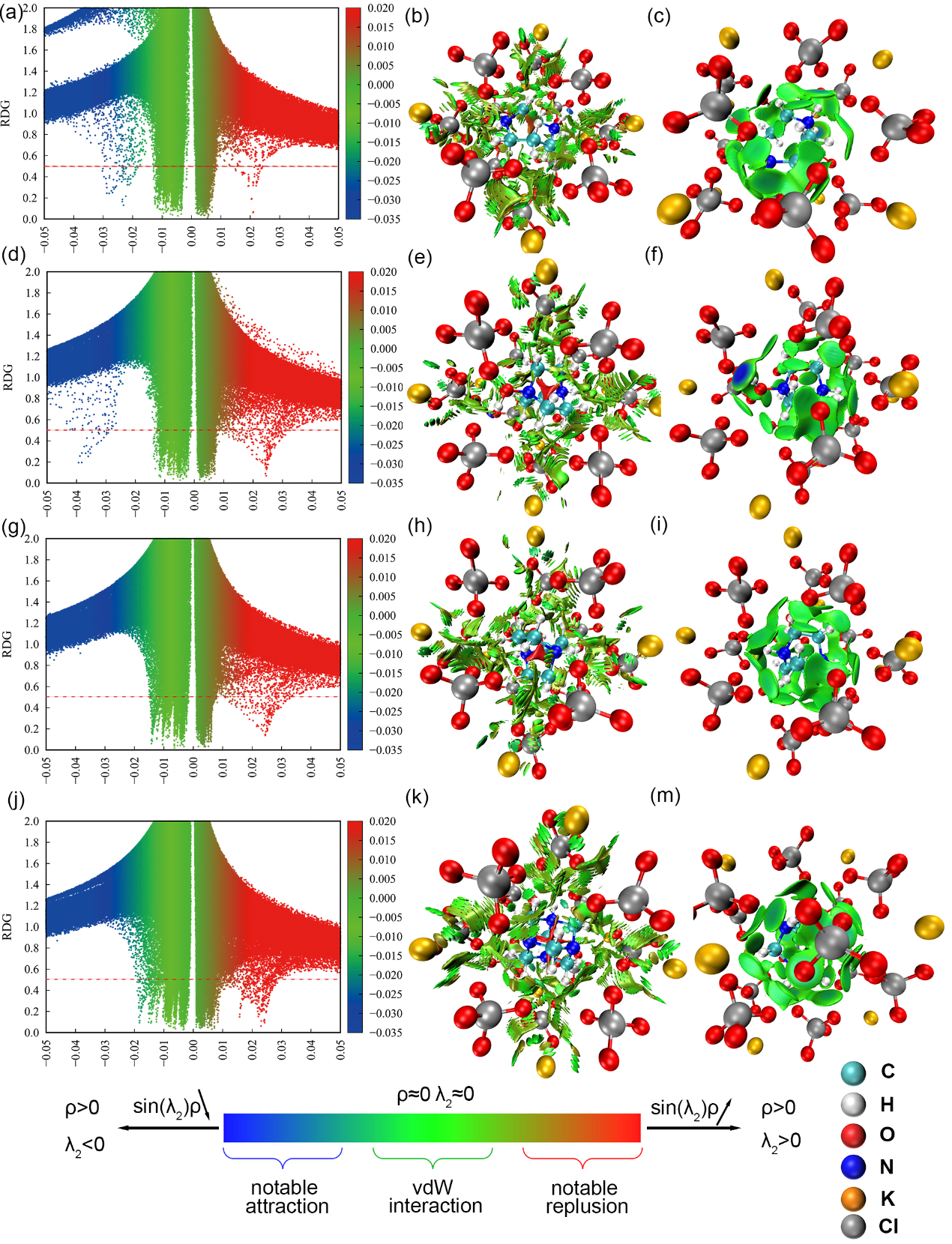
**

Figure S26. IGMH analysis of B-site (K^+^) PEMs. Plots of RDG vs sin(*λ*_2_)*ρ*: (a) PAP-H2, (d) DAP-O2, (g) DAP-2 and (j) TAP-2; Gradient isosurfaces (*s*^pro^=0.25) of (b) PAP-H2, (e) DAP-O2, (h) DAP-2 and (k) TAP-2; Sin(*λ*_2_)*ρ* colored isosurfaces of δ_g_^inter^=0.005 a.u. of (c) PAP-H2, (f) DAP-O2, (i) DAP-2 and (m) TAP-2.

**
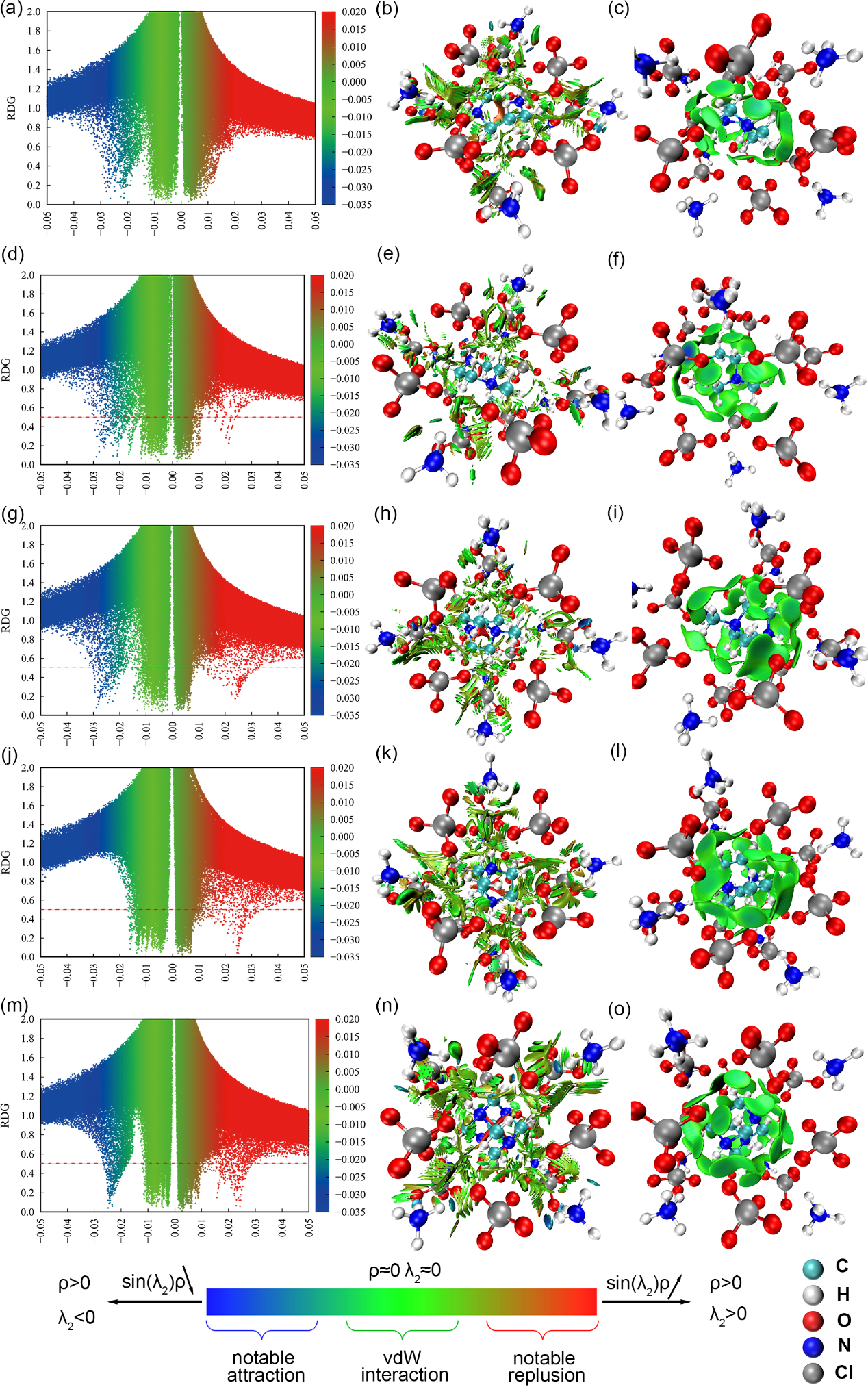
**

Figure S27. IGMH analysis of B-site (NH_4_^+^) PEMs. Plots of RDG vs sin(*λ*_2_)*ρ*: (a) PAP-H4, (d) PAP-M4, (g) DAP-M4, (j) DAP-4 and (m) TAP-4; Gradient isosurfaces (*s*^pro^=0.25) of (b) PAP-H4, (e) PAP-M4, (h) DAP-M4, (k) DAP-4 and (n) TAP-4; *Sign(λ_2_)ρ* colored isosurfaces of δ_g_^inter^=0.005 a.u. of (c) PAP-H4, (f) PAP-M4, (i) DAP-M4, (l) DAP-4 and (o) TAP-4.

From the color bar in **Figure S26**, it can be seen that the blue region has a large ρ (r) and sign(λ_2_)=-1, exhibiting strong and attractive weak interactions. The most common feature that fits this characteristic is hydrogen bonding, including strong halogen bonds and other interactions. The small value of *ρ*(r) in the green area indicates weak interaction strength, and the van der Waals interaction region conforms to this characteristic. Due to the low electron density in this region, the value of sign(λ_2_) is relatively unstable, so it can be positive or negative. The red region *ρ*(r) is relatively large and sign(λ_2_)=+1, corresponding to a strong steric hindrance effect region that appears in the ring and cage, generating tension and causing mutual exclusion between atoms around the red isosurface.

Based on the above rules, the scatter plots of sin (λ_2_) ρ vs RDG for the three types of PEMs was analyzed. From **Figure S26a**, it can be seen that the leftmost spike represents the strongest hydrogen bonding between the A-site cation and the surrounding framework among the four PEMs. Combined with its corresponding contour filling diagram, the position with the strongest hydrogen bonding can be clearly identified. In PAP-H2, the strongest hydrogen bond is located at the N-H ··O at both ends of the piperazine cation, while the strongest hydrogen bond is located at the O-H ··· O in DAP-O2. The *ρ*(r) of the remaining spikes on the left side of PAP-H2 and DAP-O2 are all less than 0.015, representing N-H ··O hydrogen bonds. The *ρ*(r) values of the leftmost spike in DAP-2 and TAP-2 are 0.014 and 0.019, respectively, both lower than those of the leftmost spike in PAP-H2 and DAP-O2, indicating the difference in the strongest hydrogen bond strength between the A-site cation and the surrounding framework among the four PEMs.

Compared the widths of spikes whose ρ(r) are within 0.000~0.015 in the four PEMs scatter plots, it can be found that the spike of TAP-2 occupies a wider width, indicating a wider range of weak hydrogen bonds. For the four PEMs, the red spike on the far right represents the repulsive force inside the annular or cage, which can be observed in the isosurface map at the isovalue of 0.5. The difference in *ρ*(r) values of the rightmost spike of the four PEMs indicates that the high piperazine cation has the least repulsive force, while the dispersion degree of the rightmost spike indicates that the distribution order of the strong repulsive force of the four A-position cations is TAP-2>DAP-2≈DAP-O2>PAP-H2. The *ρ*(r) values of the four peaks on the right side of PEM indicate that the repulsive force is greater in TAP-2, and to some extent, the interaction is stronger.

The IGMH isosurface analysis of A-site cations and anion frames of the four PEMs shows that there are a lot of weak interactions between A-site cations and anion frames, and the position of the strongest mutual attraction is clearly displayed. The distribution of weak interaction around A-site cations is more uniform in TAP-2 and DAP-2 than in DAP-O2 and PAP-H2. The spatial distribution sphericity order of weak interactions around A-site cations is TAP-2>DAP-2>DAP-O2>PAP-H2, which is consistent with the strength and spatial distribution of weak hydrogen bonds. The same situation also exists in PEMs with NH_4_^+^ at the B site. The corresponding RDG scatter plots and contour surface coloring map were shown in **Figure S27**.

**Calculation of detonation performance**

The constant-volume combustion energy (∆_c_*U*) for all samples was measured using an oxygen bomb calorimeter (IKA-C2000, Germany). The standard molar enthalpy of combustion (∆_c_*H*θ m determined at 293.15 K) of all samples was calculated based on ∆_c_*U* (Equation S11). The standard enthalpy of formation (∆_f_*H*θ m) of explosives was calculated from ∆_c_*H*θ m (Equations S13-S15) according to combustion reaction equations of explosives (Table S26). The heats of formation (HOF) and the corresponding results were shown in Table S27.

$\Delta_{\text{c}}H_{\text{m}}^{\theta}=\Delta_{\text{c}}U+\Delta nRT$ (S13)

Δ*n*=*n*_g_(products)-*n*_g_(reactants), *n*_g_ is the sum of the total moles of gas in the product or reactant, R=8.314 J mol^-1^ K^-1^, *T*=298.15 K.

$\Delta_{\text{c}}H_{^{\text{m}}}^{\theta}=\sum n\Delta_{\text{f}}H_{\text{m (production)}}^{\theta}+\sum n\Delta_{\text{f}}H_{\text{m (reaction)}}^{\theta}$ (S14)

$\Delta{}_{\text{f}}H_{\text{m (explosive)}}^{\theta}=\sum n\Delta_{\text{f}}H_{\text{m (production)}}^{\theta}-n\Delta_{\text{f}}H_{\text{m (O}_{2}\text{)}}^{\theta}$ (S15)

Table S25. Heats of formation of combustion and detonation products.

| Compd | H2O(l) | H2O(g) | CO2(g) | | CO(g) | Na2O(s) | K2O(s) |
| --- | --- | --- | --- | --- | --- | --- | --- |
| HOF (KJ mol^-1^) | -286 | −242 | −393 | | −110.5 | −416 | −361 |
| Compd | KCl(s) | NaCl(s) | RbCl | Cl2(g) | | HCl(g) | Rb2O(g) |
| HOF (KJ mol^-1^) | -437 | -411 | -435 | 0 | | -92 | -339 |

Table S26. Combustion reaction equations of explosives.

| Compd | Combustion reaction equations | |
| --- | --- | --- |
| TAP-1 | C_6_H_14_N_4_O_12_Cl_3_Na_(cr)_+3O_2(g)_ → 6CO_2_+6H_2_O_(l)_+2N_2(g)_+NaCl_(s)_+2HCl(H_2_O)_(l)_ |  |
| TAP-2 | C_6_H_14_N_4_O_12_Cl_3_K_(cr)_+3O_2(g)_ → 6CO_2_+7H_2_O_(l)_+2N_2(g)_+KCl_(cr)_+2HCl(H_2_O)_(l)_ |  |
| TAP-3 | C_6_H_14_N_4_O_12_Cl_3_Rb_(cr)_+3O_2(g)_ → 6CO_2_+7H_2_O_(l)_+2N_2(g)_+RbCl+2HCl(H_2_O)_(l)_ |  |
| TAP-4 | C_6_H_18_N_5_O_12_Cl_3(cr)_+3.75O_2(g)_ → 6CO_2_(g)+7.5H_2_O(l)+2.5N_2(g)_+3HCl(H_2_O)_(l)_ |  |
| DAP-4 | C_6_H_18_N_3_O_12_Cl_3(cr)_+3.75O_2(g)_ → 6CO_2_(g)+7.5H_2_O(l)+1.5N_2(g)_+3HCl(H_2_O)_(l)_ |  |

Table S27. The results of standard molar enthalpy of combustion and formation.

| Compd | *T*_d_ [°C]^a)^ | Δ_c_*H* [KJ g^-1^]^c)^ | Δ_c_*U* [KJ mol^-1^]^b)^ | Δ_r_*H* [KJ mol^-1^]^c)^ | Δ_f_*H* [KJ mol^-1^]^d)^ |
| --- | --- | --- | --- | --- | --- |
| TAP-1 | 197 | 12.22 | -5663.41 | -5643.58 | 974.25 |
| TAP-2 | 213 | 10.40 | -4989.69 | -4969.86 | 275.18 |
| TAP-3 | 200 | 9.18 | -4826.68 | -4806.85 | 113.32 |
| TAP-4 | 203 | 13.26 | -6080.50 | -6061.29 | 1279.58 |
| DAP-4 | 369 | 11.43 | -4920.00 | -4882.20 | 122.80 |

^a)^ Onset decomposition temperature; ^b)^ Constant-volume combustion energies; ^c)^ Standard molar enthalpy of combustion; ^d)^ Standard molar enthalpy of formation; ^e)^ Oxygen balances based on C_a_H_b_N_c_O_d_M_e_Cl_f_: OB = 1600[d-2a-(b-f +e)/2]/M.

Detonation velocity (*D*), detonation pressure (*P*) and heat of Detonation (*Q*) are the main parameters for evaluating explosives. The detonation performance of TDPIs and DAPs were predicted by the modified Kamlet-Jacbos (K-J) equations^[15]^ (Equations S14-S16) and the EXPLO5_v6.05.02 program.^[13]^ The reliability of results was verified using two different methods. Before using K-J equations, the equation of detonation needs to be determined. Actually, the detonation products of explosives are complicated. According to literature, the detonation equations could be simplified as described in Table S28.^[16]^

Table S28. Detonation reaction equations of explosives.

| Compd | explosive reaction equations |
| --- | --- |
| TAP-1 | C_6_H_14_N_4_O_12_Cl_3_Na→3.25C_(s)_+2.75CO_2_+6.50H_2_O(g)+N_2_+0.5Na_2_O+0.5Cl_2_+1.00HCl(g) |
| TAP-2 | C_6_H_14_N_4_O_12_Cl3_K_→3.25C_(s)_+2.75CO_2_+6.50H_2_O(g)+N_2_+0.5K_2_O+0.5Cl_2_+1.00HCl(g) |
| TAP-3 | C_6_H_14_N_4_O_12_Cl_3_Rb→3.25C_(s)_+2.75CO_2_+6.50H_2_O(g)+N_2_+0.5Rb_2_O+0.5Cl_2_+1.00HCl(g) |
| TAP-4 | C_6_H_18_N_4_O_12_Cl_3_→4.00C(s)+2.00CO_2_+8.00H_2_O(g)+2.50N_2_+0.50Cl_2_+2.00HCl(g) |
| DAP-5 | C_6_H_18_N_2_O_12_Cl_3_→4.00C(s)+2.00CO_2_+8.00H_2_O(g)+1.50N_2_+0.50Cl_2_+2.00HCl(g) |

$D=1.01\left(NM^{1/2}Q^{1/2}\right)^{1/2}\left(1+1.30\rho_{0}\right)$ (S16)

$P=1.55\rho_{0}^{2}M^{1/2}Q^{1/2}$ (S17)

$Q=\frac{\Delta_{f}H\text{(detonation production)-}\Delta_{f}H\text{(explosive)}}{\text{formula weight of explosive}}$ (S18)

where *D* is the detonation velocity (km s^-1^), *P* is the detonation pressure (GPa) and *Q* is the heat of detonation (Kcal g^-1^). *ρ* represents the density of explosive (g cm^-1^), *N* is the moles of detonation gases per gram of explosive and *M* is the average molecular weight of the gases (g mol^-1^).

Table S29. The calculation results of detonation parameters by K-J equations and EXPLO5.

| Compd | *ρ* [g cm^-3^]^a)^ | *D* [km s^-1^]^b)^ | | *P* [GPa]^c)^ | | *Q* [KJ mol^-1^]^d)^ | | *OB* [%]^e)^ |
| --- | --- | --- | --- | --- | --- | --- | --- | --- |
|  |  | K-J | EXPLO5 | K-J | EXPLO5 | K-J | EXPLO5 |  |
| TAP-1 | 1.99 | 8.48 | 9.19 | 33.77 | 43.09 | -4131.69 | -3849.09 | -20.71 |
| TAP-2 | 2.09 | 8.68 | 8.74 | 36.37 | 38.47 | -3458.22 | -3201.33 | -20.02 |
| TAP-3 | 2.22 | 8.36 | _ | 34.87 | - | -3294.96 | - | -18.24 |
| TAP-4 | 1.96 | 9.51 | 9.43 | 42.63 | 44.72 | -4185.78 | -3942.94 | -26.17 |
| DAP-4 | 1.90 | 8.57 | 8.63 | 33.69 | 35.44 | -3029.00 | -2836.70 | -27.87 |

1. Crystal density; ^b)^ Detonation velocity; ^c)^ Detonation pressure; ^d)^ Heat of detonation; ^e)^Oxygen balance

**
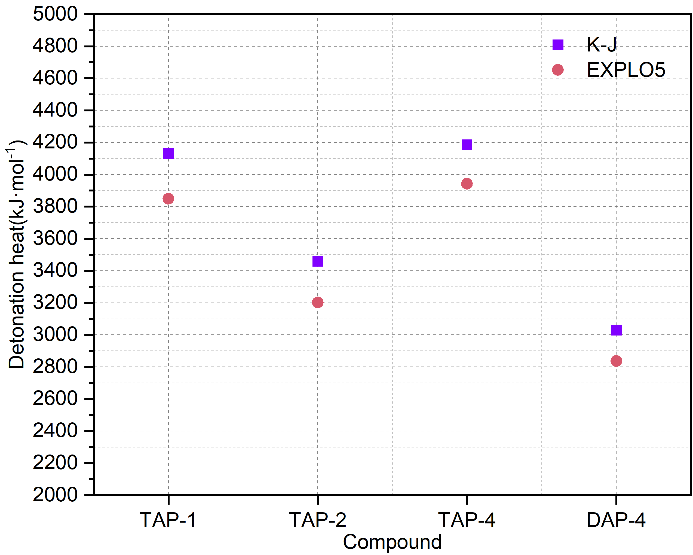
**

Figure S28. Comparison diagram of the calculated detonation velocity by two methods.

**
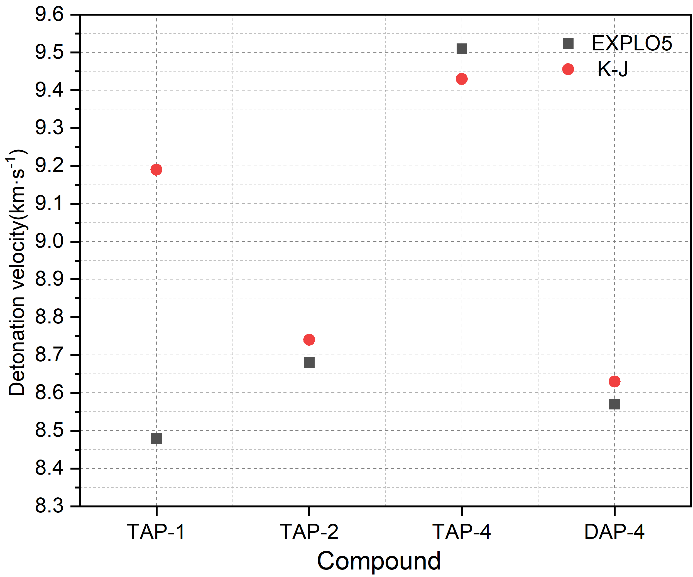
**

Figure S29. Comparison diagram of the calculated detonation pressure by two methods.

**
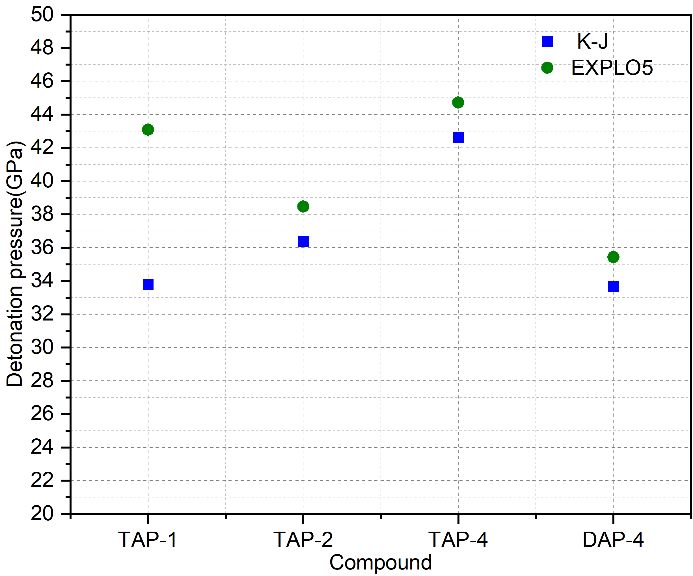
**

Figure S30. Comparison diagram of the calculated heat of detonation by two methods.

Comparing the results of the calculated detonation performance by two methods, the detonation velocity, detonation pressure, and heat of detonation all exhibit good consistency except for TAP-1 (Figure S28-S30). Concretely, the detonation velocity deviation of PEMs (TAP-2, TAP-4, DAP-4) are all less than 0.2 km s^-1^, the detonation pressure deviation is less than 2.2 MPa, and the heat of detonation deviation is less than 200 KJ mol^-1^. All detonation parameter deviation is within the acceptable range. The consistency of the results also indicates the reliability of the calculation. However, the detonation performance of TAP-1 shows significant difference in addition to the detonation heat. As shown in Table S30, it was found that the carbon content in the explosion equation of TAP-1 is significantly lower than that of other PEMs. It also means that the detonation products of TAP-1 generated by EXPLO5 has more gas than those generated by the K-J equation. Therefore, there is a significant difference in the detonation velocity and pressure obtained by the two calculation methods. Finally, the detonation performance of TAP-1 were calculated using the K-J equation.

Table S30. The detonation production generated by EXPLO5.

| Compd | explosive reaction equations |
| --- | --- |
| TAP-1 | 0.48C_(s)_+3.27CH_2_O_2(g)_+0.86CO_2_+2.44H_2_O_(g)_+1.91N_2_+NaCl_(s)_+1.92HCl_(g)_+1.30CO+0.12NH_3(g)_ |
| TAP-2 | 1.28C_(s)_+2.37CH_2_O_2(g)_+0.95CO_2_+2.98H_2_O_(g)_+1.96N_2_+0.5K_2_CO_3(s)_+2.95HCl_(g)_+0.87CO+0.07NH_3(g)_ |
| TAP-4 | 1.29C_(s)_+3.06CH_2_O_2(g)_+0.51CO_2_+3.84H_2_O_(g)_+2.34N_2_+2.91HCl_(g)_+1.00CO+0.26NH_3(g)_+0.12H_2(g)_ |
| DAP-4 | 1.85C_(s)_+2.40CH_2_O_2(g)_+0.74CO_2_+4.76H_2_O_(g)_+1.42N_2_+2.96HCl_(g)_+0.96CO+0.13NH_3(g)_+0.10H_2(g)_ |

**Lead plate perforation test**

In this test, a 500 mg sample was used as the secondary charge to detonate a 5 mm thick lead plate. The detonation performance was evaluated by measuring the perforation diameter of the lead plate. A 220 mg RDX was applied as the transition charge on the upper layer of the secondary charge under soft pressure. Finally, 100 mg of nickel hydrazine azide (NHA) was loaded as the primary explosive into an 8# industrial detonator and ignited using a standard electrical igniter. The primary charge and secondary explosive were compressed under static pressures of 30 MPa and 50 MPa, respectively.

Each sample undergoed two experiments, where the maximum and minimum pore sizes are measured independently, and the average of these values is used to represent the pore size. The maximum value from the two experiments is selected to represent the detonation performance under the given condition.
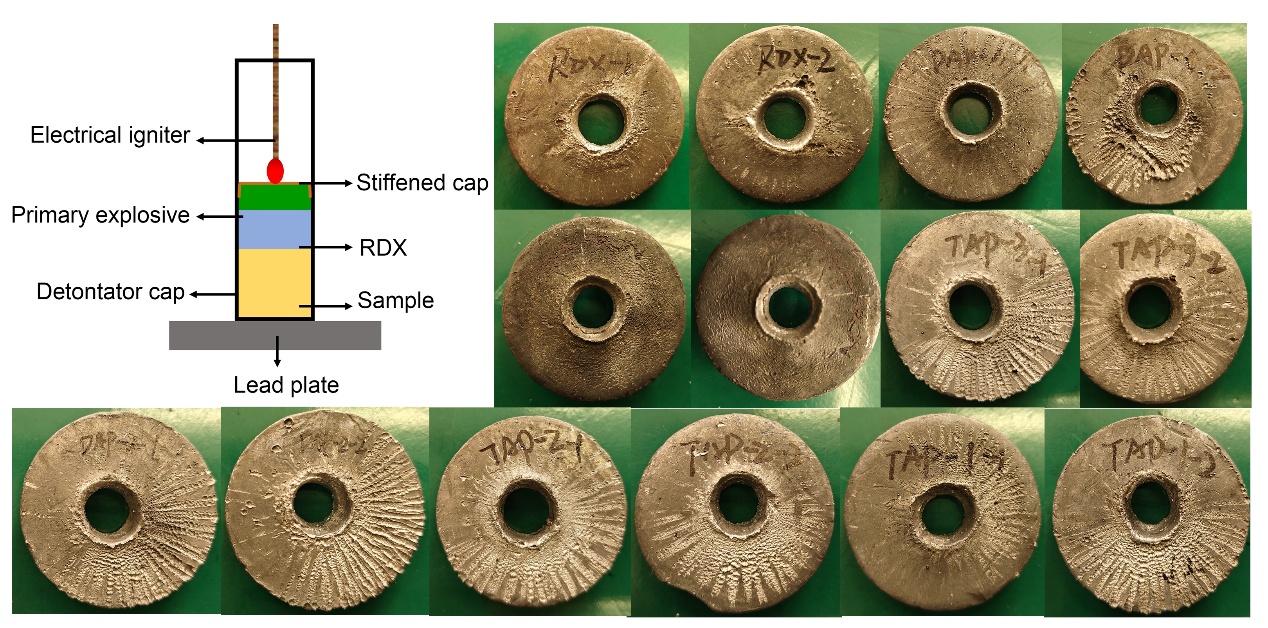


Figure S31. diagram of lead plate experimental apparatus and physical picture of lead plate perforation.

Table S31. minimum, maximum, and average values for lead plate perforation diameter

| Compd | Smaple1 perforation diameter (mm) | | | Sample2 perforation diameter (mm) | | |
| --- | --- | --- | --- | --- | --- | --- |
|  | min | Max | Average | Min | Max | Average |
| RDX | 10.40 | 10.60 | 10.50 | 10.82 | 10.90 | 10.86 |
| DAP-4 | 10.42 | 11.10 | 10.76 | 9.14 | 11.50 | 10.32 |
| TAP-4 | 10.96 | 11.16 | 11.06 | 10.60 | 11.10 | 10.85 |
| TAP-3 | 10.40 | 10.70 | 10.55 | 9.80 | 11.10 | 10.45 |
| TAP-2 | 10.60 | 10.80 | 10.70 | 9.40 | 9.90 | 9.65 |
| DAP-2 | 10.00 | 10.50 | 10.25 | 9.00 | 10.30 | 9.65 |
| TAP-1 | 9.80 | 10.80 | 10.30 | 9.30 | 9.86 | 9.58 |


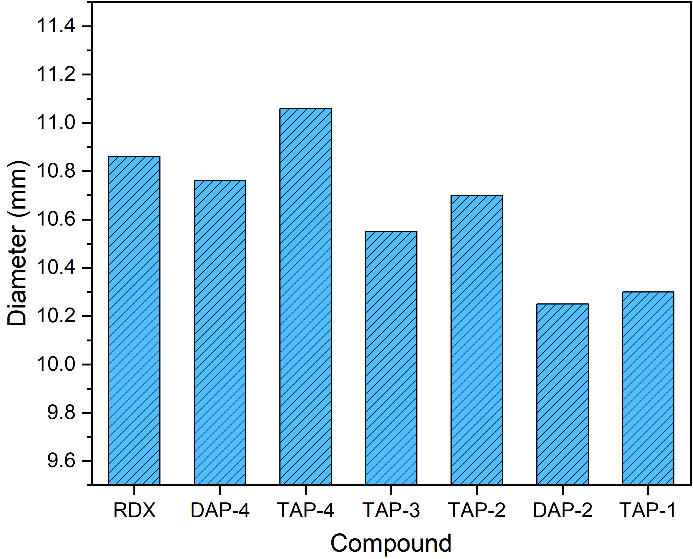


Figure S32. Comparison diagram of lead plate perforation diameter.

As illustrated in the figure 32, under the charging conditions described in this study, the detonation velocities of the compounds follow this order: TAP-4 > RDX > DAP-4 > TAP-2 > TAP-3 > TAP-1 > DAP-2. Although the perforation diameter order of TAPs does not fully align with the calculated detonation velocity order, the results comparing TAP-4 with DAP-4 and TAP-2 with DAP-2 suggest that Urotropine, as an A-site cation, contributes to the increase in detonation velocity.

**References**

[1] Travis W, Glover E N K, Bronstein H, et al. *Chemical Science*, 2016, 7(7): 4548-4556.

[2] Li Z, Yang M, Park J, et al. *Chemistry of Materials*, 2016, 28(1): 284-292.

[3] Kuehne. Thomas D, Iannuzzi. Marcella, Del Ben. Mauro, Rybkin. Vladimir V, Seewald. Patrick, Stein. Frederick, Laino. Teodoro, Khaliullin. Rustam Z, Schuett. Ole, Schiffmann. Florian, Golze. Dorothea, Wilhelm. Jan, Chulkov. Sergey, Bani-Hashemian. Mohammad. Hossein, Weber. Valery, Borstnik. Urban, Taillefumier. Mathieu, Jakobovits. Alice Shoshana, Lazzaro. Alfio, Pabst. Hans, Mueller. Tiziano, Schade. Robert, Guidon. Manuel, Andermatt. Samuel, Holmberg. Nico, Schenter. Gregory K, Hehn. Anna, Bussy. Augustin, Belleflamme. Fabian, Tabacchi. Gloria, Gloess. Andreas, Lass. Michael, Bethune. Iain, Mundy. Christopher. J, Plessl. Christian, Watkins. Matt, VandeVondele. Joost, Krack. Matthias, Hutter. Juerg, *J. Chem. Phys*. 2020, 152(19), 194103.

[4] Vande.Vondele J, Krack. M, Mohamed. F, Parrinello. M, Chassaing. T, Hutter. R, *Comput. Phys. Commun*. 2005, 167(2), 103-128.

[5] VandeVondele. J, Hutter. J, *J. Chem. Phys*. 2007, 127, 114105(1)-114105(9).

[6] Goedecker. S, Teter. M. J, *Phys. Rev. B*. 1996, 54, 1703-1710.

[7] Krack. M, *Theor. Chem. Acc*. 2005, 114, 145-152.

[8] Perdew. J, P. Burke. K, Ernzerhof. M, *Phys. Rev. Lett*. 1996, 77, 3865.

[9] Grimme. S, Antony. J, Ehrlich. S, Krieg. H, *J. Chem. Phys.* 2010, 132, 154104.

[10] Grimme. S, Ehrlich. S, Goerigk. L, *J. Comput. Chem*. 2011;32(7):1456-1465.

[11] Wang. L, Li. J, Cheng. L, *J. Mater. Chem. A*. 2021, 9(26), 14868-14876.

[12] Mendez. F, Gazquez. J. L, *J. Am. Chem. Soc*. 1994, 116(20), 9298-9301.

[13] Tian. Lu, Feiwu. Chen, *J. Comput. Chem*. 2012, 33, 580-592.

[14] M. J. Frisch, G. W. Trucks, H. B. Schlegel, G. E. Scuseria, M. A. Robb, J. R. Cheeseman, G. Scalmani, V. Barone, G. A. Petersson, H. Nakatsuji, X. Li, M. Caricato, A. V. Marenich, J. Bloino, B. G. Janesko, R. Gomperts, B. Mennucci, H. P. Hratchian, J. V. Ortiz, A. F. Izmaylov, J. L. Sonnenberg, Williams, F. Ding, F. Lipparini, F. Egidi, J. Goings, B. Peng, A. Petrone, T. Henderson, D. Ranasinghe, V. G. Zakrzewski, J. Gao, N. Rega, G. Zheng, W. Liang, M. Hada, M. Ehara, K. Toyota, R. Fukuda, J. Hasegawa, M. Ishida, T. Nakajima, Y. Honda, O. Kitao, H. Nakai, T. Vreven, K. Throssell, J. A. Montgomery Jr., J. E. Peralta, F. Ogliaro, M. J. Bearpark, J. J. Heyd, E. N. Brothers, K. N. Kudin, V. N. Staroverov, T. A. Keith, R. Kobayashi, J. Normand, K. Raghavachari, A. P. Rendell, J. C. Burant, S. S. Iyengar, J. Tomasi, M. Cossi, J. M. Millam, M. Klene, C. Adamo, R. Cammi, J. W. Ochterski, R. L. Martin, K. Morokuma, O. Farkas, J. B. Foresman, D. J. Fox, Wallingford, CT 2016.

[12] Emamian. S, Lu. T, Kruse. H, *J. Comput. Chem*. 2019, 40(32), 2868-2881.

[15] Wang. Y, Zhang. J, Su. H, Li. S, Zhang. S, Pang. S, *J*. *Phys. Chem. A*. **2014**, 25, 118.

[16] a) Jichuan. Zhang, Zhenye. Zhu, Mingqing. Zhou, Jiaheng. Zhang, Joseph. P. Hooper, Jean’ne. M. Shreeve, *ACS Appl*. *Mater*. *Interfaces* **2020**, 12, 36; b) Tingwei. Wang, Zhenxin. Yi, Xiaojun. Wang, Wenli. Cao, Shunguan. Zhu, Jianguo. Zhang, *ACS Appl*. *Mater*. *Interfaces* **2022**, 14, 14; c) Ying. Li, Yuteng. Cao, Siwei. Song, Sitong. Chen, Yi. Wang, Kangcai. Wang, Qinghua. Zhang, *Chem*. *Eng*. *J*. **2022**, 442, 136326.
